# Fruit-eating adaptations in a Late Cretaceous oviraptorosaur (Theropoda)

**DOI:** 10.1038/s41467-026-78343-w

**Published:** 2026-10-10

**Authors:** Gregory F. Funston, Thomas W. Dudgeon, D. Jade Simon, Danielle M. Dufault, Clint Boyd, Denver Fowler, Philip J. Currie, David C. Evans

**Affiliations:** 1 Stony Brook University, Department of Anatomical Sciences, Stony Brook, NY USA; 2 Royal Ontario Museum, Department of Natural History, Toronto, ON Canada; 3 University of California, Davis, Department of Earth and Planetary Sciences, Davis, CA USA; 4 University of Toronto, Department of Ecology and Evolutionary Biology, Toronto, ON Canada; 5 Fossil Resource Management Program, North Dakota Geological Survey, Bismarck, ND USA; 6 Badlands Dinosaur Museum, Dickinson Museum Center, Dickinson, ND USA; 7 University of Alberta, Department of Biological Sciences, Edmonton, AB Canada

**Keywords:** Palaeontology, Palaeoecology

## Abstract

The diversification of herbivorous dinosaurs and the rise of angiosperms in the Late Cretaceous are each well established, but the link between them is debated. Broad-scale diversity patterns in the two groups appear not to closely align, and there is no direct evidence of dinosaur adaptation to angiosperm-based diets. Here we describe *Infernogallus jamesi* gen. et. sp. nov., a diminutive caenagnathid oviraptorosaur, and show that these theropods were specialised herbivores that originated alongside the rise of fleshy angiosperm fruits. The nearly complete, articulated skull of *Infernogallus jamesi*—the first for the clade—reveals drastic changes to the adductor musculature organisation, unique in non-avian theropods but converged upon by extant frugivorous parrots. These modifications enabled extensive oral processing of soft food using anteroposterior movement of the mandible and occlusion of ridges and grooves on opposed elements. Altogether, this attests to a highly specialised herbivorous diet in caenagnathids, which likely consisted in large part of fleshy angiosperm fruits, which increased in size and diversity in concert with the radiation of oviraptorosaurs. Caenagnathids thus provide one of the strongest candidates for angiosperm-based diet adaptations in dinosaurs, but their role in the Late Cretaceous proliferation of fruit—which shaped early Cenozoic flora—remains unclear.

## Abstract (web summary)

Dinosaurs coexisted with flowering plants, but it’s unclear whether they ate them. The study presents a new small theropod dinosaur with remarkable jaw adaptations for eating fruit, providing one of the strongest evidence of dinosaur-angiosperm interaction.

## Introduction

Late Cretaceous ecosystems exhibited peaks in diversity for many terrestrial animal clades, including insects^1^, mammals^2–4^, and dinosaurs^5,6^. These evolutionary radiations were likely spurred by the emergence of angiosperm plants^7,8^, and the new niches that opened in response, dubbed the Cretaceous (or Angiosperm) Terrestrial Revolution^9^. Whereas direct links have been drawn between angiosperm evolution and the diversifications of insects, mammals, and birds, the relationship between the radiations of herbivorous non-avian dinosaurs and flowering plants are less clear^5,6,10–12^. Where precise diets are known, angiosperms are not major components, with ferns, cycads, and conifers comprising the bulk of material^13,14^. Nevertheless, the exact diets of many groups of herbivorous dinosaurs remain unknown, particularly the herbivorous theropods like ornithomimids, oviraptorosaurs, and therizinosaurs, making these relationships difficult to test.

Among these herbivorous theropods, oviraptorosaurs in particular are the subject of considerable dietary debate. Initially considered ovivorous^15,16^, later work suggested molluscivory^17^, carnivory^18^, omnivory^19–21^, strict or predominant herbivory^22–25^, granivory^26,27^, frugivory^28,29^, or even crevice-probing^30^. While some of this uncertainty stems from the inherent adaptability of the toothless beak in the later-branching taxa, there is also a major gap in our understanding of their craniomandibular biomechanics, particularly in the clade Caenagnathidae.

Caenagnathids were a predominantly North American group of oviraptorosaurs distinguished by long, low edentulous mandibles that often bear complex ridges and grooves on their occlusal surfaces^16,19,31^; elongate, gracile limbs^23,32,33^; and highly pneumatized skeletons^23,34,35^. This combination of features selects against their preservation in many Late Cretaceous depositional systems in the Western Interior Basin^36,37^, resulting in a fossil record dominated by fragmentary, isolated bones^38^. Although partial skeletons are known^23,32,34,35,39–43^, none have yet enabled analysis of the mandibular adductor musculature, which would elucidate the functions of their unusual mandibles.

Here we describe a new genus and species of caenagnathid from the latest Cretaceous Hell Creek Formation that preserves the first intact cranium known for the group. The bizarre skull has striking adaptations to the temporal and palatal regions related to feeding. Functional analysis of these features together indicates a highly specialised soft-plant herbivorous diet that, based on extant analogues, consisted partially of fruit. Caenagnathids diversified in the Late Cretaceous across Laurasia, and now provide one of the strongest cases for dinosaur-angiosperm interaction via the evolution of frugivory, with ramifications for ecosystem structure preceding the end-Cretaceous mass extinction.

## Results and discussion

### Systematic palaeontology

Theropoda

Oviraptorosauria

Caenagnathidae

Eucaenagnathinae Clade nov.

#### Definition

The most inclusive monophyletic group including *Anzu wyliei* and *Elmisaurus rarus*, but not *Anomalipes zhaoi*.

#### Diagnosis

Eucaenagnathines (meaning ‘true caenagnathids’) are distinguished from other caenagnathids by the morphology of the fused dentaries, with an upturned tomial edge marked with lateral and anterior occlusal ridges, and a midline sulcus bordered by longitudinal lingual ridges separated from the tomial edge by a lingual groove.

**Fig. 1:**
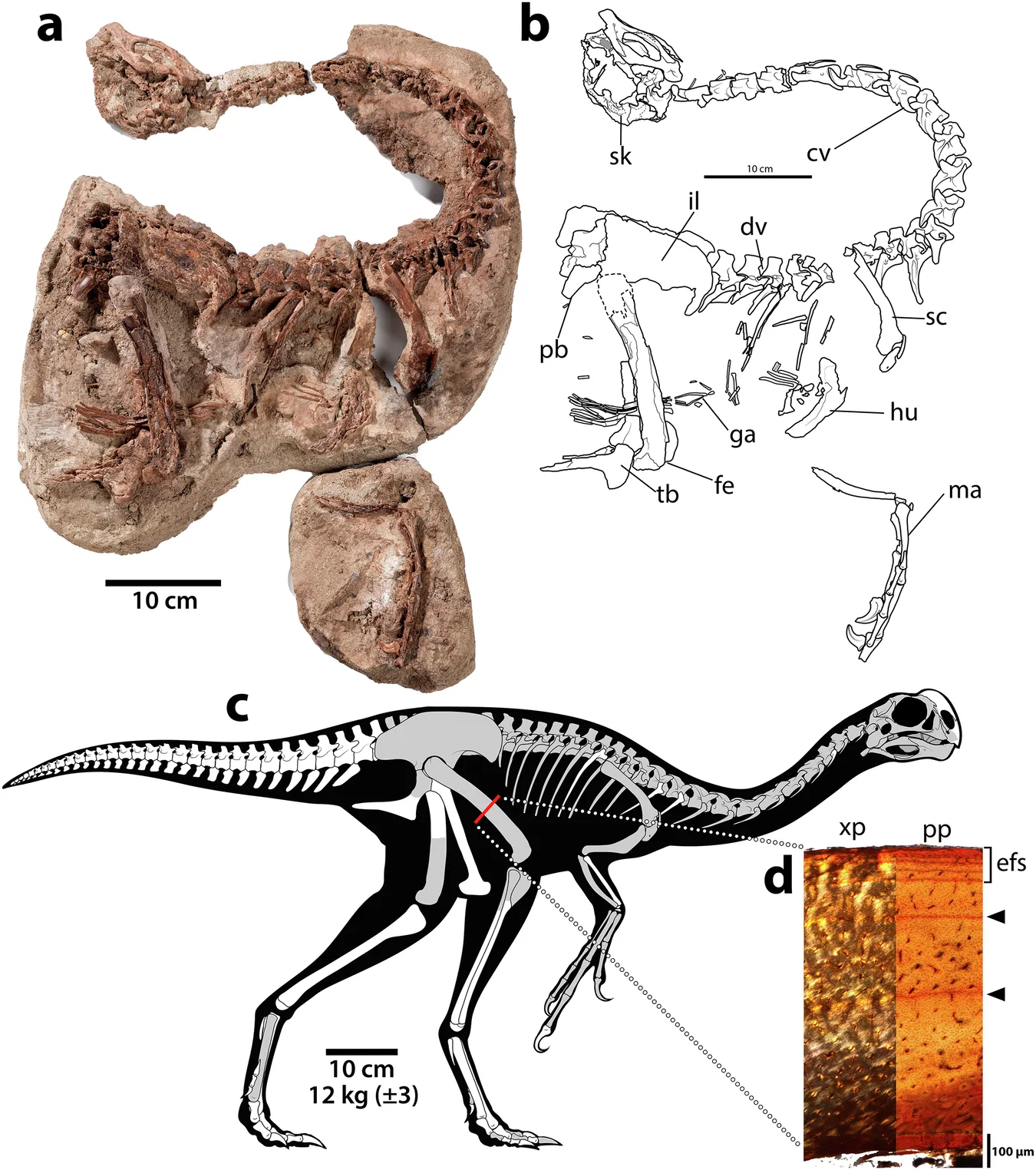
*Infernogallus jamesi* (ROM VP 65050). Photograph (**a**) and line drawing (**b**) of ROM VP 65050. **c** Skeletal reconstruction of *Infernogallus jamesi* based on ROM VP 65050 and other eucaenagnathines (shaded areas are preserved in ROM 65050). **d** Palaeohistological thin section of the femur of ROM VP 65050, showing lines of arrested growth (arrows) and an external fundamental system indicative of advanced age and skeletal maturity. cv cervical vertebrae, dv dorsal vertebrae, efs external fundamental system, fe femur, ga gastralia, hu humerus, il ilium, ma manus, pb pubis, pp plane-polarised light, sc scapulacoracoid, sk skull, tb tibia, xp cross-polarised light. Scale bars as indicated.

**Fig. 2:**
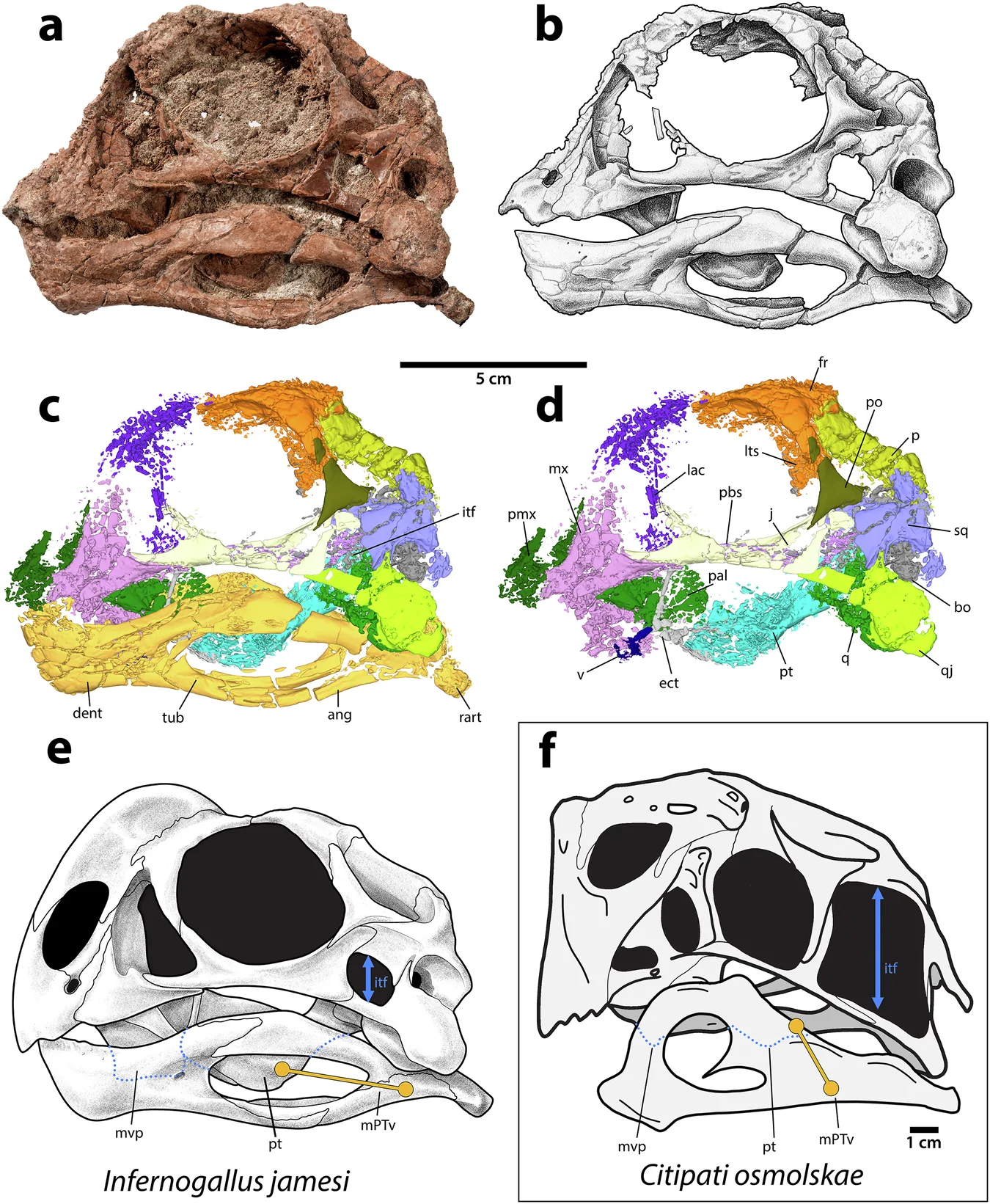
Cranial anatomy of *Infernogallus jamesi* gen. et. sp. nov. Photograph (**a**) and line drawing (**b**) of the skull of ROM VP 65050 in left lateral view. **c** µCT reconstruction of the skull and mandible, with each bone segmented separately (colours) and retrodeformed (see “Methods”). **d** µCT reconstruction with the mandible removed to show the palatal bones, which protrude ventrally below the cheek margin and the mandibular articulation point of the quadrate. **e** idealised reconstruction of the skull and mandible as they would have appeared in life, showing distinctive reduction of infratemporal fenestra (blue arrow), and rearrangement of pterygoid musculature (orange line) compared to archetypal oviraptorid *Citipati osmolskae* (**f**). Dotted blue lines in (**e**, **f**) outline internal features obscured by overlying mandibles. bo basioccipital, ect ectopterygoid, fr frontal, j jugal, lac lacrimal, lts laterosphenoid, mvp maxillovomeral process, mPTv pterygoideus ventralismuscle, mx maxilla, p parietal, pal palatine, pbs parabasiphenoid, pmx premaxilla, po postorbital, pt pterygoid, q quadrate, qj quadratojugal, sq squamosal, v vomer. Scale bar as indicated.

**Fig. 3:**
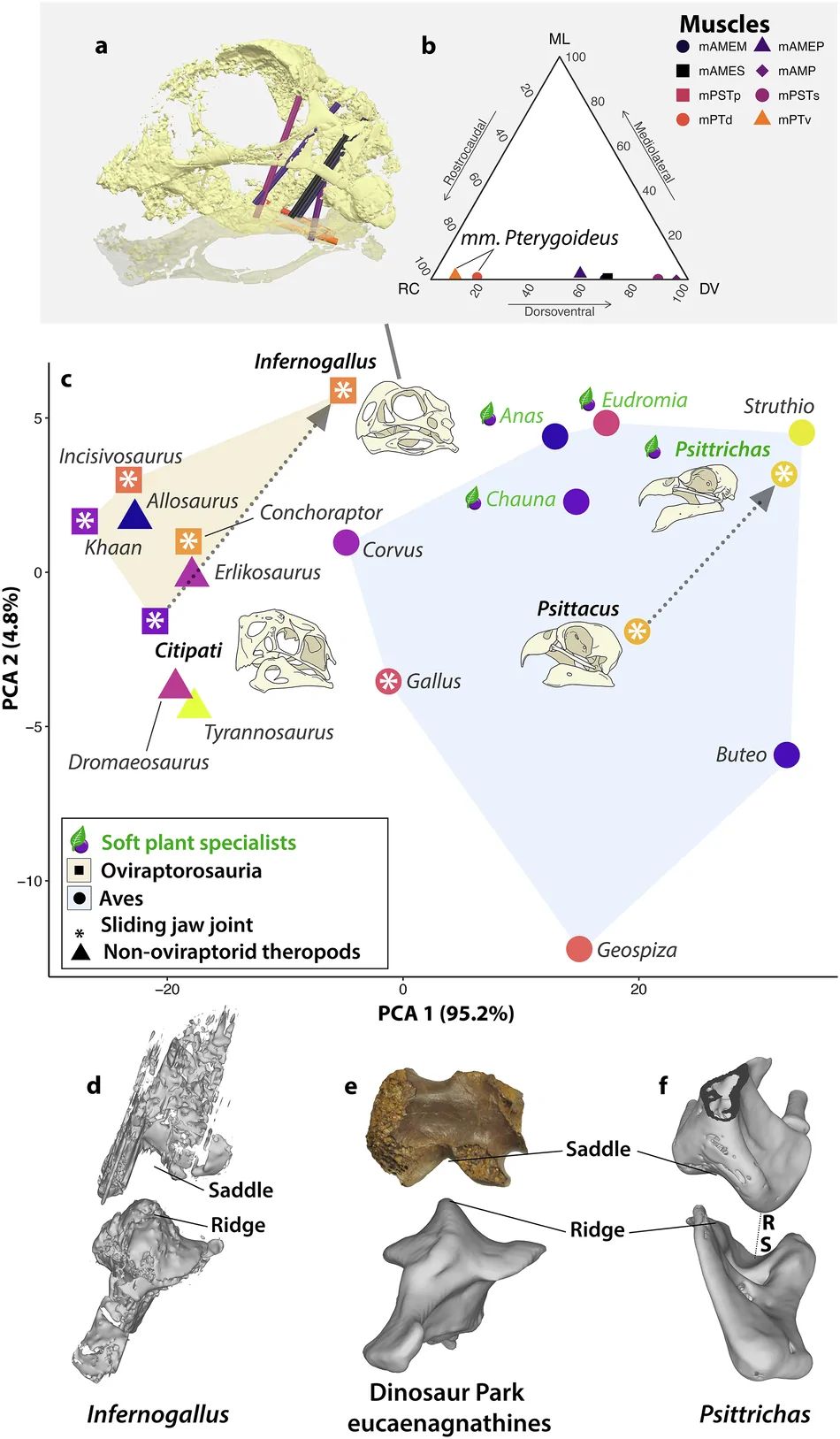
Cranial muscle organisation in caenagnathids. **a** µCT model of the skull and mandible of *Infernogallus*, with three-dimensional adductor muscle reconstructions when the mandible is positioned in the middle of its anteroposterior articular range (see Supplementary Fig. 8 for other configurations). Points are coloured by muscle, colours do not represent additional data. **b** decomposed muscle vector orientations for *Infernogallus*, showing strongly anteroposterior (rostrocaudal) actions of the *pterygoideus* muscles. **c** principal components analysis of total mandibular adductor muscle organisation, summarising muscular vectors for each taxon. Note the close clustering of non-avian theropods, except for *Infernogallus*, which, compared to other oviraptorosaurs (e.g., *Citipati osmolskae*), emphasises anteroposterior motions at the expense of dorsoventral motions. A similar pattern of change (dotted grey arrows) is evident between *Psittacus* and *Psittrichas*, likely related to different oral processing behaviours. Triangles are non-oviraptorosaur theropods, squares are oviraptorosaurs, and circles are avians; points with an asterisk possess a mandibular articulation that enables anteroposterior motion; convex hulls group oviraptorosaurs (orange) and avians (blue). Points are coloured by species, colours do not represent additional data. **d**–**f** posterior view of mandibular articular regions of *Infernogallus*, Dinosaur Park eucaenagnathines, and *Psittrichas*, showing ridge and saddle arrangement enabling anteroposterior sliding of the mandible; images not to scale. mAMEM m. adductor mandibulae externus medialis, mAMEP m. adductor mandibulae externus profundus, mAMES m. adductor mandibulae externus superficialis, mAMP m. adductor mandibulae posterior, mPSTp m. pseudotemporalis profundus, mPSTs m. pseudotemporalis superficialis, mPTd m. pterygoideus dorsalis, mPTv m. pterygoideus ventralis, PCA principal component axis, R ridge, S saddle.

**Fig. 4:**
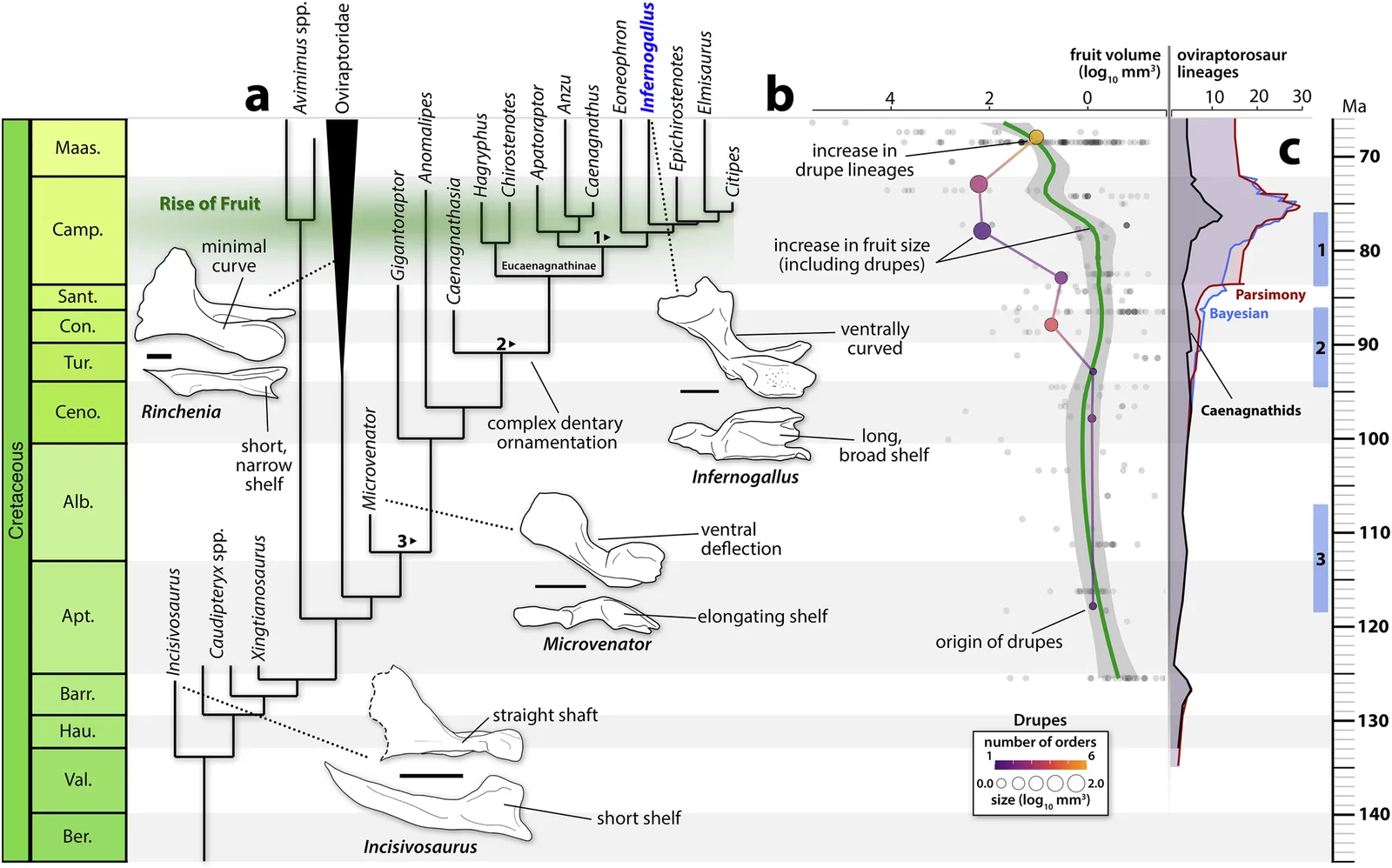
Evolution of Caenagnathidae alongside the rise of fruit. **a** Bayesian phylogeny of Oviraptorosauria, scaled *a posteriori* using a morphological-clock-informed fossilised birth-death (FBD) model. Mean node ages were derived from 100 trees output by the model to scale the branches. Line diagrams of oviraptorosaur pterygoids in right lateral (top) and ventral (bottom) views illustrate early acquisition of derived features in the caenagnathid lineage. Scale bars are 1 cm. **b** Size and diversity of Cretaceous angiosperm fruit (increasing to the left), using data from Naware and Benson^8^. Grey dots show estimated volume of fruit for fossil occurrences through time, enveloped line shows moving average of fruit size through time, with 95% confidence interval, and coloured points show the mean volume and number of lineages producing drupe-like fruit in 5-million-year time bins. **c** lineage-through-time plots (increasing to the right) of Oviraptorosauria based on *a posteriori* FBD-scaled Bayesian (blue) and parsimony (red) tree topologies, showing a drastic increase in the late Campanian, coincident with mean size increases in drupes and in fleshy fruit overall (**b**). Blue boxes show ages estimated by the morphological clock analysis for key nodes numbered in (**a**). Alb Albian, Apt Aptian, Barr Barremian, Ber Berriasian, Camp Campanian, Ceno Cenomanian, Con Coniacian, Hau Hauterivian, Maas Maastrichtian, Sant Santonian, Tur Turonian, Val Valanginian.

*Infernogallus jamesi* gen et. sp. nov. (Figs. 1–4)

#### Etymology

The name comes from the Latin “Inferno” (Hell) and “gallus” (chicken), referring to the Hell Creek Formation and the small, bird-like appearance of the species; “jamesi”, a patronym for James William Boyd, the paternal grandfather of author CAB, who sparked his interest in the field of palaeontology.

#### Holotype

ROM VP 65050, articulated partial skeleton including the skull, cervical and dorsal vertebrae, right pectoral girdle and forelimb, partial pelvic girdle, and partial right hindlimb.

#### Paratypes

ROM VP 64193, dentaries; ROM VP 64194, articular-surangular-coronoid complex; ROM VP 88690, left and right pterygoids; NDGS 4351, dentaries.

#### Locality and horizon

Hell Creek Formation (late Maastrichtian), Garfield County, Montana; Meade County, South Dakota; and Bowman County, North Dakota, USA (Supplementary Fig. 1; See “Methods”).

#### Diagnosis

Small (femur length 185 mm; est. 12 ± 3 kg body mass) caenagnathid oviraptorosaur diagnosed by the following autapomorphies: broad jugal with large anterior ascending process and which contacts the squamosal posteriorly; infratemporal fenestra extremely reduced, about 25% the diameter of the orbit; quadratojugal with greatly enlarged, circular posterior process; squamosal with dorsoventrally broad body lacking a depression and ridge for adductor musculature; pterygoids concave dorsally, unfused along the midline, and with a lateral buttress; ectopterygoid–pterygoid bar greatly expanded and extending far ventrally; long axis of vomer oriented anteroventral to posterodorsal; lateral tubercle on posteroventral ramus of dentary; extensor ridge on metacarpal II; manual phalanx I-1 less than 90% the length of II-1; tubercle proximal to condyle on metatarsal I. Differs from *Eoneophron infernalis*^44^ in its much smaller body size ( ~ 21% [range 9.4–26%] of estimated mass) at maturity, a better-developed accessory trochanteric crest of the femur, and a proximodistally shorter but more prominent cnemial crest of the tibia.

This published work and the nomenclatural acts it contains have been registered in ZooBank, the proposed online registration system for the International Code of Zoological Nomenclature (ICZN). The ZooBank LSIDs (Life Science Identifiers) can be resolved and the associated information viewed through any standard web browser by appending the LSID to the prefix “http://zoobank.org/”. The LSIDs for this publication are:

urn:lsid:zoobank.org:act:B6B53067-416C-409D-93F8-F54CB53B467F

urn:lsid:zoobank.org:act:4F3AC0D0-C842-4234-ABFD-1F3B76F22520

urn:lsid:zoobank.org:act:ADC89004-AA0F-400D-8905-7FC20BE4C17E

#### Description and comparisons

The holotype skeleton (ROM VP 65050) preserves, in articulation, the skull, cervical vertebrae, dorsal vertebrae, pectoral girdle and forelimb, and part of the pelvic girdle and hindlimb (Fig. 1). The main block was separated into four smaller blocks, resulting in the loss of part of the distal scapula and the elbow region of the forelimb. The skull of *Infernogallus jamesi* (Figs. 2, 3) superficially resembles those of oviraptorids more closely than the hypothesised reconstruction of the skull of *Anzu wyliei*^23^, but is unusual in several key features, not only among oviraptorosaurs but among theropods more broadly. The circular orbit is large, occupying nearly 40% of the basal skull length. In contrast, other fenestrae in the skull are reduced, and the infratemporal fenestra is remarkably small, 11% of the length and 12.5% of the height of the skull (Fig. 2), a unique condition among non-avian theropods. In other oviraptorosaurs, for example *Citipati osmolskae* (Fig. 2f; 29% length, 48% of height), the infratemporal fenestra is usually much larger in both length and height^18,28,45,46^. These differences between the postorbital region of *Infernogallus jamesi* and oviraptorids result in a bizarre jugal morphology for an oviraptorosaur. Whereas typically in oviraptorosaurs the jugal is a delicate rodlike bone, in *Infernogallus jamesi* it is broad, similar to those of deinonychosaurians^47^, and has a well developed lateral lip underlying the orbit. The anterior end of the jugal is bifurcated into an anterior maxillary process—typically present in oviraptorosaurs but greatly elongated in *Infernogallus jamesi*—and a tall lacrimal process that ascends nearly halfway up the orbit posterior to the lacrimal. This latter process is absent in *Anzu wyliei* and all other oviraptorosaurs except *Incisivosaurus gauthieri*^48^, where it is present but much smaller than the process in *Infernogallus jamesi*. The posterior end of the jugal is also bifurcated into a short and broad quadratojugal process and a plate-like postorbital process, contrasting strongly with the tapered postorbital processes of other oviraptorosaur jugals. In *Infernogallus jamesi*, the postorbital process further divides into two small fingers that straddle the postorbital, the posterior one contacting the squamosal (a unique contact among oviraptorosaurs^18^), and the anterodorsal one underlying much of the postorbital. The quadratojugal is also exceptional among oviraptorosaurs in its considerable size: the posterior process, which is usually tab-like in oviraptorosaurs, has expanded into a large circular plate exceeding the size of the infratemporal fenestra. Together with a large ventral process of the squamosal, it demarcates a circular external auditory meatus, contrasting strongly with the morphology in oviraptorids, where these features border a dorsoventrally extended slot.

The palate of *Infernogallus jamesi* (Figs. 2d, 4a, Supplementary Note 1 and Supplementary Fig. 2) is highly specialised compared to the already-derived palates of other oviraptorosaurs^49^. Like most oviraptorosaurs, the maxilla and vomer contact in a ventrally protruding process that would have projected into the oral cavity^18,45^. However, this is much larger in *Infernogallus jamesi* relative to oviraptorids, and the long axis of the vomer extends anteroventral to posterodorsal, instead of anteroposteriorly. The ectopterygoid-pterygoid bar, present in all oviraptorosaurs, is exceptionally large and arcs ventrally in *Infernogallus jamesi*, approaching the bottom of the external mandibular fenestra when the jaw is closed (Fig. 2c), filling most of the oral cavity. Typically, this structure extends horizontally along the bottom of the skull in oviraptorosaurs and does not extend far below the ventral edges of the premaxilla and quadrate, if at all^46^. The body of the pterygoid of *Infernogallus jamesi* has an anterodorsal prong and a transversely oriented shelf that anchored the pterygoideus musculature on its dorsal and ventral surfaces. Lateral to the shelf, the lateral surface of the pterygoid is flared dorsoventrally into a fin-like prominence, with a rugose surface that is superficially similar to the pterygoid buttress in crocodylians^50^ (Supplementary Note 2). The ectopterygoid has an unusual rod-like lateral process that bolsters the posterior end of the maxilla where it meets the jugal. The palatine is robust, and its anterior end is anteroposteriorly broad, underlying the entire anteroposterior length of the antorbital fossa, whereas it usually contacts only the posterior portion of the maxilla in oviraptorids.

The mandible is complete but crushed mediolaterally in the holotype (Fig. 2), and its internal morphology is best observed in the paratypes, particularly NDGS 4351 (Supplementary Figs. 3, 4). The completely fused dentaries are steeply upturned in lateral view, forming an edentulous beak similar to those in extant avians and turtles^16,19,51^. In *Infernogallus jamesi*, the ‘chin’ is rounded, more closely resembling mandibles referred to *Chirostenotes pergracilis* than those referred to *Leptorhynchos gaddisi* and *Elmisaurus rarus*, where it is more square^25,38,40,52^. There is a deep pneumatic fossa immediately anterior to the long, roughly elliptical external mandibular fenestra (35% the length of the mandible). Just below the pneumatic fossa on the posteroventral ramus of the dentary, there is a laterally projecting tubercle that is not present in other caenagnathid taxa (Supplementary Figs. 3, 4). The genioglossus insertion on the ventral surface of the symphysis is roughly W-shaped, rather than the heart or hourglass shape of other caenagnathids^16^. The occlusal surface of each dentary is configured like those of other caenagnathids, with a midline sulcus, a lingual ridge, and a labial groove bordered by lateral occlusal ridges (Supplementary Figs. 3, 4). Two prominent, rugose anterior occlusal ridges adorn the internal surface of the beak anterior to the midline sulcus. Like in all caenagnathids, the posterodorsal ramus of the dentary interfingers with the surangular, but the border of the surangular over the external mandibular fenestra has a shallow ventral protrusion in place of the laterally everted lip present in *Chirostenotes pergracilis*^19,52^. The articular-surangular-coronoid complex is completely fused, and the angular and prearticular are also fused into this complex, although their edges can still be discerned (Fig. 2 and Supplementary Fig. 4). The medial surface of the coronoid is rugose and opposes the buttress-like lateral surface of the pterygoid. The prearticular supports most of the ventral surface of the medial glenoid of the articular, and with the angular, these three bones form the large, hatchet shaped retroarticular process. The articular forms a rounded ridge that fits into the saddle-like cotyles of the quadrate. This ridge and saddle arrangement allows for extensive fore-aft movement of the lower jaws (Fig. 3 and Supplementary Fig. 4).

The neck of *Infernogallus jamesi* is elongate and composed of the atlas, axis, and 12 postaxial cervical vertebrae (Fig. 1 and Supplementary Fig. 5). Epipophyses, even on the anterior cervical vertebrae, are much smaller than other oviraptorosaurs^46,53^, although on the axis and first two postaxial vertebrae, they are present as small, rounded knobs. The centra and neural arches of the cervical and dorsal vertebrae are extensively pneumatized. Three transitional cervicodorsal vertebrae, which presumably had large hypapophyses based on the one exposed in the first cervicodorsal vertebra, separate the scapulacoracoid from the cervical series.

The scapula is ribbon-like and has a minimal distal expansion (Fig. 1 and Supplementary Fig. 5). The acromion is large, laterally everted as in other oviraptorosaurs^18^, and contacts a slender, boomerang shaped furcula. The pectoral glenoid faces laterally, like in *Apatoraptor pennatus* and *Elmisaurus rarus*^34,39,40^, and the coracoid has a large, rugose biceps tubercle. Only the proximal part of the humerus is preserved; it has a large deltopectoral crest, but it does not extend as far down the shaft as in some oviraptorids like *Heyuannia yanshini* and *Oksoko avarsan*^28,53^. The ulna is bowed, unlike the straight ulnae of heyuannine oviraptorids^18,46,54^. Metacarpal II is the longest in the hand and has a shallow ridge on its extensor surface that is absent in other caenagnathids. Metacarpal I is half its length. The phalanges are similar to those of *Elmisaurus rarus* and *Hagryphus giganteus*^55,56^: phalanx I-2 is shorter than phalanx II-1, and phalanx III-3 is longer than either III-1 or III-2. Ungual I-2 is larger than III-4, but like in other oviraptorosaurs, they are nearly identical in shape, with pronounced proximodorsal lips (Supplementary Fig. 6).

The ilium (Fig. 1 and Supplementary Fig. 5) is damaged, missing its posterior half, but the anterior half is much lower dorsoventrally than in other caenagnathids like *Chirostenotes pergracilis* and *Caenagnathus collinsi*^33^; it is more similar to *Elmisaurus rarus* (= *Nomingia gobiensis* sensu^40^). Like all caenagnathids, the proximal end of the pubis has a posteriorly circumscribed medial fossa. Most of the femur is preserved; it would have been ~ 185 mm long in life, much shorter than those known for *Anzu wyliei* (500–525 mm^23^). Based on the circumference of the femur, *Infernogallus jamesi* would have weighed approximately 12 kg (9–15 kg) in life^57^. There is an anterior trochanteric ridge as in many, but not all caenagnathids, including *Anzu wyliei* and *Elmisaurus rarus*^23,42^. The tibia has a large but proximally restricted cnemial crest, the anterior margin of which curves towards a ventrally directed apex, like in all oviraptorosaurs. Metatarsal I (Supplementary Fig. 6) has a large, bulbous distal condyle and is unique among caenagnathids in having a tubercle separating the condyle from the dorsal process, which is missing. The proximal part of metatarsal II provides little information, but a clean articular surface for metatarsal III tentatively suggests that the proximal metatarsus was not extensively coossified in life, contrasting with *Citipes elegans* and *Elmisaurus rarus*^38,58^. The phalanges (Supplementary Fig. 6) are long with ginglymoid condyles, and the ungual is recurved with a large flexor tubercle.

#### Osteohistology and maturity

Thin sections from the femur, fibula, and metatarsal II (Supplementary Fig. 7) exhibit cortices of fibrolamellar bone with moderately dense longitudinal to reticular vascularity. The vascularity is generally consistent in orientation throughout the cortex, but in all elements, it becomes less dense towards the periosteal surface, and the number of reticular branches decreases, making the canals appear more longitudinal in orientation. Secondary remodelling is present in the fibula and metatarsal, concentrated in regions where muscles would have attached superficially. The endosteal margins of each element show evidence of resorption and remodelling. In the femur, there are well developed endosteal lamellae, whereas in the fibula, there are multiple erosive cavities lined with lamellar bone. The metatarsal shows evidence of resorption in the form of Howship’s lacunae and cross-cut vascular canals, but there has been no redeposition of lamellar tissue in these areas. Multiple lines of arrested growth are present in the primary cortical bone of each element. Each growth mark is preceded endosteally by a thin layer of parallel-fibred bone, most apparent in the femur under cross-polarised light (Fig. 1d and Supplementary Fig. 7c, d). At the subperiosteal surface of all elements, growth marks are closely spaced in a zone of low vascularity, together comprising an external fundamental system. Growth marks suggest an age at death of at least 6 years, likely 7 based on an extra internal growth mark in the fibula. Altogether, osteohistological signals confirm skeletal maturity in this individual, despite its small size (12 ± 3 kg).

#### Implications

The remarkably complete skull of *Infernogallus jamesi* provides the first clear glimpse into the highly derived cranium of caenagnathids and their functional morphology. To date, recorded caenagnathid cranial elements have included disarticulated material from *Anzu wyliei, Apatoraptor pennatus*, *Elmisaurus rarus*, and *Epichirostenotes curriei*, and isolated bones of indeterminate species in the Dinosaur Park Formation^23,34,35,38,39^. The overall layout of the skull in *Infernogallus jamesi* (Fig. 2) differs radically from that hypothesised for *Anzu wyliei*^23^, and despite a superficial similarity in overall structure to oviraptorids, finer details of the anatomy of *Infernogallus jamesi* are unique among oviraptorosaurs and even other theropod dinosaurs (Fig. 2e, f). These peculiarities include the enlarged maxillovomeral process; the highly reduced ectopterygoid; the robust, strongly curved, and dorsally concave pterygoids; the large quadratojugal; and the small infratemporal fenestra. Nevertheless, similarities in the known elements that overlap between *Infernogallus jamesi* and other eucaenagnathines^23,34,35,38,39^, especially the quadrates and mandibles, suggest that these features were broadly shared in this group. Thus, *Infernogallus jamesi* elucidates the form and function of the eucaenagnathine skull, which has long been enigmatic.

#### Functional morphology

Prominent differences from other non-avian theropods in the mandible and temporal region of *Infernogallus jamesi* suggest a specialised function for the eucaenagnathine cranium during oral food processing. Three key characteristics of *Infernogallus jamesi* provide insight into feeding behaviour: (1) derived muscle topology; (2) mandibular joint mobility; and (3) morphology of the occlusal surfaces.

Reconstruction of individual jaw adductor muscles demonstrates that caenagnathids possessed a highly derived muscle topology compared to other non-avian theropods^26,59,60^ (Fig. 3). The anteroposterior orientation of the palatal muscles in *Infernogallus jamesi* is the most divergent feature and suggests powerful anteroposterior mandibular movements (Fig. 3b and Supplementary Fig. 8), driven primarily by the enlarged attachments for the *pterygoideus* muscles originating on the ventrally curved palatal bar. The highly increased anteroposterior orientation, and concomitantly reduced dorsoventral orientation of the large palatal muscles, suggests that these muscles could not contribute substantially to dorsoventral bite force. This muscle topology differs substantially from other non-avian theropods, where the palatal muscles are oriented more dorsoventrally and are the primary contributor to orthal jaw motion^26^ (Fig. 2e, f). Unexpectedly, reduction of the dorsoventral vector of the *pterygoideus* muscles in *Infernogallus jamesi* was not associated with a compensatory increase in the relative sizes of the other adductor muscles; instead, there is a reduction in temporal chamber volume and temporal muscle insertion area (Fig. 2). Thus, anteroposterior orientation of the enlarged palatal muscles and reduction in size of the temporal muscles indicate a substantially weaker dorsoventral bite force in caenagnathids than other oviraptorosaurs, and likely substantially weaker than other herbivorous non-avian theropods more generally^26^. Summarising muscle vectors for each adductor muscle to visualise their overall configuration demonstrates that *Infernogallus jamesi* diverged significantly in morphospace from other oviraptorosaurs and non-avian theropods (Fig. 3c), demonstrating the novelty of its muscle topology.

In eucaenagnathines, the implied anteroposterior movement of the mandible facilitated by the enlarged and reoriented *pterygoideus* muscles aligns with their distinctive ridge-and-saddle mandibular joint (Fig. 3d–f)^16,19,31^. The articular is expanded dorsally at the jaw joint into a prominent, rounded ridge, whereas the corresponding surface of the quadrate is ventrally incised with a groove, forming a smooth saddle with contralateral cotyles that straddle the articular ridge mediolaterally^19,38^. A similar arrangement is well-described in oviraptorids^28,45,54^ and earlier-branching oviraptorosaurs^48^, but in these taxa the articular ridges are weakly developed and oriented obliquely to the long-axis of the skull such that anteroposterior movement was considerably more limited than in eucaenagnathines. Based on virtual articulation of the jaw joint of *Infernogallus jamesi*, up to 15 mm of anteroposterior jaw motion was possible ( ~ 14% of skull length), conservatively assuming the bones would have remained in contact (although soft tissues likely affected true joint mobility^61,62^). In contrast, other non-avian theropods have mandibular-quadrate joints that restrict anteroposterior movement (stabilising the joint), or facilitating lateral rotation of the mandibular rami to expand the pharynx^63,64^. Interestingly, in *Infernogallus jamesi*, ventral deflection of the pterygoids into the oral cavity inherently results in a sliding interface between the medial side of the jaw and the lateral surface of the palate. This sliding of the mandible along the palate, and the resulting muscular confinement, appears to have been accommodated by a well-developed pterygoid buttress that is highly convergent with the pterygoid of crocodylians (see Supplementary Note 2). The orientation of the pterygoid buttress anteroposteriorly in *Infernogallus jamesi*, rather than mostly dorsoventrally as in crocodylians^50^, bolsters the hypothesis that anteroposterior sliding was a habitual jaw motion for eucaenagnathines.

The complex ridges and grooves on the occlusal surface of the fused dentaries have long been recognised as a distinctive caenagnathid feature^16,19,23,51^, and to our knowledge, are unique in complexity among tetrapods. However, these features take on new importance in light of the morphology of the palate revealed by *Infernogallus jamesi*. Like oviraptorids^45^, the maxilla and vomer meet in a midline ventral process, but in *Infernogallus jamesi* this process is enlarged and fits into the midline sulcus of the dentaries when the jaw is in resting position (as preserved; Supplementary Fig. 9a). During anteroposterior movement of the mandible, contact between the highly textured upper and lower jaws would have provided a mechanism for shearing, mashing, or crushing soft foods, similar to a mortar and pestle (Supplementary Fig. 9). Adjacent features of the mandible and palate support this functional interpretation; positioning of food within the mouth may have been facilitated by a powerful tongue, as attested by the prominent insertion for *m genioglossus*, a tongue protruding muscle^16,19^, a characteristic feature on the ventral surface of all caenagnathid dentaries (Supplementary Figs. 3, 4). Likewise, the ceratobranchials are relatively long in caenagnathids^34^, which is linked to enhanced tongue mobility in birds^65^.

Altogether, the unusual combination of anteroposteriorly oriented jaw muscles; exaggerated ridge-and-saddle jaw joint; well-developed, anteroposteriorly oriented pterygoid buttress; complex occlusal surfaces; and large tongue musculature in *Infernogallus jamesi* all demonstrate that this species, and likely caenagnathids more generally, were highly adapted for specialised oral food processing. Based on the orientations of the jaw musculature, in tandem with increased quadrate-articular mobility, we hypothesise that a typical chewing stroke would have included substantial anteroposterior movement of the jaw (Supplementary Note 3 and Supplementary Fig. 9), facilitating occlusion of the dentaries and palate with relatively low forces. Other herbivorous non-avian theropods lack these adaptations and are generally understood to have relied on high-fibre plant matter^24,66–68^, or possibly durable materials like nuts, seeds, and shoots^26^. High-fibre herbivory is usually facilitated in these other herbivorous taxa either with teeth or a gastric mill, both present in ancestral oviraptorosaurs^69^ but lost in avimimids, caenagnathids, and oviraptorids, suggesting high-fibre herbivory is unlikely in caenagnathids. Indeed, despite the articulated nature of ROM VP 65050 (including a complete gastral basket), no potential gastroliths were found in the body cavity during preparation, suggesting their absence in caenagnathids. Likewise, durophagy is typically associated with adaptations to facilitate high bite force, like stable jaw joints and flat, crushing occlusal surfaces^70^. These adaptations for durophagy are observed to a greater degree in oviraptorids, but are totally absent or reversed in caenagnathids. In sum, caenagnathids lacked adaptations for processing fibrous or durable vegetation that are present in other herbivorous non-avian theropods, and would therefore have been restricted to a diet of soft, non-fibrous foods, which nevertheless apparently required extensive manipulation and processing.

#### Extant analogues

We evaluated potential extant analogues for the anatomy and potential dietary function of *Infernogallus jamesi*, focusing on the two edentulous beaked clades (Testudines and Aves). No single extant analogue possesses every craniomandibular adaptation present in *Infernogallus jamesi*, but each of the three key features noted above is converged upon by at least one of these extant groups. Critically, the most closely analogous species within these groups are always herbivores, and in particular, those which extensively orally process soft foods (folivorous and frugivorous parrots, and herbivorous sea turtles [Supplementary Fig. 10]). Our analyses (see “Methods”) indicate that psittaciforms are the most compelling overall analogue for the cranial adaptations we find in caenagnathids, and they therefore form the focus of the comparison here (see Supplementary Note 5).

Superficial resemblance between oviraptorosaur and psittaciform skull shape has long been appreciated, but it has remained less clear whether these gross anatomical similarities indicate shared function. Accordingly, to contextualise function as reflected by muscle orientations, we reconstructed muscle vectors for a wide range (*n* = 19) of theropod dinosaurs and birds with varied diets, focusing on varying modes of herbivory and building on the most comprehensive myological datasets available (see “Methods”)^59,60^. As expected, most extant birds have enhanced anteroposterior jaw musculature when compared to other theropods (Fig. 3c), almost certainly reflecting constraints imposed by large eyes and brains^60,71–73^. Most non-avian theropods retain high dorsoventral components, whereas *Infernogallus* is distant in morphospace from other non-avian theropods due to its reduction of the dorsoventral musculature and the increased anteroposterior component (Fig. 3). In contrast to birds, it is unlikely that the increased anteroposterior component of muscle vectors in *Infernogallus* results from increased encephalization or eye size, because this pattern in this taxon is primarily driven by rearrangement of the palate (specifically the pterygoid), and the braincase is small, not encroaching into the temporal region. Likewise, the orbit of *Infernogallus*, although large, is not markedly expanded relative to other oviraptorosaurs that have more dorsoventral adductor musculature orientation—instead, the temporal region is reduced in size. However, a suite of birds whose diet is predominantly soft plant parts (either folivorous or frugivorous) show similar reductions in muscular dorsoventral vector components. Of these birds, those with the most extreme reduction of dorsoventral components are *Psittrichas fulgidus*, an obligately frugivorous psittaciform, and *Struthio camelus*, an omnivore. Detailed comparison of muscle component vectors in *S. camelus* shows that its position in morphospace is driven by a uniform anteroposterior shift in all jaw muscles, likely a result of encephalization rather than functional specialisation of the musculature^60^. Furthermore, it lacks a strong ridge-and-saddle joint to facilitate anteroposterior sliding of the mandible. In contrast to *Struthio*, the component vectors in *Psittrichas fuldigus* are remarkably similar to *Infernogallus* in spanning a wide range of orientations, and include well-developed musculature that facilitates anteroposterior movement of the lower jaws. Psittaciforms in general have the most advanced oral processing capabilities among birds, but unlike most psittaciforms, *Psittrichas fuldigus* processes fruit by consuming the pulp and discarding the seed. In contrast, most other parrots like *Psittacus* (Psittacidae) are granivorous and target the internal seed by employing strong cracking motions of the lower jaw to husk or shell the seed and consume the nutmeat—behaviours facilitated by their retention of strong dorsoventral vector components (Fig. 3). Gross anatomical comparisons of *Psittrichas fulgidus* with other psittaciforms reveals several specialisations that parallel the unique cranial anatomy of *Infernogallus jamesi* relative to other oviraptorosaurs—both taxa are distinctive among their respective clades^74,75^. *Psittrichas* has a longer, lower cranium and mandible (generally associated with softer diets in psittaciforms^76^), enhanced articular ridges of the mandible, and exhibits lower dorsoventral components and higher anteroposterior components of the adductor musculature (Fig. 3c, f). Considering the skulls of ancestral oviraptorosaurs and psittaciforms generally converged on the same boxy shape, we interpret the parallel trajectories towards longer, lower mandibles and associated myological adaptations in *Infernogallus* and *Psittrichas* as evidence for similar dietary pressures operating on the cranium.

Comparison to extant analogues highlights the sophistication of the food processing capabilities in caenagnathids, as *Infernogallus* possesses multiple adaptations that each confer a high degree of food processing ability in living animals. In reducing bite force and reorienting muscle vectors, these traits are incompatible with processing fibrous or durable foods and are instead most consistent with adaptations for processing soft materials. However, the question remains as to which foods are sufficiently soft, yet require extensive mechanical processing. Based on obligate pulp-mashing frugivory in the closest extant analogue we could identify (*Psittrichas fulgidus*), we hypothesise that *Infernogallus jamesi* could have been specialised for processing soft fleshy fruit, although it is likely that other soft plant materials also formed a significant component of its diet. Therefore, we next consider whether a partially frugivorous diet is consistent with the fossil records of caenagnathid and fruiting angiosperms.

#### Caenagnathid evolution

Improved resolution of caenagnathid relationships and origination times based on a comprehensively revised phylogenetic matrix show that a diet incorporating a high proportion of fruit is reasonable for this group (Fig. 4, Supplementary Note 4, Supplementary Figs. 11, 12). Bayesian tip-dating and *a posteriori* fossilised birth-death (FBD) models (Fig. 4) both unambiguously estimate the origin of caenagnathids in the Aptian–Albian, shortly after the origin of angiosperm fruits^7,8^, including drupes (fruits with a single large internal seed surrounded by a well-developed fleshy mesocarp). Reanalysis of the earliest caenagnathid, *Microvenator celer*, reveals that it incipiently exhibited the features of the pterygoids and dentaries that are inferred to have enabled complex oral food processing in *Infernogallus jamesi* (Fig. 4 and Supplementary Note 7), and relaxed clock models indicate that caenagnathids had elevated rates of mandibular evolution (Supplementary Fig. 12b). This indicates that potentially frugivorous adaptations arose early and rapidly in the caenagnathid lineage. FBD and relaxed clock models predict a late Santonian–early Campanian origin for Eucaenagnathinae, the clade that shows the most advanced craniomandibular adaptations, which is congruent with the estimated size increase and diversification dates of major fruiting angiosperm lineages^7,8,77,78^ (Fig. 4b). Caenagnathids undergo a major diversification and range expansion from the Campanian onwards, perhaps associated with a proliferation of new herbivorous niches as angiosperms diversified during this interval. Fleshy fruit originate in multiple angiosperm lineages and increase in size^8,77–84^, and in particular, drupes become larger, more abundant, and more diverse from the Coniacian onwards^7,85^ (Fig. 4b). The eucaenagnathine oral apparatus has adaptations consistent with of defleshing such fruits, a typical behaviour in extant *Psittrichas fulgidus*. Thus, the apparent lock-step timing of adaptations arising in caenagnathids and diversification and size increase in fruiting angiosperms are consistent with the hypothesis that oral processing of fruits formed a significant component of caenagnathid diets (and see Supplementary Note 6). Critically, previous studies of broad-scale correspondence between dinosaur and angiosperm diversity have shown poor synchronicity^6,10–12^, suggesting that the pattern observed here between caenagnathid and fruit evolution is not a simple consequence of broader evolutionary dynamics. Altogether, the congruence of biomechanical adaptations, functional convergences, and the timing of these anatomical changes supports the idea that caenagnathids were adapted for at least partially frugivorous diets (but see Supplementary Note 8). This is consistent with recent analyses showing earlier, Cretaceous origins of large angiosperm diaspores, particularly fleshy fruit, in southern North America (where caenagnathids were present^86–88^), indicating that angiosperm-animal interactions were probably more extensive than previously recognised^7^.

Further testing the hypothesis of frugivory in caenagnathids could be carried out with additional fossil evidence (e.g., stomach contents) or more complex biomechanical modelling to further elucidate the chewing mechanics of caenagnathids, as well as the development of better proxies for modes of frugivory based on living species. To this last point, frugivores are often lumped as a single catch-all category in dietary studies, despite a wide range of functional behaviours. Indeed, many frugivorous birds do not orally process food at all, consuming fruit whole, whereas others have complex behaviours discarding the flesh to consume the seed (and might better be called granivores), and others still consume the flesh, discarding the seed. In tandem with the multifunctional nature of the cranium and beak^73,74,89^, this makes it difficult to generalise a single functional signal of frugivory in birds. The challenge of projecting a dietary signal to theropods is further complicated by the extensive modification of the skull in birds as compared to their ancestors^60^, confounding statistical attempts to test for function (see Supplementary Note 9 and Supplementary Fig. 13). For these reasons, we argue that detailed comparative anatomy and functional morphology are more appropriate in this case than a broad-scale statistical approach, until larger, more detailed avian dietary behaviour datasets are available alongside muscle vectors. Based on our qualitative observations and comparisons, we hypothesise that, if caenagnathids did indeed consume fruit, a defleshing habit, similar to *Psittrichas*, is the most likely mode of consumption, reflecting the complexity of the caenagnathid oral processing apparatus and their low bite forces.

The discovery of *Infernogallus jamesi* from the latest Cretaceous is also intriguing with regard to our understanding of Late Cretaceous diversity and survival dynamics of the end-Cretaceous extinction, when non-avian theropods like caenagnathids went extinct. The discovery of a small-bodied, highly specialised taxon in a well-studied system like the Hell Creek Formation highlights that not only is taxonomic diversity affected by taphonomic size biases^36^, but that measures of functional disparity may be undersampled as well. The combination of small body size ( ~ 12 ± 3 kg; below the hypothesised extinction body-mass threshold^90,91^), presumably avian-like metabolism, and herbivorous diet of *Infernogallus jamesi* overlaps considerably with the features proposed to have enabled birds to survive the end-Cretaceous extinction^92^. However, our analyses suggest that it had a highly specialised diet, which may have consisted in large part of sporadic or seasonal foods (fruit flesh) rather than persistent materials (the seeds within). Previous work suggests that the capability to access persistent food sources was likely a key factor impacting survival when primary resources underlying terrestrial food webs collapsed at the end of the Cretaceous^92^. Thus, despite the proliferation of angiosperms and their fruit in the early Cenozoic, the oviraptorosaur radiation, like other non-avian dinosaurs, went extinct.

## Methods

### Specimens

All five specimens are from the Maastrichtian Hell Creek Formation (see detailed locality information below). All specimens were collected in accordance with local permitting requirements and are permanently accessioned at accredited museums where they are available for study in perpetuity. The holotype and paratype were discovered independently by two different research groups working in separate field areas of the Hell Creek Formation. The decision was made to combine the efforts of the two to help ensure the long-term stability of this new taxon and facilitate future referrals of new specimens. All specimens were incorporated into the description here, which represents a full but brief account of the known morphology of this taxon. A manuscript focused on a detailed description of the paratype, which was in progress before the efforts were combined, will follow the present study.

The holotype material (ROM VP 65050) was collected in Garfield County, Montana, USA, by C. Phipps in July 2011 with appropriate permissions from the private, deeded land owner. Detailed location information is on file at the Royal Ontario Museum. This land is the traditional territory and home of the Niitsítapi; Očhéthi Šakówiŋ; Apsáalooke; Tséstho’e; Cayuse, Umatilla, and Walla Walla; and Métis peoples, who have been stewards of these areas since time immemorial. Initial preparation was conducted by CK Preparations (Montana) prior to acquisition by the ROM in April 2012, but no reconstructive work was done, and final preparation was carried out by ROM staff (S. Sugimoto, I. Morrison). CT scans confirm the authenticity of the specimen, including the skull.

### Detailed locality information

#### ROM VP 65050

The holotype material was collected from private land in the northeastern part of Garfield County, Montana, USA, by C. Phipps in July 2011. The specimen was preserved in a concretion within an organic-rich, medium-grained sandstone approximately 3.75 m thick (Supplementary Fig. 1). The site is 15.5 m below the K/Pg boundary (measured with a Jacob’s staff by DCE), which is approximated by the base of a 4 m-thick coal swarm exposed on top of a ridge ~ 1 km to the NE. The specimen was acquired by the ROM in April, 2012 with a ROM Strategic Acquisition Grant to DCE from the Louise Hawley Stone Charitable Trust. Detailed location information, including exact GPS coordinates, is on file at the Royal Ontario Museum, as is a letter from the BLM stating its provenance from private, deeded land.

#### ROM VP 64193 and 64194

These two specimens, comprising partial dentaries and an articular-surangular-coronoid complex, were collected together from private land the south-central part of Garfield County, Montana, USA. The specimens were collected from the same channel complex that produced the holotype of *Acheroraptor temertyorum*^93^. The specimens are identical in preservation style and correspond to an animal of the same size, and therefore, we interpret them to belong to the same individual. Detailed location information is on file at the Royal Ontario Museum.

#### ROM VP 88690

ROM VP 88690 was acquired by the ROM in 2015. It was collected from the Hell Creek Formation exposed near Faith, South Dakota. Detailed location and provenance information is on file at the Royal Ontario Museum.

#### NDGS 4351

NDGS 4351 was collected in 2019 by one of the authors [DF] from locality NDGS L10006 ( = PTRM V87012^94^) in the Mud Buttes area of southwestern Bowman County, North Dakota on lands managed by the Bureau of Land Management. It was collected under BLM permit MTM 108845 during a field trip for the Cretaceous and Beyond symposium held in Dickinson, North Dakota in 2019. Further details are available to researchers upon request. The specimen was collected from a microvertebrate accumulation preserved within a silty mudstone interpreted as a floodplain environment within the upper portion of the Hell Creek Formation, 9.85 metres below the Cretaceous-Paleogene boundary^94^ (Pearson et al.^94^), Lancian North American Land Mammal Age, within plant zone HCIII^95^.

### Data collection

Palaeohistological samples were taken from the femur, fibula, and metatarsal II of ROM VP 65050. No separate permits or permission were required for destructive sampling. Fragments of bone were removed from the areas closest to the midshaft, embedded in Buehler Epothin II epoxy under vacuum, and sectioned on an Isomet 1000 Precision Saw. After being mounted to plexiglass slides, samples were resectioned on the Isomet 1000 Precision saw and ground to thickness on a Hillquist Sectioning Machine, before being polished using a series of 600-, and 1000-grit silicon carbide abrasive, followed by polishing with Al-O_2_ powder. Thin sections were imaged on a Nikon AZ100 microscope using NIF Elements software. Palaeohistological terminology follows Padian and Lamm (2013).

The skull block of the specimen was µCT scanned using a Precision X-Ray SmART+ system at the STTARR facility of the University Health Network in Toronto, ON, Canada. Scans were taken at a pixel size and slice distance of 100 µm, with X-rays generated at 80 V and 3 mA. Because the field of view was just smaller than the skull, three sets of images were taken, of the anteroventral, anterodorsal, and posterior parts of the skull. Segmentation of the µCT dataset was done in Slicer 4.10.2, and segmentations from the three skull regions were aligned in Meshlab v2020.06 using the ‘Align’ toolset. Fragmentation of the bones and the presence of high-density, iron-rich sediment grains in the matrix surrounding the skull made automatic segmentation impossible and discerning bone surfaces in the inside of the skull difficult, especially around the braincase. In other areas, this resulted in floating ‘islands’ of bone. As a result, in some cases the µCT segmentations do not fully capture the entire internal structures of the bones preserved, although they do accurately reflect the external morphologies of these elements. For some intimately associated elements like the braincase bones, sutures between the bones could not be discerned from the µCT data, and so these were segmented as single elements. Segmentations of the bones were retrodeformed in Blender (v3.1.2) to correct for post-mortem crushing, using undistorted specimens from other caenagnathids and oviraptorids as guides (see below).

Surface scans of NDGS 4351 were conducted using an EinScan-SP 3D scanner using the EinScan software. A fixed scan was conducted using the built-in turn table with scan data collected every two degrees. NDGS 4351 was scanned in four different orientations to ensure complete coverage of all surfaces of the specimen. A watertight model was then exported using the high detail setting, resulting in a model consisting of 10.05 million polygons. These scans allowed areas of the specimen to be imaged in orientations that would not be possible with standard photography methods (e.g., medial surfaces). Copies of this model are available upon request from the North Dakota Geological Survey.

All statistical analyses and data visualisation was done in RStudio using the ggplot2 package^96,97^, alongside other task-specific packages outlined below. Figures were compiled in Adobe Photoshop 2026.

### Muscle vector analysis

Whereas muscular insertions could be readily identified on the surangular of ROM VP 64193 and the pterygoids of ROM VP 88690, crushing of the internal parts of the skull and the inability to directly observe the bone surface within the adductor chamber mean that muscle origins could not be identified directly in ROM 65050. However, the presence of these muscle groups is deeply conserved within Archosauria, and positions for muscular origins were recently reconstructed for closely related oviraptorids by Meade and Ma^26^. Therefore, we used the corresponding muscle origin sites described by Meade and Ma^26^ to reconstruct muscular origins and found no incompatibilities between the anatomy of *Infernogallus jamesi* and these inferred origins. Using the observed muscle insertions and hypothesised muscle origins, we traced vectors from centroid to centroid to infer lines of action for the adductor musculature in three dimensions. These were done in three positions: one with the mandible at the anterior extent of its articulation with the quadrate, one in the middle, and one with the mandible at the posterior extent of this articulation (the position in which it is preserved). We did not scale muscle vectors to force, and without more information about the origin sites and associated musculature, it is not possible at this time to estimate maximum gape and account for this in the lines of action. Accordingly, we used a 5° gape when calculating muscle vectors (see below), as in Cost et al.^59^, and we used a conservative 23° gape when reconstructing the open position of the mandible during chewing, which is near the optimal gape for the range of oviraptorosaurs reconstructed by Meade and Ma^26^.

### Retrodeformation

The temporal arches and lower jaw of ROM 65050 were retrodeformed in Blender (v3.1.2) to approximate their proportions and positions in life, following the methods of Lautenschlager ^98^. A detailed description of our methods follows. The specimen is compressed mediolaterally, with little of the right side remaining intact, but the left cranial elements are semi-articulated and could be reconstructed with little modification. Fractured and displaced segments of the jaw were reconnected to form a single element, which was then plastically deformed mediolaterally using a lattice modifier to follow the shape of an undistorted jaw of the eucaenagnathine *Chirostenotes pergracilis* (TMP 2001.012.0012). For the temporal arch, the postorbital, squamosal, quadratojugal, and jugal were translated laterally and rotated such that the articular surfaces of each bone were contacting their neighbour. A lattice modifier was then applied to plastically deform the braincase mediolaterally to follow the proportions of other well-preserved oviraptorosaur cranial material (UWBM 120330; *Conchoraptor gracilis*, MPC-D NatGeo.2018.036a; *Oksoko avarsan*, MPC-D 102/11; *Nemegtomaia barboldi*, MPC-D 100/2112).

### PCA and Ternary plots

To compare muscle orientations between *Infernogallus jamesi* and other dinosaurs, we decomposed muscle orientations into their constituent orthogonal vectors. We visualised these in two ways. First, we visualised them in ternary diagrams to show individual muscle vectors for each taxon. Coordinates for the approximate centroid of the origin and insertion sites for each muscle were recorded in Blender for *Infernogallus jamesi*, and comparison taxa: the carnivorous tyrannosaur *Tyrannosaurus rex* (data from Cost et al.^59^); the herbivorous therizinosaur *Erlikosaurus andrewsi* (MPC-D 100/111^99–101^); the early-diverging oviraptorosaur *Incisivosaurus gautheri* (IVPP V13326; Meade and Ma^26^); the oviraptorids *Citipati osmolskae* (MPC-D 100/798; Meade and Ma^26^), *Conchoraptor gracilis* (MPC-D 100/3006; Meade and Ma^26^), and *Khaan mckennai* (MPC-D 100/973; Meade and Ma^26^); the extant parrots *Psittacus erithacus* (MUVCAV042; Cost et al.^59^) and *Psittrichas fulgidus* (YPM ORN 141821; https://www.morphosource.org/concern/biological_specimens/000S29980); and the avians *Struthio camelus* (Data from Cost et al.^59^), *Gallus gallus* (Data from Cost et al.^59^), *Buteo rufofuscus* (NHMUK 1862.1.18.1; https://data.nhm.ac.uk/object/bb8fe44f-723e-4c5f-b5a7-82208ede3bd8/1737590400000), and *Geospiza fuliginosa* (NHMUK A1975.15.25; https://www.morphosource.org/concern/biological_specimens/000S29995). These taxa were chosen because they represent a range of diets, have 3-dimensional models already available, and have detailed dissections of their mandibular musculature published. For each extant taxon, muscular attachment sites and vectors were cross-referenced with available dissections. The origin and insertion for mPSTp was not reconstructed for *Infernogallus jamesi* because the hypothesised site of origin is not preserved enough for an accurate estimate. In addition, we supplemented our dataset with additional avian and dinosaurian taxa from Wilken et al.^60^. Coordinates for each muscle were converted into relative rostrocaudal, mediolateral, and dorsoventral components and plotted using the R package MuscleTernary (Cost et al.^59^).

For the second visualisation, we summarised the overall organisation as reflected in the mean value of anteroposterior, dorsoventral, and mediolateral components of musculature for each taxon. This reduced the dimensionality of the data while maintaining overall relationships between anteroposterior and dorsoventral muscle vector components, and allowed us to incorporate data with varying numbers and identities of muscles. These data were visualised using principal components analysis to reconstruct a morphospace of mandibular adductor organisation, similar to a geometric morphometric approach, but using functional data directly instead of discerning functional signals from shape data.

### Phylogenetic analysis

The phylogenetic matrix of Funston et al. (2020) was comprehensively updated to provide a new, easier-to-use foundational matrix for analysis of Oviraptorosauria. In addition to introducing new characters, revising and outlining character states in more detail, and deleting some poorly constructed or redundant characters, the character list was re-ordered so that characters are grouped by anatomical regions. Several taxa were excluded based on inappropriateness for the analysis. These include *Banji long* and *Beibeilong sinensis* (known only from young juvenile material), *Ningyuansaurus wangi* (likely not an oviraptorosaur), and *Leptorhynchos gaddisi*, *Luoyanggia liudanensis*, and *Ojoraptorsaurus boerei* (too fragmentary to be informative or of dubious validity). The resulting matrix had 42 taxa scored for 271 characters. All matrices and scripts for analysis are provided as Supplemental Data Files. Three phylogenetic approaches were used on this matrix.

The first phylogenetic approach used TNT v1.5 to conduct a conventional maximum parsimony analysis, with 10,000 replicates of tree-bisection-reconnection, followed by a subsequent round of branch breaking to find additional most parsimonious trees. Bremer support values were calculated using the bremer.run script provided with TNT. To assess divergence dates of oviraptorosaur clades, we used an *a posteriori* fossilised birth-death model implemented in the paleotree package in R. We used the mean speciation rate (λ = 0.008684), extinction rate (µ = 0.950842), and sampling rate (s = 0.116264) estimated by a morphological clock Bayesian analysis (see below). We sampled 100 trees and calculated the mean age for each node of each tree, to produce a single *a posteriori* time-calibrated tree.

For the second approach, we used a Bayesian optimality criterion with the full dataset, without any time calibration, to evaluate whether Bayesian topologies differed substantially from parsimony results. We partitioned the characters into five anatomical regions: the skull, mandible, axial column, forelimb, and hindlimb, and allowed rates between partitions to vary. We used the MK(v) prior for character transformations and ordered 5 characters: 1, 8, 10, 103, and 263 (see Supplementary Data Files). We constrained the tree topology to include a monophyletic Oviraptorosauria, Caenagnathoidea, Caenagnathidae, and Oviraptoridae, based on uncontroversial membership in recent parsimony-based trees, which were also recovered during preliminary unconstrained Bayesian analyses. We ran the analysis using the CIPRES Gateway^102^ for 120 M generations or until convergence, which typically happened after about 10 M generations. Samples were taken every 1000 generations, and the first 25% of samples was discarded as burn-in. From the tree sample, we chose the single tree with the maximum posterior probability.

The third phylogenetic analysis refined our Bayesian approach with a relaxed morphological clock analysis, to our knowledge, the first for Oviraptorosauria. Analyses with the full matrix failed to converge in a reasonable number of generations because of the prevalence of missing data in the dataset. Therefore, we trimmed the dataset to include 29 taxa that were either scored for a substantial number of characters or occupied key stratigraphic intervals to assist in anchoring nodes. We used the skyline fossilised birth–death process with either three or four rate partitions to tip-date the phylogeny. In the three-rate analysis, we set breaks at 126 ma and 66 ma, reflecting the first appearance of oviraptorosaurs and their extinction, because the matrix focuses on this clade and has inconsistent sampling outside of this interval. In the four-rate analysis, we likewise set breaks at 126 ma and 66 ma, but added a break at 83.6 ma, reflecting the much better fossil record of oviraptorosaurs from the Campanian until the end of the Maastrichtian. We used the sampling probability for theropods from Starrfelt and Liow^103^. Taxon ages were sampled from a uniform distribution of stratigraphic occurrences, and a dummy extant taxon constrained to *Anzu wyliei* (with *Infernogallus jamesi* and *Eoneophron infernalis*, the youngest taxon in the dataset) was used to anchor the trees. The root age of the tree was sampled from an offset exponential distribution between 228 and 235 ma, the age range of *Herrerasaurus ischigualastensis*. We used the TK02 best-fit autocorrelated relaxed clock model, modelled with random sampling but corrected for sampling exclusively fossil taxa, and calculated the clock rate prior from a non-clock analysis of the same matrix (without the dummy) under otherwise identical conditions, following Gunnell et al. (2018). Analyses were run using the CIPRES Gateway^102^ for 120 M generations or until convergence, indicated by the average standard deviation of split frequencies dropping below 0.01. Each focal analysis converged before reaching the set number of generations, at roughly 9 M generations in the three-rate analysis and 41 M generations in the four-rate analysis. Samples were taken every 1000 generations, and the first 25% of samples was discarded as burn-in. Convergence was assessed in Tracer v1.7.2, and trees were plotted in Figtree and in R using the ggtree package^104^.

To evaluate angiosperm fruit diversity dynamics, we used the dataset from Naware and Benson^8^, which we plotted using commands from ggplot2. From this dataset, we summarised data pertaining to drupe size and taxonomic diversity.

### Reporting summary

Further information on research design is available in the Nature Portfolio Reporting Summary linked to this article.

## Supplementary information

Supplementary Information

Description of Additional Supplementary File

Supplementary Data 1

Reporting Summary

Transparent Peer Review file

## Data Availability

Fossil specimens in this study are permanently deposited at the Royal Ontario Museum (ROM VP 65050, ROM VP 64193, ROM VP 64194, and ROM VP 88690) and the North Dakota Geological Survey (NDGS 4351), where they can be accessed by contacting the relevant curator. Data used to conduct this study (including all Source files for figures) are available as Supplementary Data files (Supplementary Data 1). The µCT, phylogenetic and quantitative data generated in this study, including all data and code used to generate the figures, have been deposited in Figshare^105^ (https://doi.org/10.6084/m9.figshare.31901296).

## Code availability

Phylogenetic analysis scripts are available in Figshare^105^ (https://doi.org/10.6084/m9.figshare.31901296). Four directories and a ReadMe file are provided in the.zip archive. No custom code or software was used in the study.
