# Supplementary material for: Fruit-eating adaptations in a Late Cretaceous oviraptorosaur (Theropoda)
Source: Nat Commun. 2026 Oct 10;17:11549. doi: 10.1038/s41467-026-78343-w (PMC13748659; doi:10.1038/s41467-026-78343-w)
Supplement: Supplementary file 1 [file 41467_2026_78343_MOESM1_ESM.pdf]

Supplementary Materials for:
Fruit-eating adaptations in a Late Cretaceous oviraptorosaur (Theropoda)

Authors: Gregory F. Funston[1,2,3*], Thomas W. Dudgeon[2,4], D. Jade Simon[2,4,], Danielle M. Dufault[2], Clint Boyd[5], Denver Fowler[6], Philip J. Currie[7], David C. Evans[2,4*]

Affiliations:
[1] Stony Brook University, Department of Anatomical Sciences, Stony Brook, NY, United States of America
[2] Royal Ontario Museum, Department of Natural History, Toronto, ON, Canada
[3] University of California, Davis, Department of Earth and Planetary Sciences, Davis, CA, United States of America
[4] University of Toronto, Department of Ecology and Evolutionary Biology, Toronto, ON, Canada
[5] Fossil Resource Management Program, North Dakota Geological Survey, Bismarck, ND, United States of America
[6] Badlands Dinosaur Museum, Dickinson Museum Center, Dickinson, ND, United States of America
[7] University of Alberta, Department of Biological Sciences, Edmonton, AB, Canada

*Correspondence should be addressed to:
gregory.funston@stonybrook.edu ; davide@rom.on.ca

Contents:

Supplementary Notes:
- Supplementary Note 1: Expanded description of the palate of *Infernogallus jamesi*
- Supplementary Note 2: Pterygoid buttresses in crocodylians and caenagnathids
- Supplementary Note 3: Reconstructing the chewing stroke of *Infernogallus jamesi*
- Supplementary Note 4: Phylogenetic results
- Supplementary Note 5: Additional comparison to extant analogues
- Supplementary Note 6: Additional evidence of angiosperm-based diets in caenagnathids
- Supplementary Note 7: Palatal evolution in Oviraptorosauria
- Supplementary Note 8: Diet in other oviraptorosaurs
- Supplementary Note 9: Notes on statistical approaches

Supplementary Table 1. Measurements of ROM VP 65050

Supplementary Figs. 1–13

References

Supplementary Note 1: Expanded description of the palate of *Infernogallus jamesi*

The palate of *Infernogallus jamesi* is known from two specimens (ROM VP 65050 and ROM VP 88690), which together provide detailed anatomical information for each palatal bone. The palatal complex of *Infernogallus jamesi* consists of the pterygoid, ectopterygoid, palatine, vomer, and a portion of the maxilla.

Pterygoid—The pterygoid is the largest and most robust bone of the palate. It consists of two main components, the posterior wing and the anterior shelf, connected by a curved, circular ramus. The posterior wing is roughly triangular in lateral view, and is mediolaterally compressed into a relatively thin sheet of bone. Along the dorsal surface of the pterygoid wing, it is pierced by a deep, ovoid pneumatopore. Ventral to this, the posterior two thirds of the medial surface of the wing is inset to accommodate the basipterygoid process of the basisphenoid. The ventral margin of this contact forms a slot into the bone, bordered by a thin medial flange that would have wrapped around the ventral edge of the basipterygoid process, forming a tongue-and-groove joint. This kind of 'tongue-and-groove' or 'chevron-shaped' contact is often associated with mitigating compressive stresses [1]. Anterior to the basipterygoid contact on the medial surface of the wing, there is a platform of raised bone with a distinctive circular knob, which may have braced the base of the parabasisphenoid rostrum. The lateral surface of the pterygoid wing is gently faceted where it would have contacted the optic wing of the quadrate. The border of this contact is marked by a slight ridge, which extends posteroventrally to anterodorsally. Based on these contacts, the wing of the pterygoid would have slotted tightly in between the optic wing of the quadrate and the basipterygoid process, but it seems not to have fused to the basipterygoid process as occurs in some theropods.

The ramus of the pterygoid is thickened and roughly circular in cross-section, but with a flattened medial face. It extends anteroventrally from the pterygoid wing at an angle of about 45° from horizontal. The lateral surface of the ramus has a sharp ridge that extends from the ventral edge of the pterygoid wing towards the lateral surface of the anterior shelf, eventually bifurcating anteriorly to form the dorsal and ventral edges of the lateral pterygoid buttress.

The anterior part of the pterygoid is complex, comprising a dorsally- and ventrally-concave, transversely-oriented shelf, bordered medially and laterally by vertically oriented areas of bone. The medial part of the anterior end is sheet-like, with a flattened medial surface that seems to have contacted its counterpart on the opposing pterygoid, along the midline. In ROM VP 88690, this region is dorsally deflected and tapers anteriorly, producing an elongate, triangular splint of bone. The ventral edge of this splint is broken, and it may have extended to meet the transverse shelf, forming a continuous wall. However, the same triangular splint is found in the µCT segmentations of ROM VP 65050, which may suggest that it reflects the shape in life. In other oviraptorosaurs[2–4], the corresponding medial portion of the anterior pterygoid extends anteriorly to meet the vomer and part of the palatine, but there is no evidence for these contacts on the triangular splint of bone in *Infernogallus jamesi*. Instead, a small notch in the anterior margin of the transverse shelf in ROM VP 88690 probably corresponds to the foramen that typically pierces the palate posterior to the choanae and which separates the contact between the palatine and vomer from the contacts of each of these bones with the pterygoid. This is supported by the µCT data from ROM VP 65050, which further suggest that there is no contact between the pterygoid and vomer in *Infernogallus jamesi*, and that the vomer is shortened, rotated, and contacts only the palatine and maxilla (see below).

The transverse shelf of the pterygoid has concave upper and lower surfaces which face directly dorsal and ventromedial, respectively. The dorsal surface is marked by a prominent ridge that separates the ramus from a depression that is continuous with the lateral surface of the medial process of the pterygoid. This ridge, and the corresponding posterior edge of the depression, extends posteromedially to anterolaterally, with the anterior end of the ridge reaching the lateral edge of the transverse shelf. The depression anterior to the ridge has a slightly hummocky surface that grades into a more porous surface anteriorly. Based on muscular insertions reconstructed for birds, crocodylians, and other theropod dinosaurs [5], we interpret the depression as the origin of *m pterygoideus dorsalis*. The ventral surface of the transverse shelf is inclined, so that it reaches further laterally than it does medially, extending between the ventral edge of the lateral buttress and the ventral margin of the medial process of the pterygoid. At the posterior end of the shelf, the ventral surface has a subtle groove extending posteromedially to anterolaterally, associated with several smaller anteroposteriorly-oriented ridges. This groove, and a surface widening posteriorly and tapering anteromedially, is rugose, with a fibrous appearance. Lateral to this rugose patch, the ventral surface is striated by anteroposterior grooves. We interpret the entire ventral surface of the transverse shelf as anchoring *m pterygoideus ventralis*, the posterior border of which is marked by the subtle transverse groove.

The lateral surface of the transverse shelf is broadened dorsoventrally into an almond-shaped pad, strikingly similar in appearance to the pterygoid buttress in extant crocodylians [6]. We therefore refer to it as the pterygoid buttress (see below). The pterygoid buttress in *Infernogallus jamesi* is taller posteriorly, becoming dorsoventrally shorter anteriorly. Its dorsal edge gently slopes ventrally towards the anterior end, whereas the ventral edge is steeply sloped at its posterior end, but then flat anteriorly. At the posterior end, the buttress is confluent with the bifurcating ridge of the ramus, contributing to its almond-like shape. There is a small, smooth depression on the posteroventral part of the buttress in lateral view, which is roughly circular. Otherwise, the lateral surface of the buttress is rugose and gnarled, identical to the texture in crocodylians when the overlying soft tissue is removed (e.g., ROM R9549 *Caiman crocodilius*).

Ectopterygoid—The ectopterygoid is preserved in ROM VP 65050, where only a small portion is visible externally, but µCT data reveal the remainder of the bone. Like the ectopterygoid of most oviraptorosaurs, the ectopterygoid of *Infernogallus jamesi* is simplified compared to most theropods, however *Infernogallus jamesi* takes this further than most oviraptorosaurs [2,3,7]. The ectopterygoid consists of two parts, a pterygoid process and a maxillary process, oriented with their long axes at 90° to each other.

The pterygoid process of the ectopterygoid expands posteriorly in lateral view, forming a flattened cone that cups the anterior margin of the pterygoid buttress and extends a short distance along its dorsal and ventral edges. The lateral surface of the ectopterygoid is depressed to accommodate the pterygoid, so that the ectopterygoid lies against the medial surface of the pterygoid and is minimally exposed laterally. This produces the distinctive crescentic suture between the pterygoid and ectopterygoid in lateral view that characterizes most oviraptorosaurs [2,3,8,9].

The maxillary process of the ectopterygoid of *Infernogallus jamesi* is reduced and simplified compared to other oviraptorosaurs. Whereas in *Incisivosaurus* and oviraptorids the dorsal end of the maxillary process is expanded into a hatchet-like process that bolsters the maxilla where it meets the jugal and lacrimal [3,7], in *Infernogallus* this process is thin and rod-like, with a slightly clubbed dorsal end. This appearance is consistent on both sides of the skull.

Palatine—The palatine is a complex bone that is difficult to observe in most oviraptorosaur skulls because it is typically preserved only in articulation with the overlying pterygoid and ectopterygoid. It typically consists of two parts, a platelike posterior region with contacts for the vomer and pterygoid, an anterior process that contacts the maxilla.

Whereas usually in oviraptorosaurs the platelike region is oriented transversely to span the gap between the pterygoid and vomer [2], from µCT scans of ROM VP 65050, it appears to be oriented more vertically in *Infernogallus jamesi*. The contact with the vomer cannot be discerned because there is a gap between these bones as preserved, but it appears based on the position of the vomer that this contact was situated close to the midpoint of the palatine, internal to the rod-like maxillary process of the ectopterygoid, similar to oviraptorids [3,4]. Posterior to this, the platelike portion of the palatine is expanded dorsally, extending as far as the tip of the ectopterygoid. From this point, the vertical plate of bone tapers posteriorly before it contacts the anterior end of the pterygoid, and it appears to match the concavity in the dorsal surface of the pterygoid and contribute to a dorsal pocket on the lateral shelf of the palate.

The maxillary process of the palatine is more conventional for an oviraptorosaur, in being expanded into a broad triangular plate, but this is relatively larger in *Infernogallus jamesi*, and offset from the rest of the palatine by a less well-developed neck. The triangular plate is thicker anteriorly, forming a rounded columnar ridge where it underlies the maxilla and extends the entire length of the antorbital fossa.

Vomer—The oviraptorosaur vomer is a rod-like bone with expanded anterior and posterior ends, roughly resembling a dumbbell [2,4]. Typically, it extends anteroposteriorly parallel to the ectopterygoid–pterygoid bar [2,4], but in *Infernogallus jamesi*, it is steeply inclined posterodorsal–to–anteroventrally. The posterodorsal end is expanded dorsoventrally but relatively constricted mediolaterally, not being much thicker transversely than the shaft. The anteroventral end, in contrast, is expanded into a bulb, which appears to be filled with cancellous cavities in µCT data. This anterior end contacts the maxilla, like in oviraptorids, to produce a 'tooth-like' maxillovomeral process [4].

Palatal part of the maxilla—The palatal portion of the maxilla is difficult to observe in ROM VP 65050, although its anterior part is exposed on the right side of the skull. The µCT data confirm the presence of this ventral process, but crushing obscures finer details like the presence of ridges and grooves sometimes described for oviraptorids. As preserved, the palatal process of the maxilla in *Infernogallus jamesi* appears similar to that in *Citipati osmolskae* [2], with a relatively long, triangular spine extending ventrally. The exposed right side of the skull and the µCT data show that this spine extends along the anterior edge of the palatine, and was possibly reinforced by this contact. There appears to be at least one prominent, posteriorly curving dorsoventral ridge along the anterolateral surface of the palatal process of the maxilla. The palatal process of the maxilla of *Infernogallus jamesi* appears to differ from the less protruding process of *Epichirostenotes curriei* [10], although the base of this process is broken in *Epichirostenotes curriei* (ROM VP 43250) and the process may have been much longer in life. Nevertheless, other aspects of the maxilla differ considerably between *Infernogallus jamesi* and *Epichirostenotes curriei*, suggesting that the shapes of their beaks and oral cavities may have been different; perhaps corresponding to the shallow and deep beaked morphotypes previously characterized for caenagnathids [8,11–13].

Supplementary Note 2: Pterygoid buttresses in crocodylians and caenagnathids

The dorsoventrally compressed skulls of crocodylians result in major changes to their jaw adductor musculature, possessing large anteroposteriorly oriented pterygoideus muscles with anteroposterior vectors to drive mandible closure [14]. These large pterygoideus muscles are supported by ventrally protruding pterygoids, that project into the oral cavity and between the mandibles when the jaws are closed, and contribute to jaw stabilization against mediolateral movement [6]. However, the close proximity of the pterygoid to the medial surface of the lower jaw results in compressive forces on the adductor musculature during jaw movement [6]. To mitigate these compressive forces on soft tissues, crocodylians develop extensive cartilage-covered bony pads on the lateral surface of the pterygoid, the pterygoid buttress, and have an incompressible fibrocartilage sesamoid (the cartilago transiliens) that passes along the pterygoid buttress during the chewing stroke [6]. This specialized arrangement of soft tissues leaves osteological correlates in extant and extinct crocodylians: the corresponding surface of the pterygoid buttress is cartilage-covered, whereas the corresponding coronoid eminence on the mandible is rugose [15].

These features are remarkably similar to the palatal bones of *Infernogallus jamesi*. The pterygoid possesses a large, rugose lateral buttress that is not present to the same degree in other oviraptorosaurs, with a surface texture consistent with a cartilage cap [16]. The medial surface of the coronoid eminence of the surangular in ROM VP 64193 exhibits a rugose and fibrous texture, and this is generally true of caenagnathids more broadly. However, the orientation of these features differs between *Infernogallus jamesi* and crocodylians. Whereas in extant crocodylians the pterygoid buttress is oriented with its long axis anterodorsal to posteroventral (e.g., ROM R9549 *Caiman crocodilius*), in *Infernogallus jamesi* this feature is oriented with its long axis anteroposterior. In crocodylians, the long axis of the buttress closely matches the path of the coronoid as it passes by the pterygoid during jaw movement, and the pseudotemporalis superficialis muscle and cartilago transiliens stretch over the pterygoid buttress during mandibular depression. The anteroposterior orientation of this buttress in *Infernogallus jamesi* may therefore suggest that the coronoid eminence of the mandible traveled anteriorly and posteriorly past the pterygoid at least some of the time, consistent with a palinal chewing stroke inferred from the jaw adductor musculature (see below).

Supplementary Note 3: Reconstructing the chewing stroke in *Infernogallus jamesi*

Based on the complete oral apparatus of ROM VP 65050 and the jaw adductor vectors, a hypothetical chewing cycle can be proposed for *Infernogallus jamesi* and other deep-beaked eucaenagnathines. The articular ridge of the mandible and the saddle-shaped articulation of the quadrate would have allowed the mandible to slide anteroposteriorly at least 15 mm in ROM VP 65050, assuming that the articular ridge would have had to remain between the cotyles of the quadrate. The long retroarticular process of the mandible and posterior process of the squamosal would have allowed *m depressor mandibulae* to open the mouth at both the anterior and posterior extremes of this range of movement. Therefore, the chewing stroke may have begun in a variety of positions, which may have been advantageous for fine manipulation of food.

However, based on the shape of the articular ridge, which would remain in closer contact with the quadrate articulation when opening the mouth posteriorly, and the close alignment of

the anterior edges of the dentaries and premaxillae, we tentatively interpret a posterior position as a more typical starting point for the chewing stroke. After depression of the mandible in a posterior position, contraction of the *pterygoideus* muscles would have brought the mandible forwards and slightly upwards, shifting the vectors of the temporal musculature further anterior (the proal part of the stroke). Contraction of the temporal musculature would close the mouth and bring the mandible upwards and backwards, driving the medial sulcus of the dentary into the maxillo-vomeral process (the palinal part of the stroke). Notably, this movement would happen approximately parallel to the long axis of the vomer, which itself is parallel to the vectors of the temporal adductor musculature. In this closed position, it is possible that the *pterygoideus* muscles and temporal adductor musculature could be sequentially contracted to move the mandible back and forth, crushing food trapped between the dentaries and maxillo-vomeral process. However, it is unclear if this would be as efficient as performing another chewing stroke, as both muscle groups would have less capability to contract further.

Supplementary Note 4: Phylogenetic results

Extensive revisions to the most frequently used oviraptorosaur phylogenetic matrix [3,12,17–19] provide clearer, more stable resolution of caenagnathid relationships (Fig. 4; Supplementary Figs. 11, 12). Three focal analyses were undertaken on this matrix: a conventional parsimony analysis, a Bayesian analysis, and a relaxed-clock, tip-dated Bayesian analysis.

Parsimony—The parsimony analysis recovered 30 most parsimonious trees of 745 steps. A monophyletic Caenagnathidae (the common ancestor of *Microvenator* and *Elmisaurus*, and all of its descendants) is supported in the strict consensus (Supplementary Fig. 11a), as is a group of late-branching 'deep-beaked' caenagnathids, including *Infernogallus* alongside *Apatoraptor, Chirostenotes*, *Citipes*, *Elmisaurus*, *Eoneophron*, and *Hagryphus*. Within this clade, *Apatoraptor* is basal to the rest, and *Hagryphus* is recovered as a wildcard taxon; when it is removed, *Chirostenotes* moves basal to the remaining four taxa, and *Citipes* and *Elmisaurus* become sister taxa. The sister pairing of *Anzu* and *Caenagnathus* are united with the 'deep-beaked' taxa in a relatively strongly supported clade (Bremer decay value 2), here called Eucaenagnathinae. Remarkably, *Epichirostenotes* is recovered outside of Eucaenagnathinae, in a polytomy with *Anomalipes* and *Caenagnathasia*. When *Anomalipes* (identified as a wildcard taxon) is removed, *Epichirostenotes* becomes sister to Eucaenagnathinae.

Bayesian topology—In the standard, non-clock Bayesian analysis, the majority rules consensus has little resolution. However, the maximum posterior probability tree (Supplementary Fig. 11b) is generally consistent with the parsimony tree, regardless of whether the analysis was constrained, although there are some notable differences. On the oviraptorid side, whereas the parsimony analysis does not recover a monophyletic Citipatiinae, the Bayesian analysis does, although with different membership than recovered in Funston et al. (2020). Within Caenagnathidae, both analyses recover a monophyletic Eucaenagnathinae, although internal relationships are different. *Infernogallus jamesi* is recovered in a clade with *Eoneophron infernalis*, *Elmisaurus rarus*, *Citipes elegans*, and *Apatoraptor pennatus*, similar to some previous parsimony-based results. Most notably, though, is that *Epichirostenotes* is recovered as a member of Eucaenagnathinae in the Bayesian analysis, and with *Chirostenotes* and *Hagryphus*, it forms a sister clade to the pairing of *Anzu wyliei* and *Caenagnathus collinsi*. Therefore, the two

analyses differ in whether *Chirostenotes* and *Hagryphus* are allied with 'deep-beaked' caenagnathids, but both consistently recover *Apatoraptor*, *Citipes*, *Elmisaurus*, *Eoneophron*, and *Infernogallus* in a monophyletic group. The maximum clade credibility (MCC) tree has some unusual features. Notably, *Xingtianosaurus ganqi* is recovered between avimimids and caenagnathoids, despite its clear affinities with caudipterygid-grade oviraptorosaurs. Instead of a bifurcation within Eucaenagnathinae, eucaenagnathines form a grade, with only three pairs of sister taxa: the basal *Chirostenotes pergracilis* and *Hagryphus giganteus*, intermediate *Citipes elegans* and *Elmisaurus rarus*, and late-branching *Anzu wyliei* and *Caenagnathus collinsi*. Perplexingly, other taxa, including *Infernogallus jamesi*, fall along the stems in between these three sister taxa. Overall, the MCC tree is markedly different in topology from the tree with the highest posterior probability, and also with the parsimony results. Furthermore, it is somewhat inconsistent with our current morphological and stratigraphic understanding of caenagnathids. For this reason, we prefer the maximum posterior probability tree, which is defensible on the grounds that it is the single most likely tree recovered by our sample.

Relaxed clock topology—Both of the relaxed clock analyses (3- or 4-rate skyline FBD models) produced results that are highly consistent with previous analyses of oviraptorosaur phylogeny and with each other. The MCC trees of each analysis are identical in topology, and the highest posterior probability trees have the same topology. Our preferred tree is therefore the highest posterior probability tree from our 4-rate analysis (Supplementary Fig. 12), because it incorporates the most detailed model and retains a high likelihood. Eucaenagnathinae is monophyletic and divides basally into a clade of *Anzu wyliei* and *Apatoraptor pennatus*, opposed with *Chirostenotes pergracilis* and the sister pair of *Infernogallus jamesi* and *Elmisaurus rarus*. The model for our preferred tree estimates the origin of Caenagnathoidea between 129.4–110.1 Ma (mean = 118.7, median = 117.9), the origin of Oviraptoridae between 91.8–80.4 Ma (mean = 85.8, median = 85.5), the origin of Caenagnathidae between 120.2–108.3 Ma (mean = 114.0, median = 113.6), and the origin of Eucaenagnathinae between 84.3–76.7 Ma (mean = 80.3, median = 80.0). Critically, both the 3- and 4-rate analyses produce concordant estimates of origination dates across the tree, with differences in mean and median ages generally < 1 Ma and often closer to ± 0.1–0.2 Ma. The most significant difference among major origination dates is that the 3-rate tree infers slightly earlier origination dates for Oviraptoridae (mean = 86.9, median = 86.7) compared to the 4-rate tree (a maximum difference of 1.2 Ma).

Eucaenagnathine subclades and 'Elmisaurines'—The monophyly of 'elmisaurines' has been debated with little resolution, as previous phylogenies, like ours, vacillate with regards to which species are included [11,12,17,20]. Critically, we find little evidence from our phylogenetic analyses of any consistent subgroupings of eucaenagnathines, with the exception of already-recognized sister taxa, like *Caenagnathus collinsi* and *Anzu wyliei* or *Citipes elegans* and *Elmisaurus rarus*, and these are likely driven to some degree by uniquely overlapping skeletal elements. Notably, only our parsimony results support a division between 'deep-' and 'shallow-beaked' caenagnathids, but there is no resolution of relationships among 'deep-beaked caenagnathids'. In the other analyses, 'deep-beaked' caenagnathids are paraphyletic with respect to 'shallow-beaked' caenagnathids, and thus there is no basis for inferring that 'deep-beaked' caenagnathids are more closely related to each other than to 'shallow-beaked' caenagnathids. It is also noteworthy that all of the consistent sister clades recovered correspond to pairs where one taxon is represented solely or overwhelmingly by a single skeletal region: *Hagryphus* is known

only from the hand (also known in *Chirostenotes*) [11,21–23], *Caenagnathus* is known mostly from the mandible (also known in *Anzu*) [11,17,24,25], and *Citipes* is known primarily from the feet (also known in *Elmisaurus*) [13,26–28]. Although each of these sister pairs is supported by distinctive shared features, these uniting characters do little to resolve broader relationships within Eucaenagnathinae, and it is not clear that any co-occurring characters in these taxa (e.g., 'deep' beaks and fused tarsometatarsi) can be generalized as morphotypes to other species where some of these characters are unknown. Altogether, our analyses reinforce the ephemerality of eucaenagnathine subclades, and caution that formal subclades should not be erected until a better understanding of the distribution of morphological characters is developed.

Supplementary Note 5: Additional comparison to extant analogues

The features of eucaenagnathine dentaries are distinctive compared to living edentulous species, but analogues of their component parts are present in other groups. For example, the configuration of the beak-like dentaries, with a scalloped tomium and longitudinal ridges separated by a midline sulcus (Supplementary Figs. 3, 4) [3,24,29], bears remarkable morphological similarity to certain turtles, which suggests that they have shared function. In particular, herbivorous turtles (but not durophages or carnivores) possess triturating ridges on the maxillae for processing plants [30] that are analogous to the lingual ridges of caenagnathid dentaries. An especially striking analogue for caenagnathid beaks can be found in *Chelonia mydas* (Supplementary Fig. 10d), which has triturating ridges separated by a midline sulcus that accommodates a tubercle on the countering bone, directly equivalent to the distinctive palatal morphology revealed in *Infernogallus jamesi* (Supplementary Fig. 10c,d). Considering that these complex ridges and grooves of the dentary in caenagnathids would have supported a keratinous rhamphotheca [31,32], which enhances the relief of these structures in *Chelonia mydas*, these similarities likely indicate a similar emphasis on oral food processing.. Indeed, many herbivorous turtles consume a high proportion of soft foods like fruit, which they mash orally, and *Chelonia mydas* specializes on soft sea grasses, which it orally processes with a palinal chewing motion [33]. Some birds also exhibit textural features on the beaks, although these are not reflected on the underlying bone. In *Strigops* and *Nestor* (Psittaciformes), ridges on the inside of the beak, somewhat akin to the lateral occlusal ridges of caenagnathids, assist in abrading fibrous plant material, including dry-fleshed gymnosperm fruit [34].

Mandibular mobility, enabling anteroposterior movement during the chewing stroke, is rare in edentulous species. Among avians, articular structures that allow mobility are best developed in Psittaciformes, although they are present to some degree in *Gallus gallus* and *Anas platyrhynchos* [20,35]. Likewise, some turtles show incipient features conferring mobility, including *Chelonia mydas* (Supplementary Fig. 10a,b). Mandibular mobility is more common in toothed species, and has been demonstrated in a wide range of tetrapods, from salamanders to sphenodontians, to crocodylians, to mammals [36–46]. To our knowledge, in all cases, mandibular mobility is associated with increased oral processing capabilities, and is more typical of herbivory than of other diets (but not exclusively). The articular features enabling motion in *Infernogallus jamesi* are as well developed or much better developed than in any of these analogues, suggesting that anteroposterior motion was important for food processing in caenagnathids.

Other aspects of the articular region may also be associated with the enhanced food process capabilities of caenagnathids. The retroarticular process of caenagnathids is elongated

relative to other theropods, including oviraptorids. In birds, an elongated retroarticular process is associated with frugivory in two ways: by facilitating powerful gaping behaviours, and by enhancing gape to accommodate larger food items [47–49]. Although it is unlikely that the short, upturned beaks of caenagnathids would have been useful for gaping behaviours to open plant parts to access fruit, the increased gape conferred by an elongate retroarticular process may have compensated for the protrusion of the palate into the oral cavity. Furthermore, we hypothesize that the elongate retroarticular process of caenagnathids also served to facilitate anteroposterior motion of the mandible during chewing, ensuring that *m depressor mandibulae* was not impinged by the quadrate articulation when the jaw was moved anteriorly.

Our analyses of muscle vectors show a general signal of decreasing dorsoventral musculature orientations in theropods that consume softer foods (Fig. 3c). In general, folivorous and frugivorous birds are grouped together, a pattern which phylogenetically-corrected PCA sensitivity tests show is not the result of phylogeny. Nevertheless, we hesitate to generalize broad signals of diet based on muscle vectors, and instead our analyses are intended to evaluate the similarity of potential analogues in more detail. Developing reliable muscular vector proxies for fine dietary categories (some of which are rare or unique) would require a broader and denser sample of avians, but doing so necessitates dissections (virtual or conventional) to map muscle areas, detailed resolution of homologues, and ecological studies to quantify diet. Some of these data might be available in the literature, but compiling them is beyond the scope of our study here.

Furthermore, our results also highlight the difficulty of distilling the complexity of muscle function, joint morphology, and behaviour into easily quantifiable parameters, even when data are available. Testament to this, we find that *Struthio camelus* exhibits muscle architecture remarkably similar to *Psittrichas fulgidus* (Fig. 3c), despite using virtually no oral processing of food prior to ingestion. We suggest that a key morphological difference accounts for this inconsistency: *Struthio camelus* lacks the saddle-and-ridge articulation that gives *Psittrichas fulgidus* the capacity to move its mandible anteroposteriorly. Similarly, *Eudromia*, also a paleognath, is close in morphospace to both *Struthio* and to *Anas* and *Chauna*. It is unclear whether the close positions of these taxa reflect their dietary similarities (all incorporating some degree of soft water-plant folivory), or whether they result from phylogenetic inertia. Indeed, like with *Struthio* and *Psittrichas*, these taxa appear to vary in the mobility of their mandibles, and thus, despite similar muscular arrangements in these taxa, the resulting functions may be vastly different. This highlights both the difficulty of distilling function down to low-dimensional indices that could support statistical tests, and the profound impact of the combination of these two key features on cranial function.

To build on this idea, we note that whereas some avian theropods have strongly anteroposteriorly oriented jaw muscles, and some non-avian theropods have anteroposteriorly mobile mandibular joints, we were able to find the co-occurrence of these two features in only two theropod taxa: *Infernogallus jamesi* (and potentially other eucaenagnathines), and *Psittrichas fulgidus*. The latter taxon is an obligate frugivore, and these two cranial adaptations are strongly reflective of its unique dietary habits: *Psittrichas fulgidus* defleshes soft fruit using anteroposterior motions of the mandible [50]. Outside of Theropoda, another possible example of this combination of key characters occurs in *Chelonia mydas*, although its cranial muscular organization is not sufficiently known to evaluate the extent of convergence with *Infernogallus jamesi*. Nevertheless, *Chelonia mydas* is an especially herbivorous sea turtle, and its diet, consisting of soft sea grasses, might be considered functionally equivalent to pulp-eating

frugivory in terrestrial edentulous animals, as both diets rely on oral processing of soft vegetation. In this regard, the convergence of three distantly-related edentulous taxa on a similar distinctive organization of the oral apparatus emphasizes these specific adaptations as effective for diets relying on oral processing of soft plant materials.

Supplementary Note 6: Additional evidence of angiosperm-based diets in caenagnathids

In light of evidence of a specialized herbivorous diet based on the cranium, other features of caenagnathid anatomy or palaeobiology may be further circumstantial evidence of specialization for angiosperm-based diets. The caenagnathid bauplan combines a small skull, elongate neck, short torso and tail, and long appendages, a somewhat unusual combination of features.

Small skulls on long necks are frequently considered adaptations for herbivory by increasing feeding range (e.g., in sauropods [51] or giraffes), which seems reasonable for caenagnathids. The short torso of caenagnathids is opposite the expectation for a high-fiber herbivore, which requires long digestion times of tough or fibrous plants. Likewise, gastroliths have not been discovered in any caenagnathid or oviraptorid, despite many articulated skeletons and the presence of a gastric mill in more basally-branching taxa [52]. Caenagnathids in particular have even more reduced gut volumes compared to oviraptorids because of the shapes of the pubes, which are straighter and less concave than oviraptorids [53]. It is possible that sophisticated oral food processing obviated the need for long digestion times, but seeing as these digestion-enhancing abdominal traits were basally present in oviraptorosaurs, their loss in caenagnathids is notable. Instead, this situation is more consistent with a change to easily-digestible food in caenagnathids, but which required oral processing to access.

The elongate, grasping manus, which is potentially perplexing for a herbivorous dinosaur, could have served as a tool for occasional prey capture, but could also have facilitated selection or manipulation of specific plant parts. The laterally-directed shoulder glenoid in some taxa may have enhanced flexibility for these functions, and the elongate second digit of the manus may have been useful for raking fruit into accessible positions.

Whereas the long hindlimbs of caenagnathids could have been adaptations for cursoriality and predator avoidance [54], some evidence suggests that they are more akin in musculature to the long legs of wading birds [53]. This is supported by the elongate, wide splayed toes, which are reminiscent of birds that wade in soft substrates. If angiosperm radiation was initially fostered in marshy environments as hypothesized [55], the hindlimbs of caenagnathids would have facilitated access to these areas—or perhaps may even have evolved in response. Indeed, several studies have commented that the caenagnathid fossil record appears to reflect a preference for more mesic environments [8,17,20], which would be consistent with our current understanding of angiosperm and fruit evolution.

More speculative, some features of oviraptorosaurs may be indirectly related to—or have resulted from—consumption of angiosperm plants. Oviraptorosaurs have the most complex feathers outside of Aves [56,57], and several lines of evidence suggest they had multiple interspecific signalling structures, including pennibrachia and pygostyles [12]. Many extant birds consume pigments in fruit that contribute to the colouration of their feathers [58], providing an honest interspecific signal of health. Evidence from some oviraptorosaurs shows that feather complexity increased through ontogeny in oviraptorosaurs [57], suggesting a role in mating displays. Consuming pigment-laden fruit could have contributed to mating displays in oviraptorosaurs, as it does in extant birds, and this could have driven the proliferation of traits

which appear to be related to sexual display in oviraptorosaurs. Further testing of feather pigmentation in oviraptorosaurs—and its evolution—could support this notion.

Supplementary Note 7: Palatal evolution in Oviraptorosauria

The palatal complex in oviraptorosaurs has long been recognized as specialized compared to other theropod dinosaurs. One of the earliest changes to the palate, in basal oviraptorosaurs like *Incisivosaurus*, is the anterior shift of the ectopterygoid, so that it lies anterior to the pterygoid rather than lateral [7,59]. Thus, the pterygoid is elongate anteroposteriorly, with its ectopterygoid and palatine processes positioned close to each other at the anterior end of the element. This shift in the position of the ectopterygoid and its contact on the pterygoid results in a small transverse shelf at the anterior end of the pterygoid, and the development of an ectopterygoid-pterygoid bar that runs longitudinally parallel to the midline. In *Incisivosaurus*, the ectopterygoid has already assumed the distinctive shape of oviraptorosaurs, what Osmólska et al.[3] (2004) described as similar in shape to the bow of a "Viking ship". The ectopterygoid tapers anteriorly and curves strongly dorsally, before expanding again anterodorsally into a roughly triangular wedge of bone. The overall shape thus resembles a curved hourglass, with a constricted middle portion and triangular ends—Osmólska's "Viking ship" description was apt. Together with the maxillary process of the palatine, the ectopterygoid arches upwards from the palatal bar to support the maxilla where it contacts the jugal and lacrimal.

The palate is poorly known from oviraptorosaurs between the Barremian and the Campanian, mirroring the poor record of theropods overall during this interval. However, a notable exception is the earliest caenagnathid, *Microvenator celer*, from the Aptian–Albian Cloverly Formation in Montana, USA. Preliminary re-examination of the holotype material (AMNH 3041) reveals a suite of previously unidentified but well-preserved cranial bones, including a pterygoid, recognizable based on its similarity to oviraptorids and the newly recognized pterygoid morphology of *Infernogallus*. The pterygoid of *Microvenator* has a fan-shaped posterior end, with a slight depression on the medial surface which probably contacted the basipterygoid process of the basisphenoid as in other oviraptorosaurs. Unlike more basal oviraptorosaurs, however, the ramus of the pterygoid descends anteroventrally from the pterygoid wing, rather than anterior to it, a character that is much more similar to *Infernogallus* than to *Incisivosaurus* or oviraptorids. The ramus has an approximately elliptical cross-section, with a subtle lateral ridge. Like in basal oviraptorosaurs, the anterior end of the pterygoid is developed into a transverse shelf, but this differs in some key respects from *Incisivosaurus*. The transverse shelf is much longer relative to the overall length of the pterygoid, and instead of being flat, it is concave both dorsally and ventrally. The lateral end of the shelf is broadened into a lateral buttress, remarkably similar to *Infernogallus jamesi* but much larger than this plate in either *Incisivosaurus* or oviraptorids. The medial process is crushed into the shelf in AMNH 3041, implying that this shelf would have been broader in life than as preserved. Overall, despite being much closer in age to *Incisivosaurus*, the pterygoid of *Microvenator* is much more similar to the latest Cretaceous *Infernogallus*, implying rapid initial change followed by relative morphological stasis.

Like earlier-diverging oviraptorosaurs, Late Cretaceous oviraptorids retain the distinctive arrangement of the palatal bones [2,3,60,61], in which the pterygoid and ectopterygoid are aligned anteroposteriorly and form a longitudinal bar. In this arrangement, the pterygoid transitions from a fan-shaped posterodorsal wing through a thickened circular ramus, into a transversely oriented

shelf at its anterior end. In oviraptorids, the ramus of the pterygoid usually extends anteriorly from the wing, in some cases curving gently ventrally before extending horizontally. The pterygoid shelf is often transversely narrow, and if it is expanded, this is only for a short anteroposterior distance, typically less than half the total anteroposterior length of the pterygoid. In taxa where it can be observed, the shelf is usually bordered on either side by rounded longitudinal ridges of bone; on the medial side, this ridge extends anteriorly to contact the vomer close to the midline. The lateral ridge contacts the ectopterygoid, and in many oviraptorids this ridge protrudes ventrally near this contact into a small flange. The palatal bar (composed of the pterygoid, ectopterygoid, palatine, and vomer) is often aligned with a plane between the ventral premaxilla and quadrate articulation. Thus, this bar forms the bottom of the skull, but it rarely descends below other bones of the skull. Avimimids appear to possess the same features of the palate as oviraptorids, although very little is known about the avimimid palate and sutures are difficult to discern because of extensive fusion of the cranium [62–64].

Thus, the palates of Late Cretaceous caenagnathids (described in full above) diverged from basal oviraptorosaurs, whereas oviraptorids retained an arrangement similar to *Incisivosaurus*. The incipient development of some of the striking caenagnathid features in *Microvenator*, like the ventral curvature of the pterygoid, the expansion of the transverse shelf, and the large lateral buttress, suggests that caenagnathids functionally diverged from other oviraptorosaurs some 50 million years before *Infernogallus*. Nevertheless, the pterygoid of *Infernogallus* shows further development of the trends established in *Microvenator*, especially the much longer and broader transverse shelf to anchor the pterygoideus muscles. This suggests that the unusual biomechanical features shown in *Infernogallus* were established early in the caenagnathid lineage, but that they continued to be refined through time.

Supplementary Note 8: Diet in other oviraptorosaurs

The lines of evidence supporting frugivorous adaptations in *Infernogallus jamesi* do not necessarily hold for all caenagnathids nor other oviraptorosaurs more broadly. Thus, it is unlikely that many or all other oviraptorosaurs were all specialized frugivores, although there is circumstantial evidence that they may have been specialized for angiosperm-based diets nonetheless.

Early oviraptorosaurs are widely considered to have been herbivorous, but their exact diets are unclear. Functional morphological studies of *Incisivosaurus* and oviraptorids show that these oviraptorosaurs had well-muscled, biomechanically strong skulls, which were adaptations for a powerful bite [65–67]. Notably, cranial dynamics in *Incisivosaurus* are more similar to oviraptorids than they are to other herbivorous theropods [66] (Fig. 3), suggesting that even the earliest oviraptorosaurs had distinctive dietary ecologies. In *Incisivosaurus*, the incisiform teeth and resistance of the skull to twisting motions [66] suggest a reliance on disassembling food using a strong nipping bite, and this is consistent with herbivory of durable plant parts like shoots, stems, and possibly early angiosperm fruits or seeds: achenes, berries, and follicles show up in the fossil record in the late Barremian at the same time as *Incisivosaurus*, Nipping or twisting motions attested to by skull biomechanics could have been used to strip fruits and seeds from angiosperm shrubs.

The characteristics in *Incisivosaurus* that facilitated a cropping or nipping bite are further emphasized in oviraptorids, and some oviraptorids show evidence of a shift away from oral food processing and a de-emphasis of anteroposterior movement of the mandible (e.g., *Citipati*

osmolskae [65,66]). These features have led to suggestions that oviraptorids in particular were more strongly adapted for eating durable plant parts, like stems, nuts, or seeds [67]. The strong convergence of oviraptorid and psittaciform cranial shape, particularly psittaciform species like *Callocephalon fimbriatus* and the genus *Ara,* which specialize on durable foods [68–70], supports this assertion. Many of the features of the beak of psittaciforms are interpreted to be adaptations for processing durable foods like seeds and nuts [69]. Likewise, the oviraptorid record aligns closely with that of nuts, which seem to first appear in angiosperms in the Coniacian [71], just before the estimated origin of Oviraptoridae and their rapid Campanian diversification (Fig. 4 and Supplementary Text above). It is important to note that the absence of oviraptorids in the fossil record before this point is relatively well-supported. Fossiliferous deposits of Santonian age (Bayanshiree Fm., Iren Dabasu Fm.) conspicuously lack oviraptorids, despite preserving other oviraptorosaur groups, including caenagnathids of varying sizes [72,73] and avimimids [74]. Based on our phylogeny, some ancestral lineage of oviraptorids must have diverged from caenagnathids by the late Aptian, but it appears that this lineage was not highly diverse before the Campanian. In summary, adaptations of the oviraptorid cranium are more consistent with a diet emphasizing durable plant parts, and are particularly well-suited for nuts, which arose shortly before oviraptorids explosively diversified.

Understanding the diets of avimimids is difficult based on their sparse fossil record. Avimimids exhibit similar craniomandibular configurations to oviraptorids [62,63], and therefore probably shared similar functional ecology. The date at which avimimids first appear in the fossil record is unclear. The two candidate records which appear to be the oldest are an isolated occurrence in the Yalovach Formation in Tajikistan [75] and a bonebed from the Iren Dabasu Formation in China [74]. The former formation has not been dated using radioisotopic dating, as suitable sediments are not available, but the maximum age of the Iren Dabasu Fm. (= Erlian Fm.) has been constrained to 95.8 ± 6.2 Ma on the basis of detrital zircon dates [76]. In any case, both formations have been correlated mainly using vertebrate biostratigraphy as Santonian in age (see review in [74]). However, other biostratigraphic methods [77,78] point to a later date for at least the Iren Dabasu Formation, possibly late Campanian–Maastrichtian. Thus, although it is clear that avimimids originated by the Campanian at the latest, they may possibly have an older record stretching back to the Santonian (and either way, a considerable ghost lineage).

It is likely that caenagnathids also exhibited dietary differences within the clade. The presence of *Infernogallus*-like pterygoids in *Microvenator* suggests that it shared similar muscular adaptations for oral food processing, and its dentaries likewise show incipient development of the shelf and ridges that characterize eucaenagnathine dentaries [79]. Notably, *Microvenator* is known from the Albian, about 11–18 Ma after the origin of fruit in angiosperm, and only 3–10 Ma after the appearance of the first drupes [71]. *Caenagnathasia martinsoni* is possibly the next oldest caenagnathid, known from the Bissekty and Iren Dabasu Formations in Uzbekistan and China, respectively [24,80,81]. The age of the Bissekty Formation is typically given as Turonian [82–84], despite early work suggesting a younger age [85]. This older age appears to arise from a statement in Archibald and Averianov [82], referring to a paper in press by King *et al.* which was ultimately never published. Other attributions of a Turonian age cite personal communications from C. King, who unfortunately has since passed away. However, recent work using ammonites now further supports a Turonian age [86]. *Caenagnathasia* is also known from the Iren Dabasu Formation, which, as discussed above, may be younger than estimated from vertebrate biostratigraphy. Thus, there is significant stratigraphic uncertainty (> 25 Ma) in the age of *Caenagnathasia martinsoni*, a species which, as currently understood, could extend from

the Turonian to the Maastrichtian. Nevertheless, its dentary morphology is remarkably similar to eucaenagnathines, in spite of its much smaller body size (< 5 kg), suggesting that it likely possessed similar oral processing adaptations as in later caenagnathids. At around the same time, caenagnathids also became enormous, represented by *Gigantoraptor* (> 1800 kg), known likewise from the Iren Dabasu Formation, but also by similar gigantic material in the Cenomanian–Santonian Bayanshiree Formation in Mongolia [72,73,87]. The dentaries of *Gigantoraptor* do not show the ridges and grooves in eucaenagnathines and *Caenagnathasia*, and are in some respects more similar to oviraptorids, suggesting a more powerful bite and less of a reliance on oral food processing. Fruits are reasonable foodstuffs for the small-bodied *Microvenator* and *Caenagnathasia*, which also show evidence for oral food processing from the dentaries. However, it is difficult to conceive of how an animal the size of *Gigantoraptor* could be sustained from a predominantly fruit-based diet, although very large seeds (i.e., coconuts, >50 mm pyrene length) are inferred to have arisen in Arecaceae in Asia between the Turonian and Santonian [88]. Instead, *Gigantoraptor* likely diverged in diet from other smaller oviraptorosaurs.

The dichotomous morphologies of eucaenagnathine beaks have long been recognized [11,17,24,29], with a distinction made between 'shallow-beaked' caenagnathids like *Caenagnathus collinsi* and *Anzu wyliei*, and 'deep-beaked' caenagnathids like *Chirostenotes pergracilis* and *Infernogallus jamesi*. In the phylogenetic matrix provided here, this distinction is coded in character 101, Dentary: curvature around the anteroventral "chin" of the dentary in lateral view [0, Chin is abruptly curved so that it appears squarish; 1, curvature is flattened or reduced so that the beak projects anteriorly; 2, curvature is gentle so that the chin appears rounded]. 'Shallow-beaked' caenagnathids possess state 1 for this character, whereas 'deep-beaked' caenagnathids may exhibit either states 0 or 2, but also exhibit character state 1 for character 98 [dentary proportionally short and deep, with maximum depth of dentary between 25% and 50% of dentary length (with length measured from the tip of the jaw to the end of the posterodorsal process)]. This character is treated as unordered.

Critically, most phylogenetic analyses, including those presented here, find that 'shallow-beaked' caenagnathids form a clade within paraphyletic 'deep-beaked' caenagnathids. It is therefore unclear whether certain taxa, like *Hagryphus giganteus* and *Epichirostenotes curriei*, possess shallow or deep beaks. Despite ambiguity regarding the phylogenetic distribution of beak morphologies, there does appear to be a correspondence between beak morphology and body size. The two definitive shallow-beaked caenagnathids are the largest eucaenagnathines known, and comparison of the maxilla of the relatively large *Epichirostenotes curriei* to *Anzu wyliei* suggests that it may have been more likely to have been shallow-beaked as well. In contrast, deep-beaked eucaenagnathines tend to be smaller-bodied, and the largest deep-beaked species known is *Chirostenotes curriei*, estimated to be ~66 kg, less than one quarter the mass of *Caenagnathus collinsi* (~275 kg)[11]. Unfortunately, the data are insufficient to robustly test whether this character distribution is driven by allometric growth (i.e., wherein beaks become shallower with increasing size), because there are too few shallow-beaked taxa known (n = 2). Considering that the morphology of the dentary beak plays an important role in the oral food processing capabilities of *Infernogallus*, it is likely that the divergence of shallow-beaked eucaenagnathies implies that they had a different diet (possibly increased folivory), as has been argued before [11,17,20]. The preserved portions of the pterygoids in *Anzu* [17] are generally similar to those of *Infernogallus*, suggesting that they may also have anchored enlarged *pterygoideus* muscles. However, more data on the temporal region and palate of 'shallow-beaked' eucaenagnathines is required before their dietary adaptations can be determined with confidence.

Based on cranial adaptations and our phylogeny, it is relatively straightforward to envision the evolutionary pathway of dietary changes in oviraptorosaurs. Early oviraptorosaurs, like caudipterygids, were probably adapted for consuming durable plant parts, which they processed with teeth and/or gastroliths. These plant foods could have included dry 'fruits' of gymnosperms, or seeds of either gymnosperms or angiosperms. Inheriting diets focused on these small, high-nutrition plant foods, Caenagnathoidea could have diverged to specialize on either softer foods (fruits; caenagnathids), or more durable foods (nuts; oviraptorids) as these resources became more abundant and widespread in the later Cretaceous.

Altogether, evidence from morphology, biomechanics, and stratigraphy provides a strong case for frugivorous adaptations in some caenagnathids like *Infernogallus*, but it is likely that their diet included a myriad of plant parts, and probably animal resources as well. Even the most frugivorous vertebrates today also consume other resources [89], especially if source plants only bear fruit seasonally. Furthermore, once the enhanced plant processing capabilities of the caenagnathid cranium and mandible arose, they could have facilitated a wide range of herbivorous diets (as suggested for edentulous beaks more broadly[90]), which may or may not have included angiosperms.

Supplementary Note 9: Notes on quantitative approaches

The oral food processing apparatus is a highly complex system in vertebrates, involving interplay between muscular organization, skull shape, and other constraints imposed by the multifunctional nature of the skull. We found through our analytical process that muscle vectors were the best available reflection of diet that could be comparable across the clades of interest, although they remain imperfect and their variation is subject to multiple influences. In particular, the dietary signal in the musculature is complicated by: 1) the fundamental differences in cranial architecture between birds and their theropod ancestors; 2) vagueness in dietary categories of extant avians, which include numerous behaviours; and 3) the relatively few times that flesh-processing frugivory has evolved in birds, limiting comparative replicates and statistical power. These are barriers which a human observer can qualitatively tease apart and interrogate reasonably well, but which are difficult to address quantitatively, especially at the relatively small sample sizes that are dictated by our data. For example, visualization of our data using ternary plots and principal components analysis demonstrates that *Infernogallus* has a muscle vector organization that is distinct from other non-avian dinosaurs, and the region of space it occupies relative to other non-avian theropods is equivalent to the space that soft-plant specialist birds occupy relative to other birds. Furthermore, *Infernogallus* morphologically converges with some birds in the changes to the shape of the skull and mandible relative to its ancestors (and in some qualitative aspects like a sliding jaw joint), but because the underlying bony anatomy is different, it is challenging to quantify this similarity. Thus, although these concepts are clear to an observer who understands the context of the data plots and anatomy, the separate spaces occupied by the birds and dinosaurs make it difficult to apply statistics to this trend, even with phylogenetic correction.

Nevertheless, to evaluate statistical support for our hypothesis, we present three different approaches, outlined below. As we discuss above and subsequently, we feel that these analyses are inadequate to fully address the question at hand, but we show that they nonetheless support our interpretations that: 1) *Infernogallus* exhibits distinct muscular organization relative to other

theropods; 2) There is relatively strong support that this distinct muscular organization is associated with specialization for processing soft plant foods.

We used ordination techniques to distil dietary signal from muscular vectors, and predict diets using these signals: 1) phylogenetic flexible discriminant analysis (pFDA); 2) linear discriminant analysis (LDA); and 3) phylogenetically-corrected LDA in two modes. We used the following dietary categories for the extant birds in our dataset: "soft" for species that primarily eat soft plants (*Psittrichas*, *Anas*, *Chauna*); "seed" for granivores that processed seeds (*Psittacus*, *Geospiza*); "omni" for omnivorous taxa or those with very broad diets and minimal oral food processing (*Gallus, Struthio, Corvus, Eudromia*); and "meat" for carnivores (*Buteo*).

pFDA—We used the approach outlined by Schmitz & Motani (2011) to categorize dietary signals in birds and predict for the theropods. For this approach, we categorized the birds as above, and left all the dinosaur taxa as 'unknown'. The analysis did a poor job of discriminating between dietary categories in the extant birds (3 of 10 misclassified in the training set), and predicted almost all theropods as "omnivores" with exceedingly high certainty (>95%). *Infernogallus* is the exception: it was least strongly predicted as an omnivore (94.3%), with the remainder of likelihood made up by "soft" plants. We interpret these results as arising from the position of all theropods outside of the range encompassed by birds, along with the poor discrimination of dietary categories in extant avians. This latter limitation probably results from behavioural and functional differences within dietary categories (but which can be resolved by comparative anatomy). Altogether, we feel that, although this result tentatively indicates that *Infernogallus* is the most likely among dinosaurs to have had a soft-plant diet, the unrealistic predictions for other theropod diets (e.g., *Tyrannosaurus* as an omnivore) and poor predictive power within avians indicate this approach is not adequate for our data.

LDA—The second approach was to conduct a conventional LDA, not taking into account phylogenetic relatedness. We performed the LDA on the extant birds for which diet was known, and then predicted dinosaurs into the space (Supplementary Fig. 13a). This approach only minimally reorganized the bird data points compared to the PCA (main text Fig. 3c), demonstrating that the PC axes (which do not consider diet) are generally as effective at visualizing the dietary categories as the LDA axes. Estimating the diet of the dinosaurs in our dataset based on this analysis similarly predicts all species to be omnivores with >85% probability, with the exception of *Infernogallus*, which comes out as only 75% probable to be an omnivore, with the remaining 25% proportion of likelihood being "soft" foods. Visual assessment of the data shows that this pattern is driven by the dinosaurs plotting highly negatively along LD2, in their own region of space, identical to the PCA and pFDA, again with the exception that *Infernogallus* plots closer to the birds. Whereas this supports the idea that *Infernogallus* is as well adapted for soft food as any dinosaur in our dataset, it also shows that the highly derived morphologies of birds are probably not easily translatable to theropod diets without more detailed comparisons.

Phylogenetically corrected LDA—To incorporate phylogeny into the analysis, we conducted a phylogenetically-informed PCA (pPCA) and then performed an LDA on the resulting PC values for each taxon (Supplementary Fig. 13b,c). This is because both LDA and phylogenetic regression correction operate by modifying the variance-covariance matrix between points. At

small sample sizes, these approaches completely overprint each other, preventing their combined use. Therefore, structuring point values using a pPCA and then performing LDA on these data was the most practical way to incorporate both phylogeny and dietary categories into the analysis. After sensitivity analyses of intermediate strategies, we undertook this analysis in two main ways, one which was maximally conservative, and another which incorporated as much information as possible. For both analyses, we performed the pPCA on all of the taxa in our dataset, using the phylogeny and node ages of Prum et al. 2015 for birds, and consensus estimates of node ages for dinosaurs.

For our conservative analysis (Supplementary Fig. 13b), we then performed an LDA using only the birds, with the same dietary categories as above, and we predicted the dinosaurs into this space. This produced results exactly identical to the conventional LDA analysis (see above), except that the polarity of the axes was flipped. This indicates that the relatedness of dinosaurs and birds to each other is not affecting the signal to a great degree. It also reflects the way that LDA overprints the variance-covariance mofidications produced by a phylogenetic correction – while the PC axes may change, the LDA still finds the underlying structure.

For the 'exploratory' analysis (Supplementary Fig. 13c), we used generally-agreed-upon diets for the non-oviraptorosaur dinosaurs in the LDA, to flesh out the sample and predict diets for the oviraptorosaurs. We scored the bird diets as for all the other analysis, but we further categorized *Tyrannosaurus*, *Allosaurus*, and *Dromaeosaurus* as carnivores, and *Erlikosaurus* as a "soft" plant eater (as it has generally been assumed to eat foliage rather than handling seeds). This analysis, again, did not substantially rearrange the points, reinforcing that the PCA and LDA are recovering the same major axes of variation. Whereas this is unsurprising given that we only have 3 variables (because they are 3-dimensional muscle vectors), it also reinforces that the PC axes are effective at visualizing the dietary categories, and that dietary categories are indeed reflected to some degree by the muscle vectors. When predicting the oviraptorosaurs into the LDA space, all oviraptorids and *Incisivosaurus* are predicted to be carnivores, whereas *Infernogallus* is predicted to be a soft-plant eater. This directly supports our hypothesis that *Infernogallus* was adapted for eating soft plant materials like fruits, when accounting for generally accepted diets in other dinosaurs. However, it is noteworthy that although the "soft" category is the most favoured prediction for *Infernogallus*, it is only slightly more likely than the omnivorous category (40.6% vs. 40.3%). The other oviraptorosaurs are much more imbalanced, with carnivory being favoured over other categories by >9%. Indeed, these dietary categories overlap extensively in birds in all of our analyses, demonstrating that non-quantitative signals like mandibular joint mobility and the particular muscles comprising the anteroposterior vs. dorsoventral vector components are critical in discerning diet.

To summarize the results above, the statistical analyses generally corroborate two ideas presented with comparative anatomy and non-statistical analysis: 1) *Infernogallus* had significantly different muscle organization than other theropods, even other oviraptorosaurs; and 2) it is difficult to distil a single index that adequately reflects dietary behaviour in birds, probably as a consequence of both data availability (i.e., lack of detailed behavioural or dietary data), and actual complexity in the controls on adductor muscle vector orientation. Critically, none of these analyses provide any meaningful signal that contradicts our anatomical interpretations or those based on the principal component analysis. Where signals are ambiguous, it is clear that this is caused by limitations in the dataset. For these reasons, despite the support we find in our analysis incorporating the most information, we have decided not to include these

statistical analyses in the main text of the manuscript, because it might falsely imply confidence in these statistical results. With a larger and more detailed extant dataset, it is possible a clearer signal could emerge, and this is likely a fruitful avenue for further study.

Supplementary Table 1. Selected skeletal measurements of ROM 65050.

Region	Measurement	Value (mm)
Skull	Skull length (premaxilla to occipital condyle)	104
	Skull height at lacrimal (jugal to frontal)	49
	Skull height at lacrimal (including palate)	73
	Antorbital skull length	49
	Antorbital fossa length	21.7
	Antorbital fenestra length	12.9
	Antorbital fenestra height	24.1
	Orbit length	45.0
	Orbit height	37.6
	Maximum jugal length	63.0
	Postorbital skull length	36
	Frontal length	30.3
	Parietal length	20.3
	Quadrate height	26
	Maxilla length	34.5
	Maxilla height at anterior end of antorbital fenestra	25.5
	Maximum maxilla height	36.4
Mandible	Dentary length, posterodorsal process	61.3
	Dentary length, posteroventral process	81.6
	Minimum dentary height	17
	Maximum dentary height	32
	Mandible length	122.6
	External mandibular fenestra length	42.7
	External mandibular fenestra height	16.7
	Surangular length	68.0
Axial column	Neck length	485
	Trunk length (scapula to acetabulum)	240
Forelimb	Scapulacoracoid length	121†
	Scapular blade length	90†
	Scapula proximal width	25
	Scapula shaft width	12.4
	Humerus length	100e
	Humerus proximal width	27e
	Humerus deltopectoral crest length	38.4
	Ulna length	73†
	Metacarpal I length	36.2
	Metacarpal II length	66.7
	Metacarpal III length	48.5e
	Manual I-1 length	43.3
	Manual I-2 length	28.3
	Manual II-1 length	49.3
	Manual III-1 length	24.1
	Manual III-2 length	19.8
	Manual III-3 length	27.9
	Manual III-4 length	20.6
Hindlimb	Ilium height above pubic peduncle	55
	Femur length	185
	Femur shaft width anterior to posterior	21.4
	Femur circumference	54
	Metatarsal I	18†
	Pedal II-2	25
	Pedal III-2	23.2
	Pedal III-4	22.1
	Pedal IV-2	19.2

Note: †, incomplete value; e, estimated value.

Supplementary Figures:

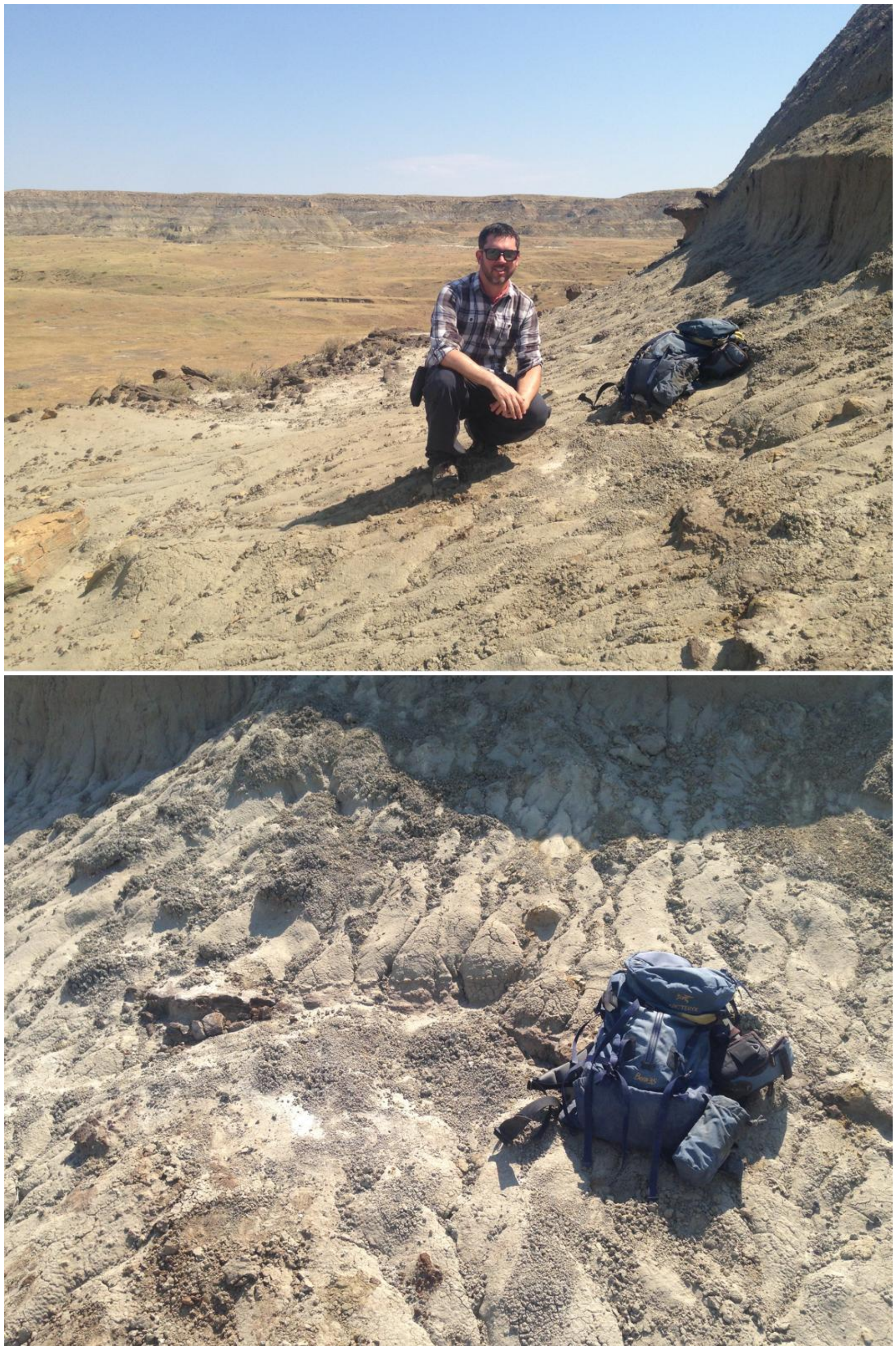

Supplementary Fig. 1. Holotype field locality in the Hell Creek Formation. Photograph (top) of DCE at the quarry where the specimen was collected. Photograph (bottom) of the quarry (to the left of the backpack). The authors affirm that DCE provided informed consent for publication of the image in Supplementary Figure 1.

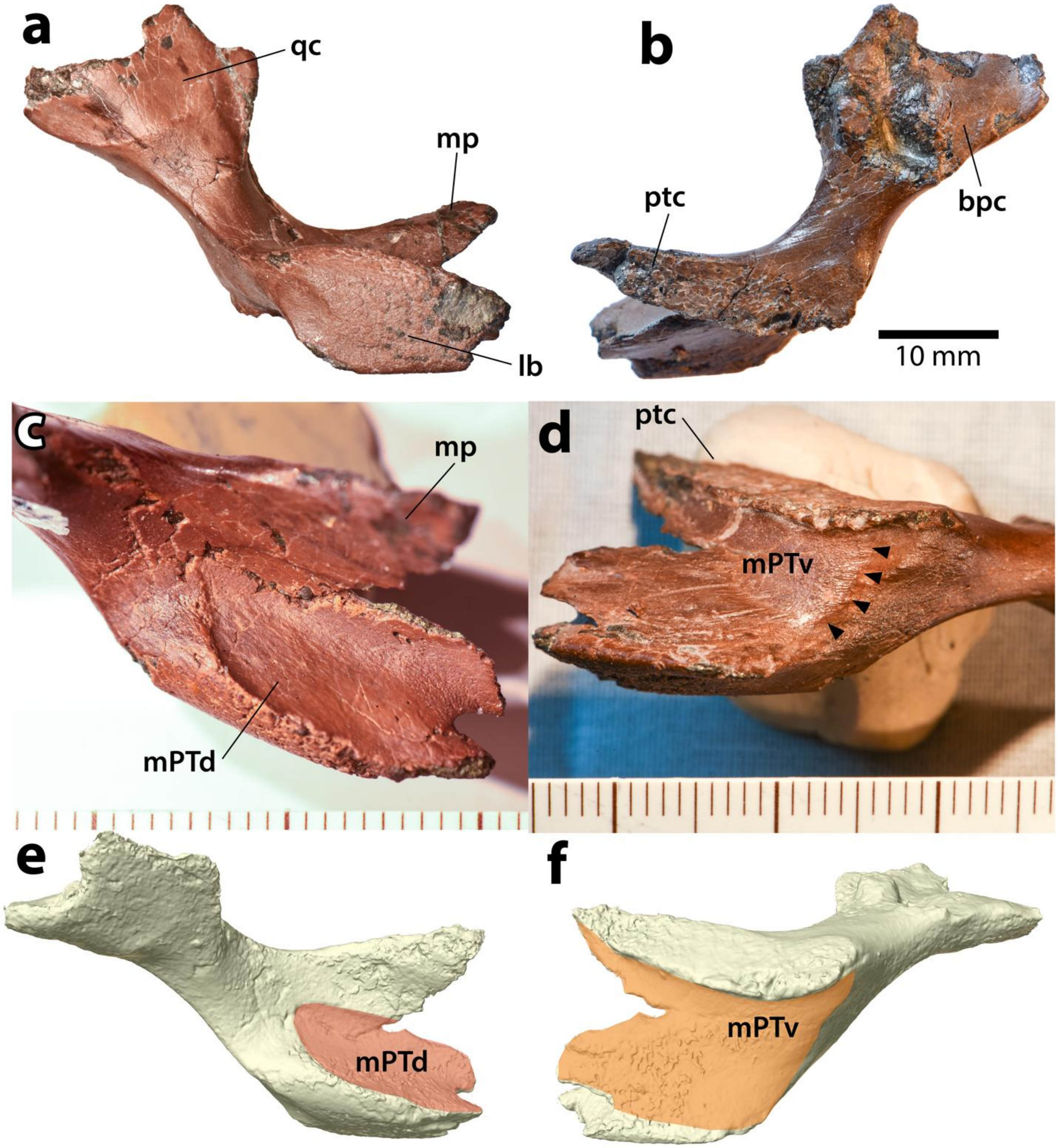

Supplementary Fig. 2. Pterygoid (ROM VP 88690; paratype) of *Infernogallus jamesi*. a–b, Right pterygoid of ROM VP 88690 in lateral (**a**) and medial (**b**) views. **c–d**, photographs of muscular attachment areas for *pterygoideus ventralis* (**c**) in dorsolateral view and *pterygoideus dorsalis* (**d**) in ventromedial view, showing textural changes marking edges of muscular attachment (arrows). **e–f**, photogrammetric models of right pterygoid showing reconstructed muscular attachment areas, in dorsolateral (**e**) and ventromedial (**f**) views; note that these views do not exactly correspond to the perspectives in (**c**) and (**d**). Scale increments in (**c**) and (**d**) are millimeters, scale bar for (**a**) and (**b**) are as indicated.

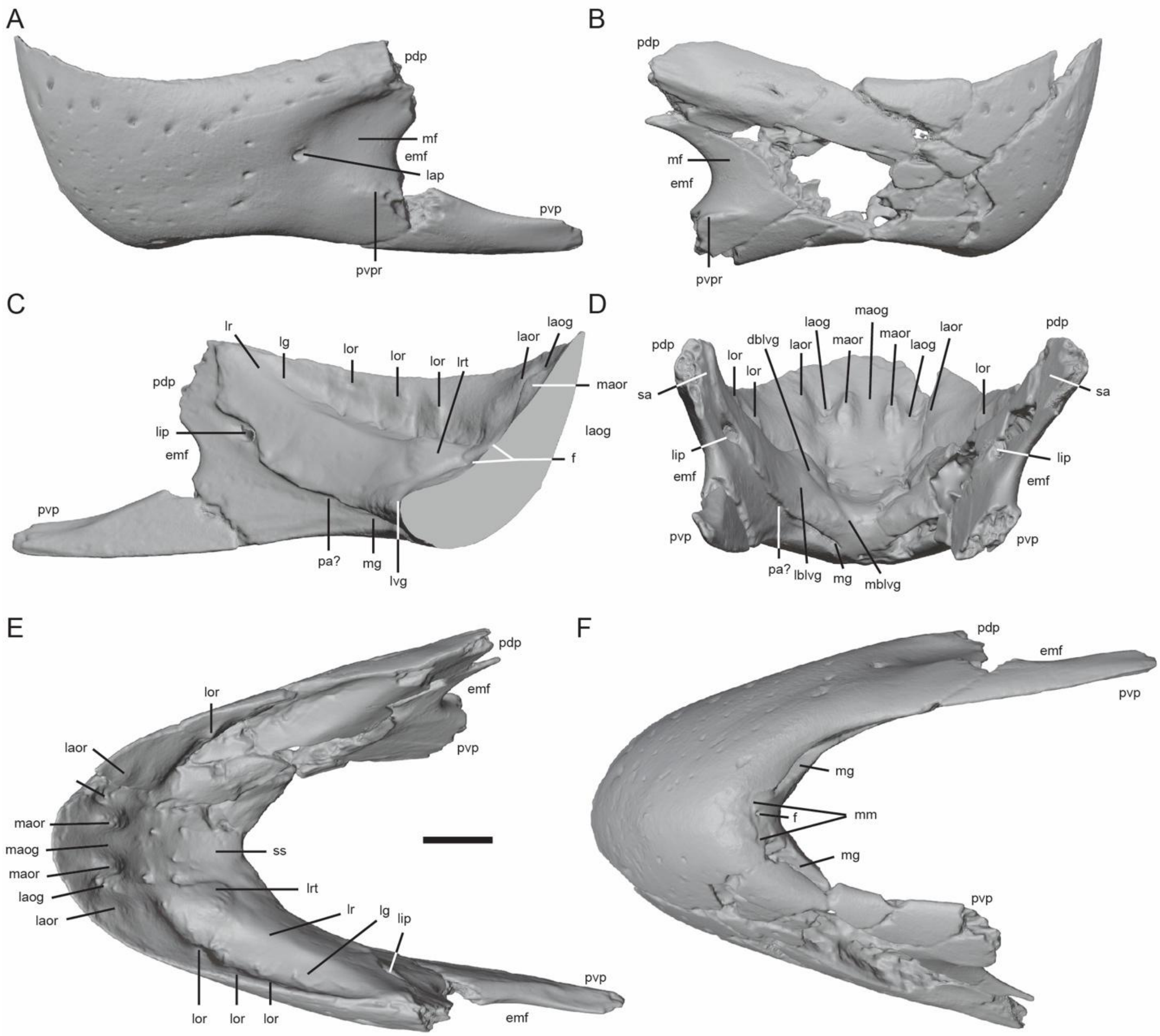

Supplementary Fig. 3. Paratype dentaries of *Infernogallus jamesi*. Three-dimensional model of NDGS 4351 in left lateral (A), right lateral (B), bisected medial (C), posterior (D), dorsal (E), and ventral (F) views. **Abbreviations: dblvg**, dorsal branch of the longitudinal vascular groove; **emf**, external mandibular fenestra; **f**, foramen; **loag**, labial anterior occlusal groove; **laor**, labial anterior occlusal ridge; **lap**, lateral pneumatopore; **lblvg**, lateral branch of the longitudinal vascular groove; **lip**, lingual pneumatopore; **lg**, lingual groove; **lor**, lateral occlusal ridge; **lr**, lingual ridge; **lrt**, lingual ridge tubercle; **lvg**, longitudinal vascular groove; **maog**, midline anterior occlusal groove; **maor**, medial anterior occlusal ridge; **mblvg**, medial branch of the longitudinal vascular groove; **mf**, mandibular fossa; **mg**, Meckelian groove; **mm**, depression for the M. genioglossus; **pa?,** possible articulation facet for the prearticular; **pdp**, posterodorsal process; **pvp**, posteroventral process; **pvpr**, posteroventral process ridge; **sa**, articulation surface for the ASC complex; **ss**, symphyseal shelf. Scale bar is 1 cm.

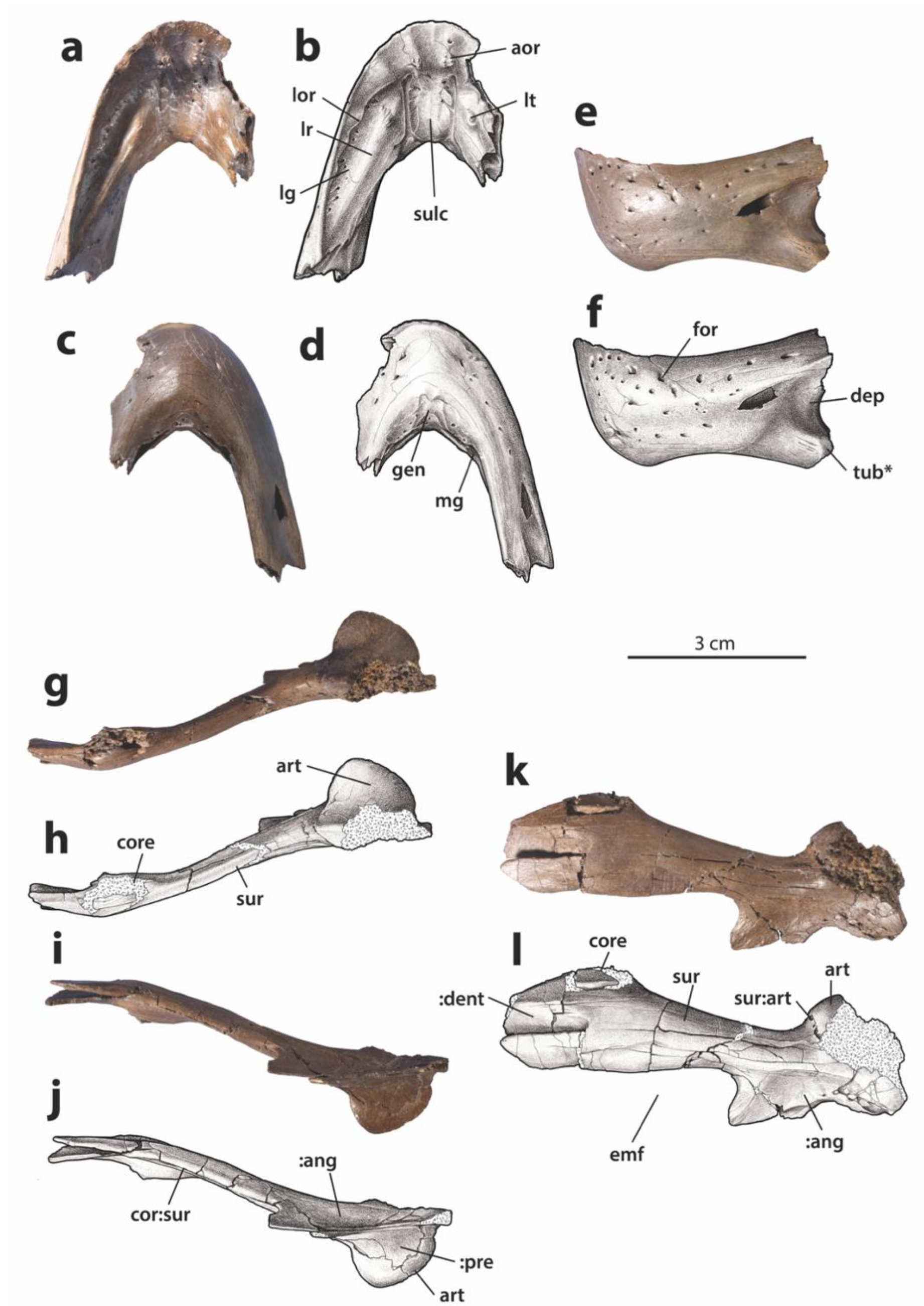

Supplementary Fig. 4. Paratype mandibles of *Infernogallus jamesi*. Photographs (**a, c, e, g, i, k**) and illustrations (**b, d, f, h, j, l**) of ROM VP 64193, fused left and right dentaries (**a–f**) and associated left articular-surangular-coronoid complex (ROM VP 64194; **g–l**), in dorsal (**a, b, g, h**), ventral (**c, d, i, j**), and left lateral (**e, f, k, l**) views. **Abbreviations: ang,** angular; **:ang**, contact for articular; **aor**, anterior occlusal ridge; **art**, articular; **core**, coronoid eminence; **cor:sur** suture between coronoid and surangular; **:dent,** contact for dentary; **dep**, depression; **emf**, external mandibular fenestra; **for**, foramen; **gen**, geniogloussus attachment; **lg**, lingual groove; **lor**, lateral occlusal ridge; **lr**, lingual ridge; **lt**, lingual tubercle; **mg**, Meckelian groove; **:pre**, contact for prearticular; **sulc**, midline sulcus; **sur**, surangular; **sur:art**, suture between surangular and articular; **tub***, autapomorphic tubercle on the posteroventral process of the dentary.

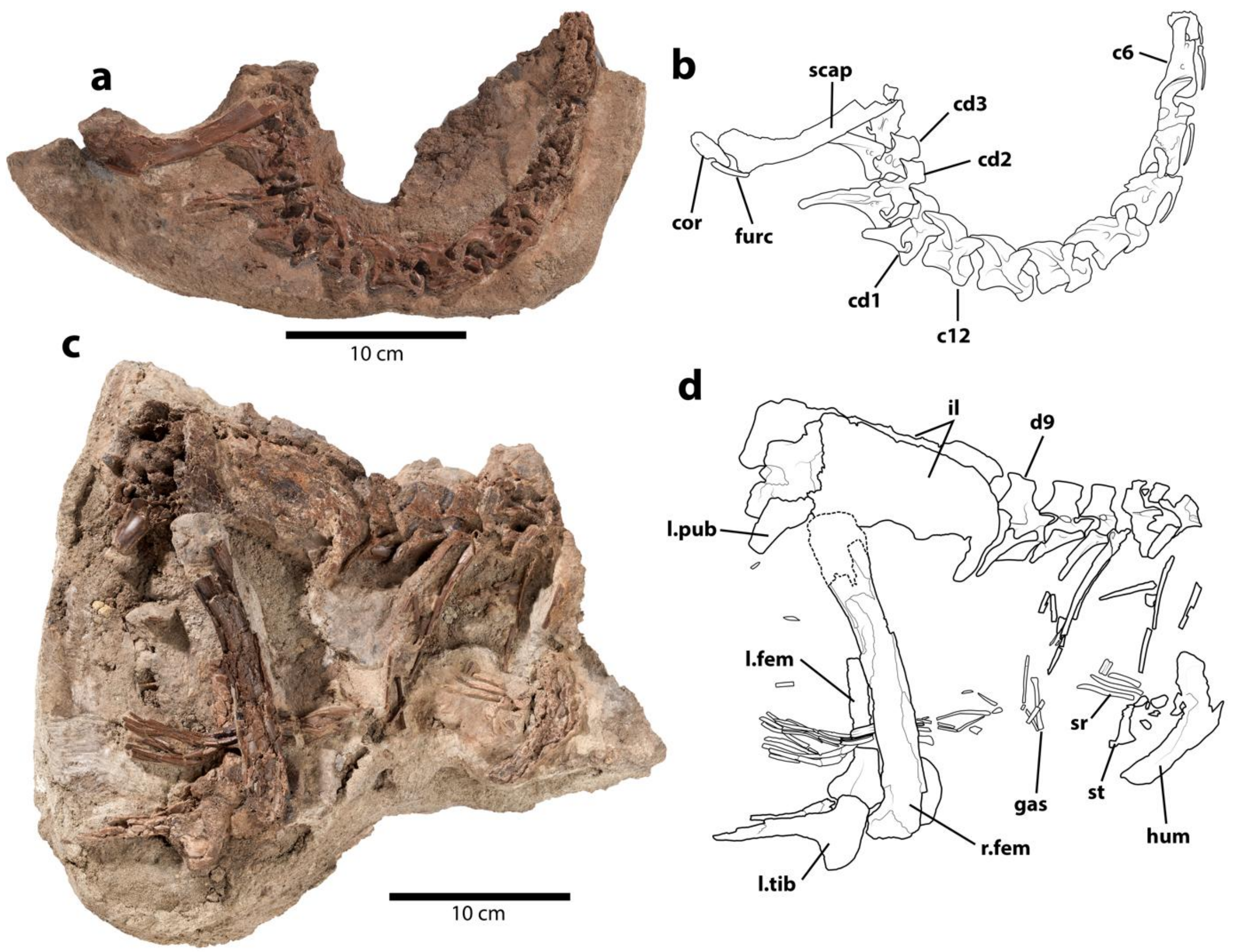

Supplementary Fig. 5. Neck and body blocks of holotype of *Infernogallus jamesi*. Photograph (**a**) and line drawing (**b**) of neck block of ROM VP 65050, extending from cervical vertebra 5 to the scapulacoracoid. Photograph (c) and line drawing (d) of body block, extending from dorsal vertebra 5 to the acetabulum of the pelvis. **Abbreviations: cor**, coracoid; **c6**, cervical vertebra 6; **c12**, cervical vertebra 12; **cd1–3**, cervicodorsal vertebrae 1 to 3; **d9**, dorsal vertebra 9; **furc**, furcula; **gas**, gastralia; **hum**, humerus; **il**, ilia; **l.fem**, left femur; **l.pub**, left pubis; **l.tib**, left tibia; **r.fem**, right femur; **sr**, sternal ribs; **st**, sternal plate; **scap**, scapula. Scale bar as indicated.

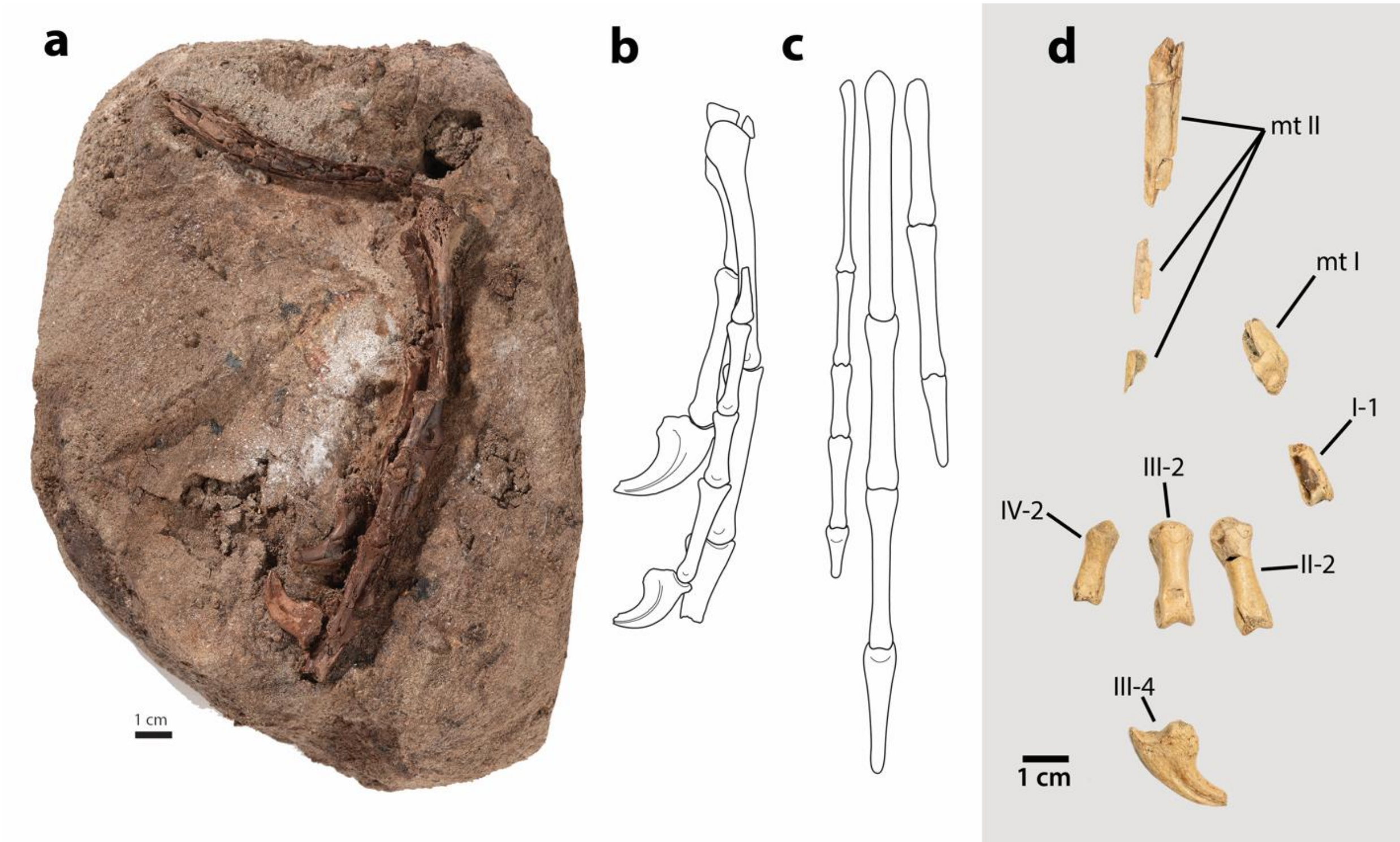

Supplementary Fig. 6. Hand and foot of holotype of *Infernogallus jamesi*. Photograph (**a**), line drawing (**b**), and reconstruction (**c**) of the right manus of ROM VP 65050 in lateral view (**a, b**) and in extensor view (**c**). Photograph (d) of the surface-collected pedal elements of ROM VP 65050 in extensor view. **Abbreviations: I-1**, phalanx I-1; **II-2**, phalanx II-2; **III-2**, phalanx III-2; **III-4**, phalanx III-4; **IV-2**, phalanx IV-2; **mt I**, metatarsal I; **mt II**, metatarsal II.

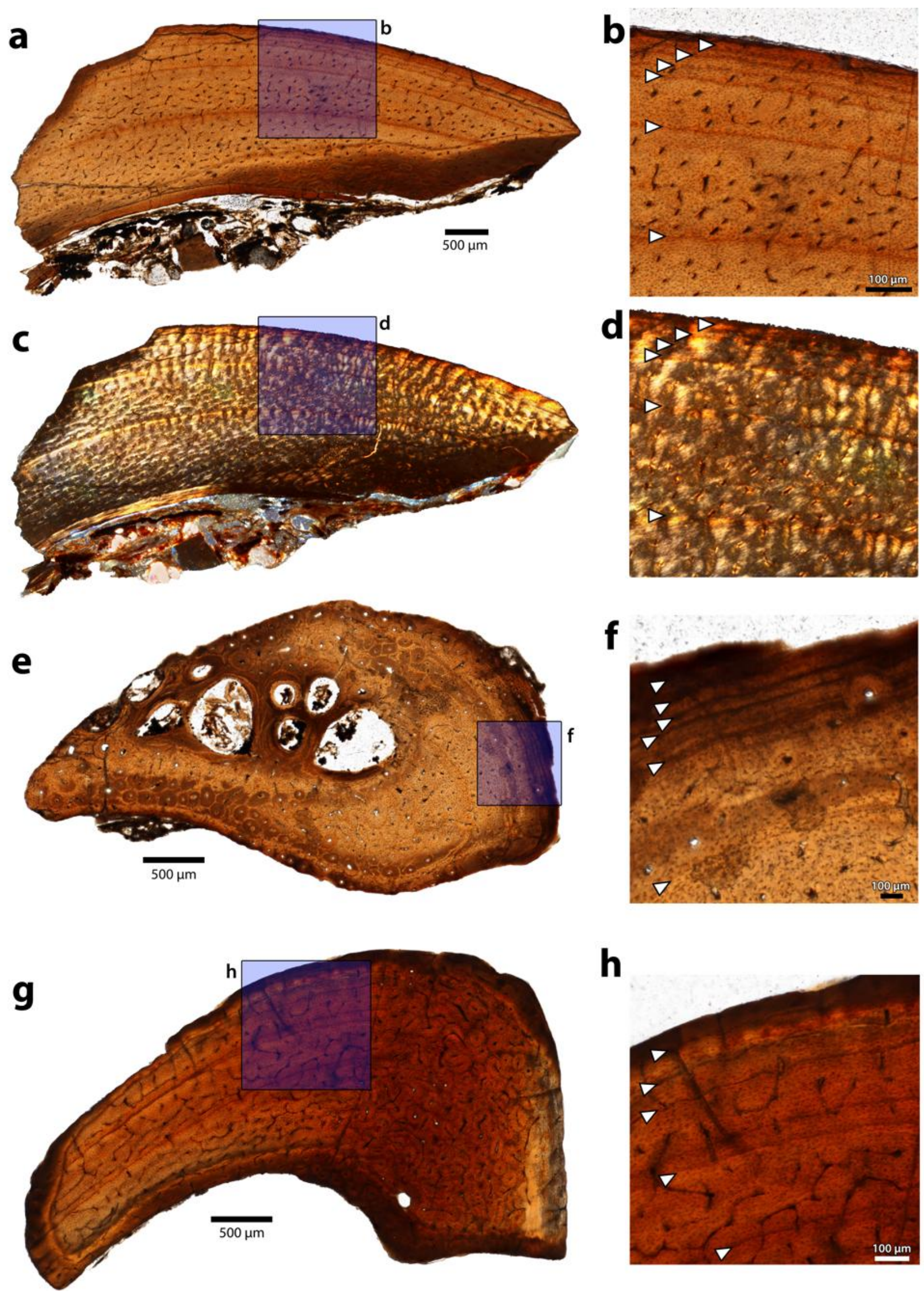

Supplementary Fig. 7. Palaeohistology of the holotype of *Infernogallus jamesi*.
Palaeohistological thin sections of the femur (**a–d**), fibula (**e, f**) and metatarsal II (**g, h**) of ROM VP 65050 in plane-polarized light (**a,b, e–h**) and cross-polarized light (**c, d**). Blue boxes in (**a, c, e, g**) show the locations of insets (**b, d, f, h**). Close-ups (**b, d, f, h**) show the external cortical region, with lines of arrested growth (arrows) becoming closely packed towards the periosteal surface and increasing organization of bone matrix fibers, indicative of reduced annual growth rates and skeletal maturity.

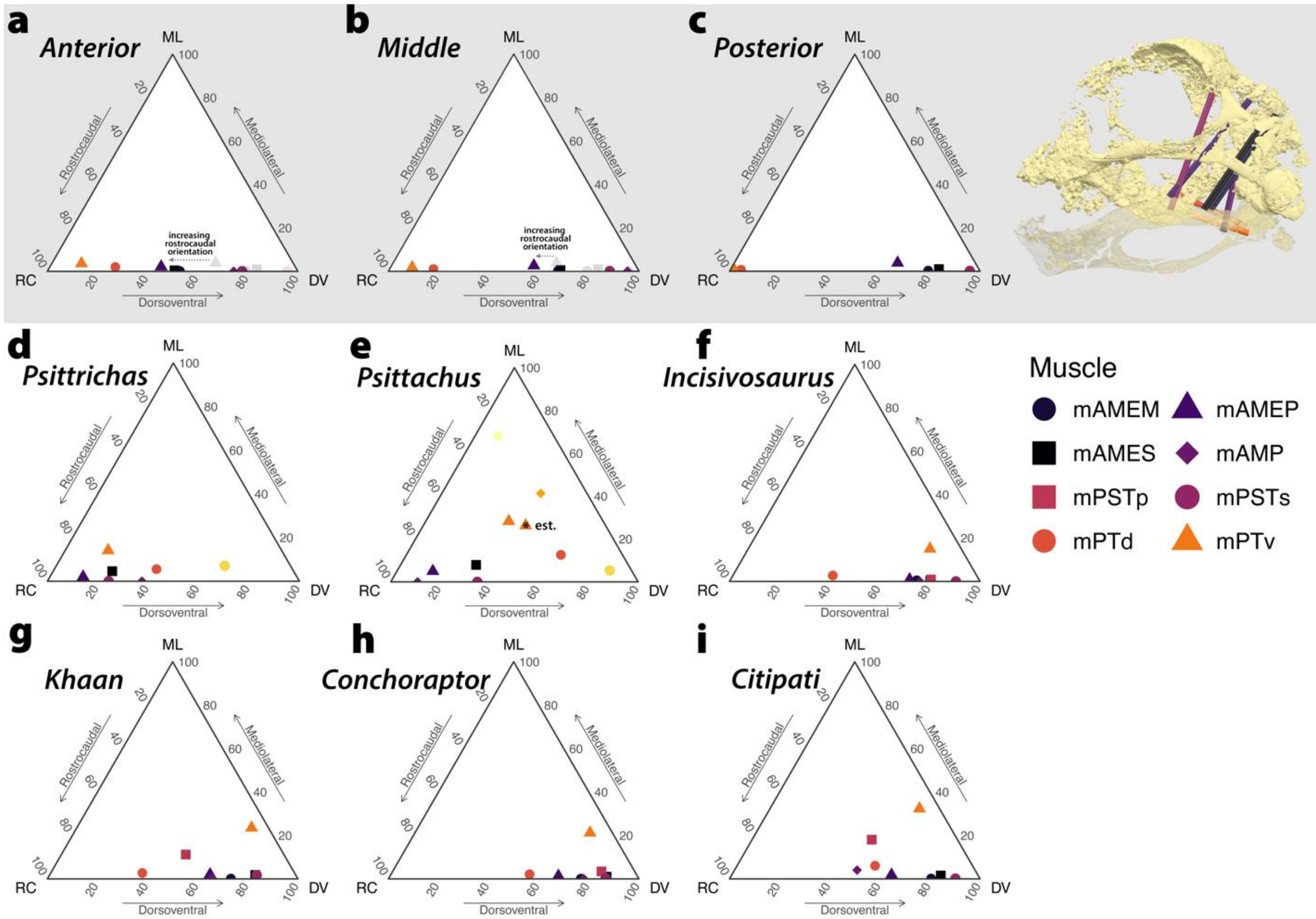

Supplementary Fig. 8. Muscle force vectors of *Infernogallus jamesi* and key comparison taxa. Top row (**a**–**c**) shows ternary plots for three jaw position arrangements for *Infernogallus*, at the anterior (**a**), middle (**b**), and posterior (**c**) of the articular range of the mandible and quadrate. **d**–**i**, comparison taxa, in order of similarity: *Psittrichas* (**d**), *Psittachus* (**e**), *Incisivosaurus* (**f**), *Khaan* (**g**), *Conchoraptor* (**h**), and *Citipati* (**i**). Note in (**e**), the marked reconstruction for mPTv was estimated using the same method as in *Infernogallus*.

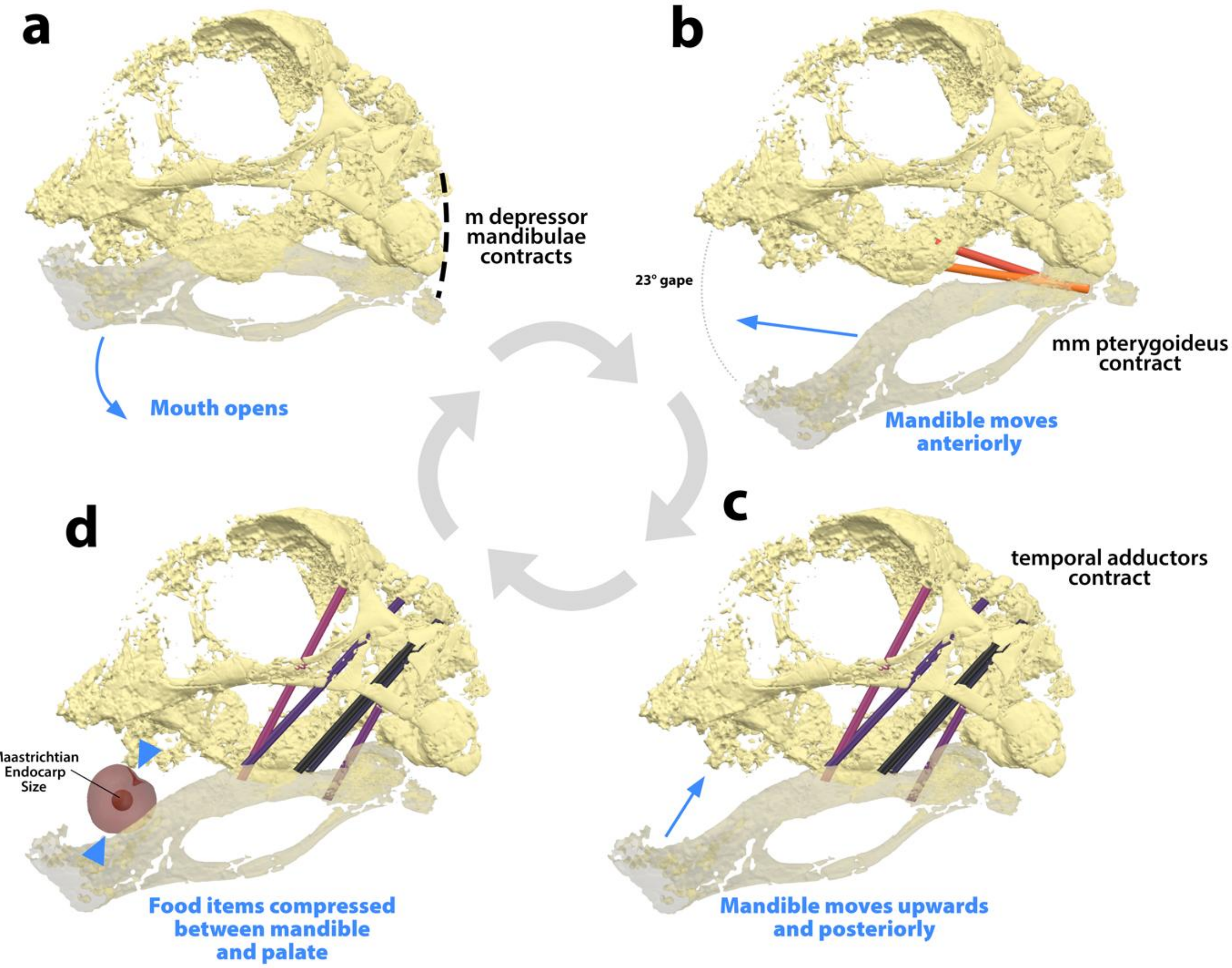

Supplementary Fig. 9. Reconstruction of anteroposterior movement of the mandible during the chewing stroke in *Infernogallus jamesi*. Three-dimensional models of the skull and mandible showing potential mandible positions based on contact of the articular and quadrate, and resulting adductor musculature orientations. From rest (**a**), contraction of the m. depressor mandibulae would open the mouth (**b**). Contraction of the the mm. pterygoideus in this position would move the mandible forwards (**c**). This anterior movement would increase the anteroposterior orientations of the temporal adductor musculature (see also Supplementary Fig. 7a–c). Contraction of the temporal adductor muscles in this position would bring the mandible upwards and backwards (**c**). This movement would enable manipulation and compression of foods between the triturating features of the dentary and the maxillovomeral process of the palate (**d**). Blue arrows and text indicate inferred motions, black text indicates muscle groups contracting. M depressor mandibulae could not be reconstructed based on the areas exposed in ROM VP 65050, but it has deeply conserved attachments on the posterior process of the squamosal and retroarticular process of the mandible in archosaurs [5]. In positions (**c, d**), the m depressor mandibulae would rest medial to the quadratojugal.

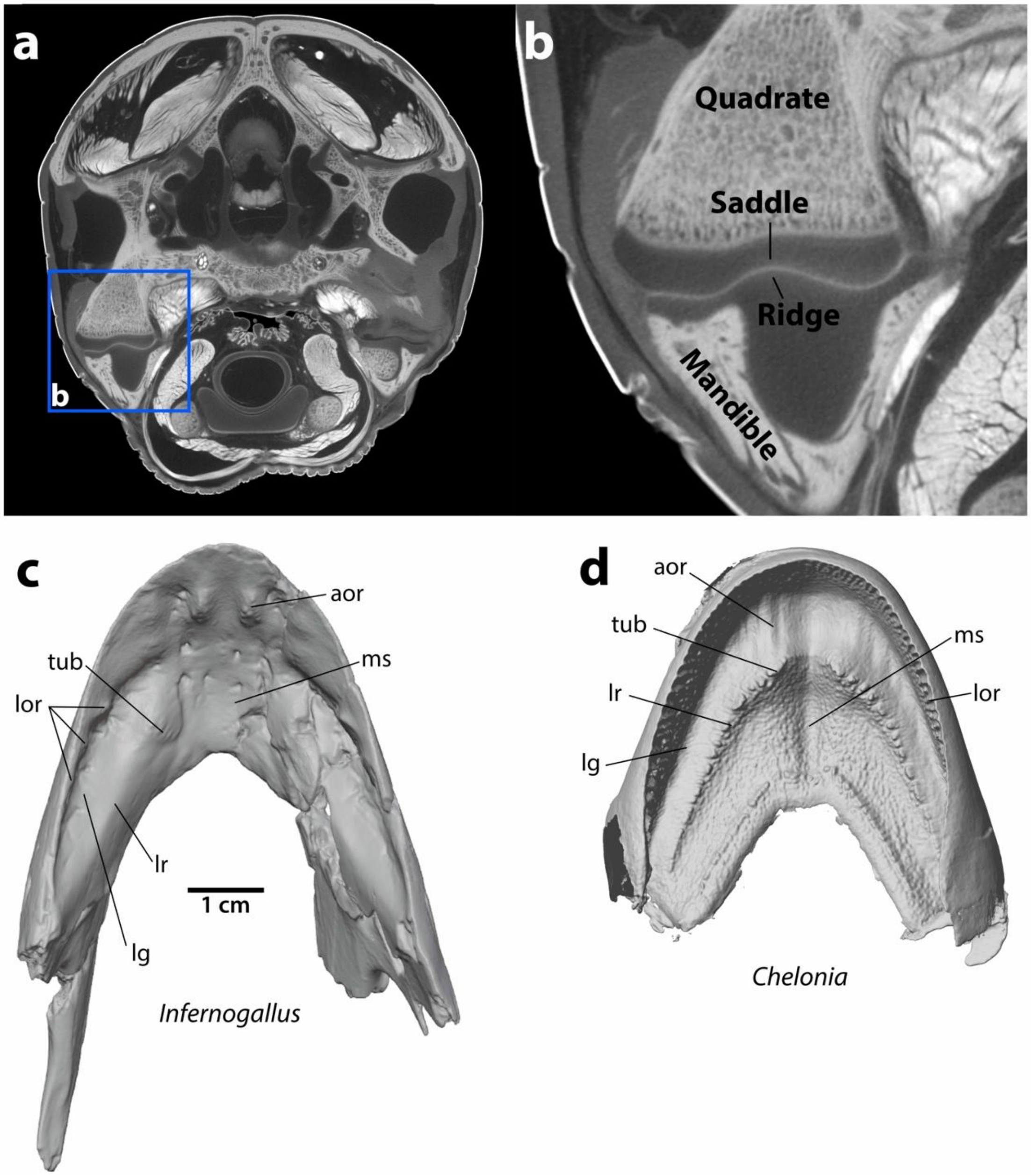

Supplementary Fig. 10. Morphological similarities between eucaenagnathines and extant soft-plant eating turtles. **A,b,** DiceCT stacked image of the mandibular joint of *Chelonia mydas*, showing a ridge-and-saddle configuration formed by soft tissues. Blue box in (**a**) shows the location of detail in (**b**). **c,d,** comparison of eucaenagnathine dentaries with turtle maxillae. NDGS 4351, fused left and partial right dentaries (paratype) in dorsal view (**c**), showing typical arrangement of eucaenagnathine dentaries, with prominent lingual ridges (lr) and a midline sulcus (ms). Keratin sheath (**d**) of the maxilla of ROM R3700 (*Chelonia mydas*) in ventral view, showing similar arrangement, with triturating ridges bordering a midline sulcus. Images **a,b** courtesy of the Florida Museum of Natural History. **Abbreviations: aor**, anterior occlusal ridge; **lg**, lingual groove; **lor**, lateral occlusal ridge; **lr**, lingual ridge; **ms**, midline sulcus; **R**, ridge; **S**, saddle; **tub**, tubercle of the lingual ridge.

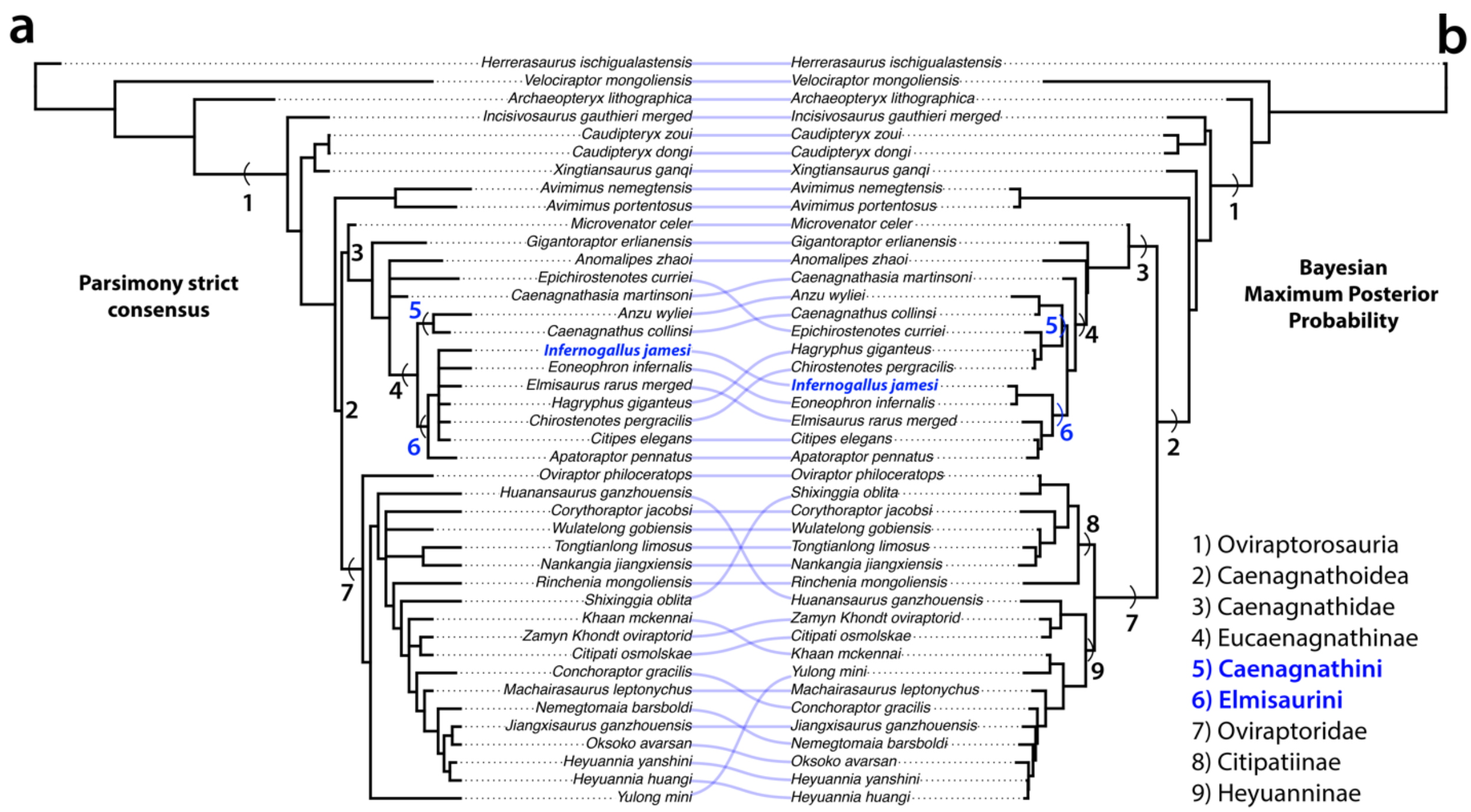

Supplementary Fig. 11. Topological differences between parsimony and Bayesian phylogenetic analyses. Strict consensus of the parsimony analysis (**a**) and the maximum posterior probability tree of the full-matrix, non-clock Bayesian analysis (**b**), with taxa linked on both trees to show changes in relationships. Note inconsistent constituency of proposed subclades 'Elmisaurini' and 'Caenagnathini', cautioning against formal establishment of these clades.

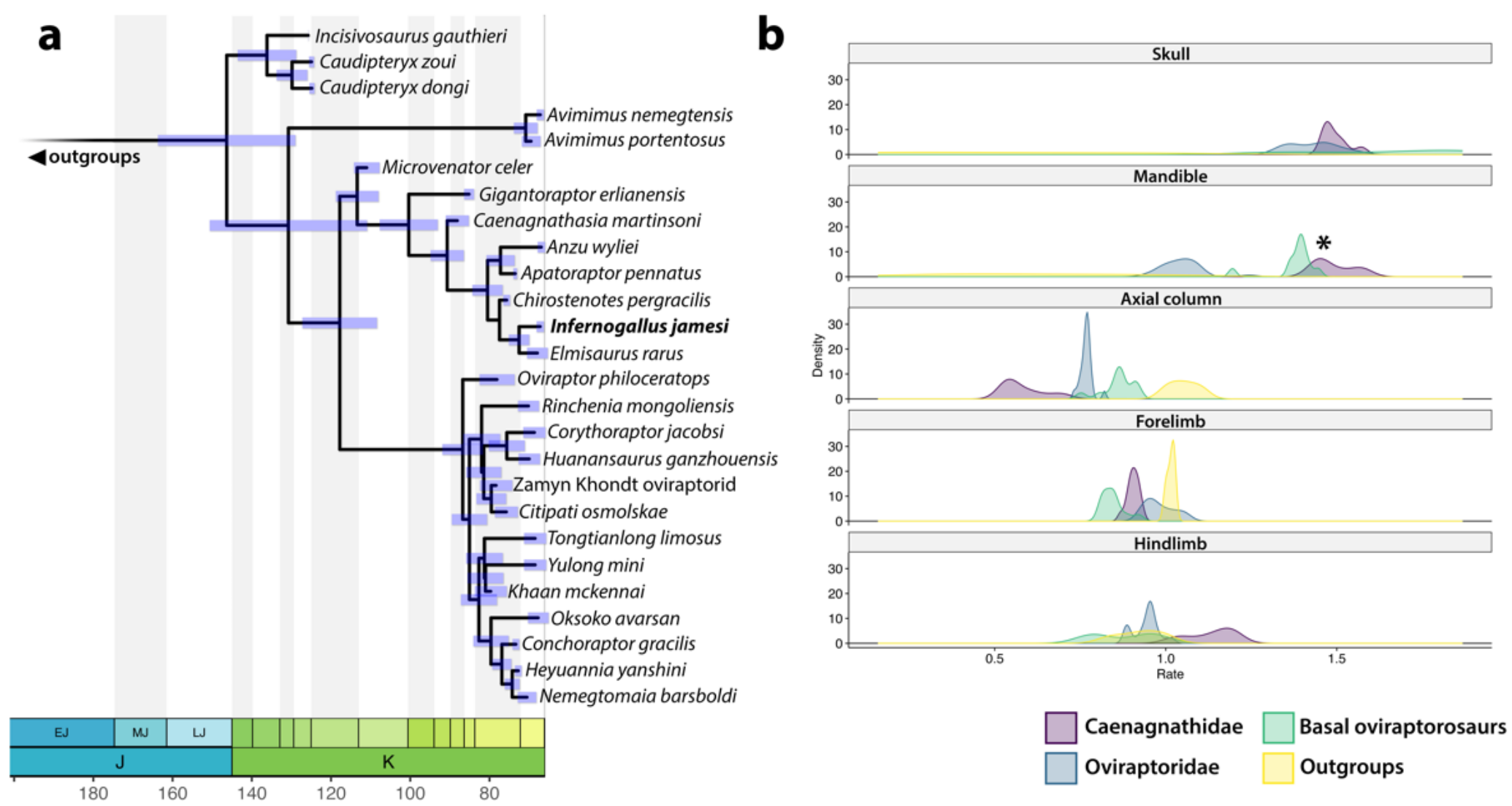

Supplementary Fig. 12. Skyline FBD relaxed morphological clock analysis of Oviraptorosauria. Maximum posterior probability tree (**a**), showing preferred topology (identical to Maximum Clade Credibility tree). Blue bars indicate 95% confidence intervals in age for each node. Estimated clock rates for each morphological partition (**b**) show that caenagnathids have elevated rates of mandibular evolution (asterisk) relative to oviraptorids and other groups of oviraptorosaurs.

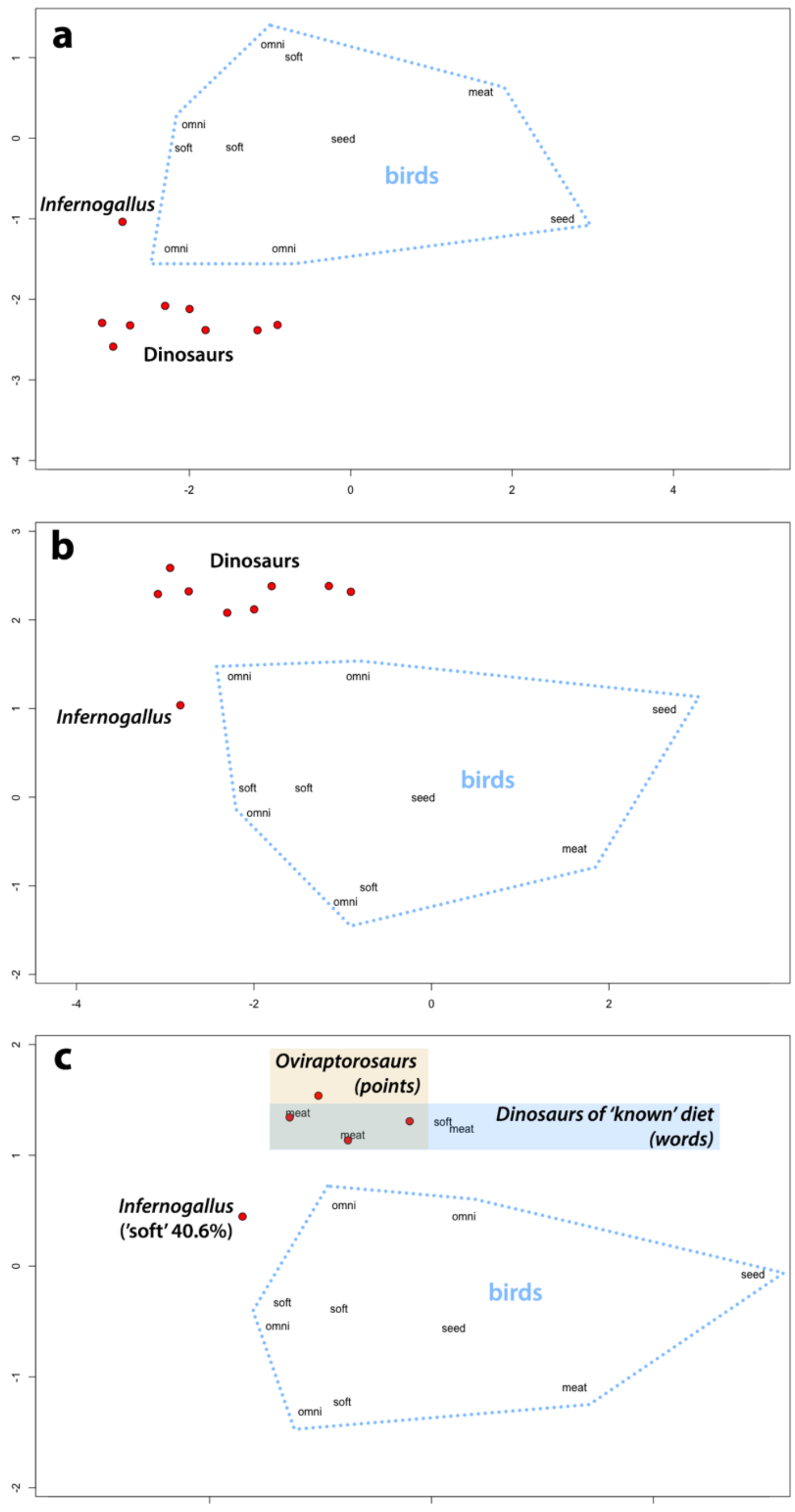

Supplementary Fig. 13. Statistical analysis of diet based on mandibular adductor muscle vectors in birds and non-avian theropods. Non-phylogenetically-corrected linear discriminant analysis (**a**), showing distinct regions of space for dinosaurs and birds, with the exception of *Infernogallus*. LDA with phylogenetic correction (**b**) *via* phylogenetic principal components analysis (see Supplementary Discussion), showing nearly identical results to (**a**) with inverted axes. Exploratory LDA with phylogenetic correct (**c**) incorporating dietary evidence for non-avian theropods except oviraptorosaurs (see Supplementary Discussion), showing differing arrangement of points and prediction of a soft plant diet for *Infernogallus*. Note that blue hulls are not statistical convex hulls, they are illustrative only.

References:

1. Herring, S. W. Mechanical Influences on Suture Development and Patency. in *Frontiers of Oral Biology* (ed. Rice, D. P.) vol. 12 41–56 (S. KARGER AG, Basel, 2008).
2. Clark, J. M., Norell, M. A. & Rowe, T. Cranial anatomy of *Citipati osmolskae* (Theropoda, Oviraptorosauria), and a reinterpretation of the holotype of *Oviraptor philoceratops*. *American Museum Novitates* 1–24 (2002).
3. Osmólska, H., Currie, P. J. & Barsbold, R. Oviraptorosauria. in *The Dinosauria* (eds Weishampel, D. B., Dodson, P. & Osmólska, H.) 165–183 (University of California Press, Berkeley, California, 2004).
4. Osmólska, H. New light on the skull anatomy and systematic position of *Oviraptor*. *Nature* **262**, 683–684 (1976).
5. Holliday, C. M. New Insights Into Dinosaur Jaw Muscle Anatomy. *The Anatomical Record: Advances in Integrative Anatomy and Evolutionary Biology* **292**, 1246–1265 (2009).
6. Tsai, H. P. & Holliday, C. M. Ontogeny of the Alligator Cartilago Transiliens and Its Significance for Sauropsid Jaw Muscle Evolution. *PLoS ONE* **6**, e24935 (2011).
7. Balanoff, A. M., Xu, X., Kobayashi, Y., Matsufune, Y. & Norell, M. A. Cranial Osteology of the Theropod Dinosaur *Incisivosaurus gauthieri* (Theropoda: Oviraptorosauria). *American Museum Novitates* **3651**, 1–35 (2009).
8. Funston, G. F., Mendonca, S. E., Currie, P. J. & Barsbold, R. Oviraptorosaur anatomy, diversity and ecology in the Nemegt Basin. *Palaeogeography, Palaeoclimatology, Palaeoecology* **494**, 101–120 (2018).
9. Funston, G. F. Osteology of the two-fingered oviraptorid *Oksoko avarsan* (Theropoda: Oviraptorosauria). *Zoological Journal of the Linnean Society* zlae011 (2024) doi:10.1093/zoolinnean/zlae011.
10. Sues, H.-D. On *Chirostenotes*, a Late Cretaceous oviraptorosaur (Dinosauria: Theropoda) from western North America. *Journal of Vertebrate Paleontology* **17**, 698–716 (1997).
11. Funston, G. F. Caenagnathids of the Dinosaur Park Formation (Campanian) of Alberta, Canada: anatomy, osteohistology, taxonomy, and evolution. *Vert. Anat. Morph. Palaeo.* **8**, 105–153 (2020).
12. Funston, G. F. & Currie, P. J. A new caenagnathid (Dinosauria: Oviraptorosauria) from the Horseshoe Canyon Formation of Alberta, Canada, and a reevaluation of the relationships of Caenagnathidae. *Journal of Vertebrate Paleontology* **36**, e1160910 (2016).
13. Funston, G. F., Currie, P. J. & Burns, M. New elmisaurine specimens from North America and their relationship to the Mongolian *Elmisaurus rarus*. *Acta Palaeontologica Polonica* **61**, 159–173 (2016).
14. Porro, L. B. *et al.* Free body analysis, beam mechanics, and finite element modeling of the mandible of *Alligator mississippiensis*. *Journal of Morphology* **272**, 910–937 (2011).
15. Iordansky, N. N. & Gans, C. The skull of the Crocodilia. *Biology of the Reptilia* **4**, 201–262 (1973).
16. Tsai, H. & Holliday, C. Articular soft tissue anatomy of the archosaur hip joint: Structural homology and functional implications. *Journal of Morphology* **276**, (2014).
17. Lamanna, M. C., Sues, H.-D., Schachner, E. R. & Lyson, T. R. A new large-bodied oviraptorosaurian theropod dinosaur from the latest Cretaceous of Western North America. *PLoS ONE* **9**, e92022 (2014).

18. Funston, G. F. *et al.* A new two-fingered dinosaur sheds light on the radiation of Oviraptorosauria. *R. Soc. open sci.* **7**, 201184 (2020).
19. Wei, X. *et al.* A new subadult specimen of oviraptorid *Yulong mini* (Theropoda: Oviraptorosauria) from the Upper Cretaceous Qiupa Formation of Luanchuan, central China. *Cretaceous Research* **138**, 105261 (2022).
20. Longrich, N. R., Barnes, K., Clark, S. & Millar, L. Caenagnathidae from the Upper Campanian Aguja Formation of West Texas, and a revision of the Caenagnathinae. *Bulletin of the Peabody Museum of Natural History* **54**, 23–49 (2013).
21. Zanno, L. E. & Sampson, S. D. A new oviraptorosaur (Theropoda, Maniraptora) from the Late Cretaceous (Campanian) of Utah. *Journal of Vertebrate Paleontology* **25**, 897–904 (2005).
22. Gilmore, C. W. A new coelurid dinosaur from the Belly River Cretaceous of Alberta. *Canada Geological Survey Bulletin* **38**, 1–12 (1924).
23. Currie, P. J. & Russell, D. A. Osteology and relationships of *Chirostenotes pergracilis* (Saurischia, Theropoda) from the Judith River (Oldman) Formation of Alberta, Canada. *Canadian Journal of Earth Sciences* **25**, 972–986 (1988).
24. Currie, P. J., Godfrey, S. J. & Nessov, L. New caenagnathid (Dinosauria: Theropoda) specimens from the Upper Cretaceous of North America and Asia. *Canadian Journal of Earth Sciences* **30**, 2255–2272 (1993).
25. Sternberg, R. M. A toothless bird from the Cretaceous of Alberta. *Journal of Paleontology* **14**, 81–85 (1940).
26. Currie, P. J. The first records of *Elmisaurus* (Saurischia, Theropoda) from North America. *Canadian Journal of Earth Sciences* **26**, 1319–1324 (1989).
27. Currie, P. J., Funston, G. F. & Osmolska, H. New specimens of the crested theropod dinosaur *Elmisaurus rarus* from Mongolia. *Acta Palaeontologica Polonica* **61**, 143–157 (2016).
28. Osmólska, H. Coossified tarsometatarsi in theropod dinosaurs and their bearing on the problem of bird origins. *Palaeontologia Polonica* **42**, 79–95 (1981).
29. Funston, G. F. & Currie, P. J. A previously undescribed caenagnathid mandible from the late Campanian of Alberta, and insights into the diet of *Chirostenotes pergracilis* (Dinosauria: Oviraptorosauria). *Canadian Journal of Earth Sciences* **51**, 156–165 (2014).
30. Shipps, B. K., Peecook, B. R. & Angielczyk, K. D. The topography of diet: Orientation patch count predicts diet in turtles. *The Anatomical Record* **306**, 1214–1227 (2023).
31. Lautenschlager, S., Witmer, L. M., Altangerel, P. & Rayfield, E. J. Edentulism, beaks, and biomechanical innovations in the evolution of theropod dinosaurs. *Proceedings of the National Academy of Sciences* **110**, 20657–20662 (2013).
32. Wang, S. *et al.* Heterochronic truncation of odontogenesis in theropod dinosaurs provides insight into the macroevolution of avian beaks. *Proceedings of the National Academy of Sciences* **114**, 10930–10935 (2017).
33. Marshall, C. D. *et al.* Scaling of bite performance with head and carapace morphometrics in green turtles (Chelonia mydas). *Journal of Experimental Marine Biology and Ecology* **451**, 91–97 (2014).
34. Froggatt, J. & Gill, B. Bill morphology reflects adaptation to a fibrous diet in the kākāpō (*Strigops* : Psittaciformes). *New Zealand Journal of Zoology* **43**, 138–148 (2016).
35. Verhulst, C. D. 3D Muscle Fiber Architecture of Avian Jaw Muscles and their Significance for Avian cranial functional morphology evolution. *The FASEB Journal* **34**, 1–1 (2020).

36. Angielczyk, K. D. Phylogenetic evidence for and implications of a dual origin of propaliny in anomodont therapsids (Synapsida). *Paleobiology* **30**, 268–296 (2004).
37. Jones, M. E. H. Skull shape and feeding strategy in Sphenodon and other Rhynchocephalia (Diapsida: Lepidosauria). *Journal of Morphology* **269**, 945–966 (2008).
38. Curtis, N. *et al.* Functional Relationship between Skull Form and Feeding Mechanics in Sphenodon, and Implications for Diapsid Skull Development. *PLoS ONE* **6**, e29804 (2011).
39. Schwarz, D., Konow, N., Roba, Y. T. & Heiss, E. A salamander that chews using complex, three-dimensional mandible movements. *Journal of Experimental Biology* jeb.220749 (2020) doi:10.1242/jeb.220749.
40. Krause, D. W. Jaw movement, dental function, and diet in the Paleocene multituberculate Ptilodus. *Paleobiology* **8**, 265–281 (1982).
41. Mao, F., Li, Z., Hooker, J. J. & Meng, J. A new euharamiyidan, *Mirusodens caii* (Mammalia: Euharamiyida), from the Jurassic Yanliao Biota and evolution of allotherian mammals. *Zoological Journal of the Linnean Society* **199**, 832–859 (2023).
42. Cracraft, J. Caenagnathiformes: Cretaceous birds convergent in jaw mechanism to dicynodont reptiles. *Journal of Paleontology* **45**, 805–809 (1971).
43. Jasinoski, S. C., Rayfield, E. J. & Chinsamy, A. Comparative Feeding Biomechanics of *Lystrosaurus* and the Generalized Dicynodont *Oudenodon*. *The Anatomical Record: Advances in Integrative Anatomy and Evolutionary Biology* **292**, 862–874 (2009).
44. King, G. M. The Functional Anatomy of a Permian Dicynodont. *Philosophical Transactions of the Royal Society B: Biological Sciences* **291**, 243–322 (1981).
45. King, G. M., Oelofsen, B. W. & Rubidge, B. S. The evolution of the dicynodont feeding system. *Zoological Journal of the Linnean Society* **96**, 185–211 (1989).
46. Iori, F. V. & Carvalho, I. de S. The Cretaceous crocodyliform *Caipirasuchus*: behavioral feeding mechanisms. *Cretaceous Research* **84**, 181–187 (2018).
47. Beecher, W. J. Adaptations for Food-Getting in the American Blackbirds. *The Auk* **68**, 411–440 (1951).
48. Zusi, R. L. The Role of the Depressor Mandibulae Muscle in Kinesis of the Avian Skull. *Proceedings of the United States National Museum* **123**, 1–28 (1967).
49. Mayr, G. A new eocene *Chascacocolius*-like mousebird (Aves: Coliiformes) with a remarkable gaping adaptation. *Organisms Diversity & Evolution* **5**, 167–171 (2005).
50. Mack, A. L. & Wright, D. D. The Vulturine Parrot, *Psittrichas fulgidus* , a threatened New Guinea endemic: notes on its biology and conservation. *Bird Conservation International* **8**, 185–194 (1998).
51. Carrano, M. T. *Amniote Paleobiology: Perspectives on the Evolution of Mammals, Birds, and Reptiles*. (University of Chicago Press, 2006).
52. Qiang, J., Currie, P. J., Norell, M. A. & Shu-An, J. Two feathered dinosaurs from northeastern China. *Nature* **393**, 753–761 (1998).
53. Rhodes, M. M., Funston, G. F. & Currie, P. J. New material reveals the pelvic morphology of Caenagnathidae (Theropoda, Oviraptorosauria). *Cretaceous Research* **114**, 104521 (2020).
54. Therrien, F. *et al.* Exceptionally preserved stomach contents of a young tyrannosaurid reveal an ontogenetic dietary shift in an iconic extinct predator. *Science Advances* **9**, eadi0505 (2023).

55. Coiffard, C., Gomez, B., Daviero-Gomez, V. & Dilcher, D. L. Rise to dominance of angiosperm pioneers in European Cretaceous environments. *Proc. Natl. Acad. Sci. U.S.A.* **109**, 20955–20959 (2012).
56. Xu, X. & Barrett, P. M. The origin and early evolution of feathers: implications, uncertainties and future prospects. *Biol. Lett.* **21**, 20240517 (2025).
57. Xu, X., Zheng, X. & You, H. Exceptional dinosaur fossils show ontogenetic development of early feathers. *Nature* **464**, 1338–1341 (2010).
58. Brush, A. H. Metabolism of carotenoid pigments in birds. *The FASEB Journal* **4**, 2969–2977 (1990).
59. Xu, X., Cheng, Y.-N., Wang, X.-L. & Chang, C.-H. An unusual oviraptorosaurian dinosaur from China. *Nature* **419**, 291–293 (2002).
60. Funston, G. F. Anatomy, Systematics, and Evolution of Oviraptorosauria (Dinosauria, Theropoda). (University of Alberta, Edmonton, Alberta, 2019).
61. Balanoff, A. M. & Norell, M. A. Osteology of *Khaan mckennai* (Oviraptorosauria: Theropoda). *Bulletin of the American Museum of Natural History* **372**, 1–77 (2012).
62. Tsuihiji, T. *et al.* New information on the cranial morphology of *Avimimus* (Theropoda: Oviraptorosauria). *Journal of Vertebrate Paleontology* e1347177 (2017) doi:10.1080/02724634.2017.1347177.
63. Funston, G. F. *et al.* The first oviraptorosaur (Dinosauria: Theropoda) bonebed: evidence of gregarious behaviour in a maniraptoran theropod. *Scientific Reports* **6**, 35782 (2016).
64. Kurzanov, S. M. Avimimidae and the problem of the origin of birds. *Transactions of the Joint Soviet Mongolian Paleontological Expedition* **31**, 1–92 (1987).
65. Meade, L. E. & Ma, W. Cranial muscle reconstructions quantify adaptation for high bite forces in Oviraptorosauria. *Sci Rep* **12**, 3010 (2022).
66. Meade, L. E., Pittman, M., Balanoff, A. & Lautenschlager, S. Cranial functional specialisation for strength precedes morphological evolution in Oviraptorosauria. *Commun Biol* **7**, 1–10 (2024).
67. Ma, W., Pittman, M., Lautenschlager, S., Meade, L. E. & Xu, X. Chapter 8 Functional Morphology of the Oviraptorosaurian and Scansoriopterygid Skull. *Bulletin of the American Museum of Natural History* 229–250 (2020).
68. Mulvaney, M. & Booksmythe, I. Gang-gang Cockatoo diet as assessed by camera images and written records. (2023).
69. Homberger, D. The comparative biomechanics of a prey-predator relationship: The adaptive morphologies of the feeding apparatus of Australian black-cockatoos and their foods as a basis for the reconstruction of the evolutionary history of the Psittaciformes. *Vertebrate Biomechanics and Evolution* 203–228 (2003).
70. Renton, K. Diet of Adult and Nestling Scarlet Macaws in Southwest Belize, Central America [1]. *Biotropica* **38**, 280–283 (2006).
71. Naware, D. & Benson, R. Patterns of variation in fleshy diaspore size and abundance from Late Triassic-Oligocene. *Biol Rev Camb Philos Soc* **99**, 430–457 (2024).
72. Xu, X. *et al.* A gigantic feathered dinosaur from the Lower Cretaceous of China. *Nature* **484**, 92–95 (2012).
73. Tsuihiji, T., Watabe, M., Barsbold, R. & Tsogtbaatar, K. A gigantic caenagnathid oviraptorosaurian (Dinosauria: Theropoda) from the Upper Cretaceous of the Gobi Desert, Mongolia. *Cretaceous Research* **56**, 60–65 (2015).

74. Funston, G. F., Currie, P. J., Ryan, M. J. & Dong, Z.-M. Birdlike growth and mixed-age flocks in avimimids (Theropoda, Oviraptorosauria). *Sci Rep* **9**, 18816 (2019).
75. Averianov, A. O. & Lopatin, A. V. First Discovery of Theropod Dinosaurs of the Family Avimimidae in the Late Cretaceous of Central Asia. *Dokl. Earth Sc.* **506**, 775–777 (2022).
76. Guo, Z.-X. *et al.* Inversion of the Erlian Basin (NE China) in the early Late Cretaceous: Implications for the collision of the Okhotomorsk Block with East Asia. *Journal of Asian Earth Sciences* **154**, 49–66 (2018).
77. Van Itterbeeck, J., Horne, D. J., Bultynck, P. & Vandenberghe, N. Stratigraphy and palaeoenvironment of the dinosaur-bearing Upper Cretaceous Iren Dabasu Formation, Inner Mongolia, People's Republic of China. *Cretaceous Research* **26**, 699–725 (2005).
78. Bonnetti, C. *et al.* Sedimentology, stratigraphy and palynological occurrences of the late Cretaceous Erlian Formation, Erlian Basin, Inner Mongolia, People's Republic of China. *Cretaceous Research* **48**, 177–192 (2014).
79. Makovicky, P. J. & Sues, H.-D. Anatomy and phylogenetic relationships of the theropod dinosaur *Microvenator celer* from the Lower Cretaceous of Montana. *American Museum Novitates* **3240**, 1–27 (1998).
80. Yao, X. *et al. Caenagnathasia* sp. (Theropoda: Oviraptorosauria) from the Iren Dabasu Formation (Upper Cretaceous: Campanian) of Erenhot, Nei Mongol, China. *Vertebrata PalAsiatica* **53**, 291–298 (2015).
81. Sues, H.-D. & Averianov, A. New material of *Caenagnathasia martinsoni* (Dinosauria: Theropoda: Oviraptorosauria) from the Bissekty Formation (Upper Cretaceous: Turonian) of Uzbekistan. *Cretaceous Research* **54**, 50–59 (2015).
82. Archibald, J. D. & Averianov, A. P. Mammalian Faunal Succession in the Cretaceous of the Kyzylkum Desert. *J Mammal Evol* **12**, 9–22 (2005).
83. Redman, C. M. & Leighton, L. R. Multivariate faunal analyses of the Turonian Bissekty Formation: variation in the degree of marine influence in temporally and spatially averaged fossil assemblages. *Palaios* **24**, 18–26 (2009).
84. Brusatte, S. L., Averianov, A., Sues, H.-D., Muir, A. & Butler, I. B. New tyrannosaur from the mid-Cretaceous of Uzbekistan clarifies evolution of giant body sizes and advanced senses in tyrant dinosaurs. *Proceedings of the National Academy of Sciences* **113**, 3447–3452 (2016).
85. Archibald, J. D. *et al.* Précis of the Cretaceous paleontology, biostratigraphy and sedimentology at Dzharakuduk (Turonian?-Santonian), Kyzylkum Desert, Uzbekistan. *Bulletin of the New Mexico Museum of Natural History and Science* **14**, 21–28 (1998).
86. Ward, D., King, C., Morris, N. & Kennedy, W. On some Upper Cretaceous ammonites from western Uzbekistan. *Acta geologica Polonica* **73**, 000–000 (2023).
87. Kurumada, Y. *et al.* Calcite U–Pb age of the Cretaceous vertebrate-bearing Bayn Shire Formation in the Eastern Gobi Desert of Mongolia: Usefulness of caliche for age determination. *Terra Nova* **32**, 246–252 (2020).
88. Bellot, S. *et al.* On the origin of giant seeds: the macroevolution of the double coconut (Lodoicea maldivica) and its relatives (Borasseae, Arecaceae). *New Phytologist* **228**, 1134–1148 (2020).
89. Voltura, E. V. *et al.* Parrot Dietary Habits and Consumption of Alternate Foodstuffs. *Journal of Avian Medicine and Surgery* **37**, (2024).

90. Zanno, L. E. & Makovicky, P. J. Herbivorous ecomorphology and specialization patterns in theropod dinosaur evolution. *Proceedings of the National Academy of Sciences* **108**, 232–237 (2011).
