# Supplementary material for: Fruit-eating adaptations in a Late Cretaceous oviraptorosaur (Theropoda)
Source: Nat Commun. 2026 Oct 10;17:11549. doi: 10.1038/s41467-026-78343-w (PMC13748659; doi:10.1038/s41467-026-78343-w)
Supplement: Supplementary file 2 [file 41467_2026_78343_MOESM2_ESM.pdf]

Description of Additional Supplementary File

Supplementary Data 1 -

READ ME

Data files for Funston et al. "Potential Fruit-eating adaptations in a Late Cretaceous Theropod"

This data archive includes four subfolders:
1) Plot datasets
2) Final phylogenetic trees
3) Input matrices for parsimony-based phylogenetic analysis
4) Input matrices for Bayesian phylogenetic analysis

1_Plot_Datasets:
- This folder contains three files and one folder.
- "Drupe _plot _data.csv" is the size data for drupe-type fruit used in Fig. 4b, taken from Naware and Benson (2024).
- "Naware_Benson_24_K_Fruit_data.csv" is the data for all fruit types used in Fig. 4b, also taken from Naware and Benson (2024).
- "Phylodata _65050 _240614.csv" is age-calibration and phenotypic data for oviraptorosaurs, including first appearance dates, last appearance dates, and body mass estimates. This dataset is built on the one from Funston (2020).
- The subfolder "Muscle Vector Data" includes .csv files of decomposed muscle vector coordinates for individual taxa, as named in the filename. These data were used to produce the plot in Fig. 3b,c.

2_Final_Trees:
- This folder contains four files.
- "65050TNT _strict.phy" is a phylip file of the strict consensus tree for the parsimony-based phylogenetic analysis. It is the tree in Fig. S11a. - "Bayesian_noclock_mrbayes_sumt.phy" is a phylip file of the majority rules tree for the full dataset, analysed using Bayesian techniques but without tip-dating.
- "Funston _Bayes _Matrix231106constr_maxpost.phy" is a phylip file of the maximum posterior probability tree produced by MrBayes software, for the full-dataset Bayesian analysis without tip-dating. This tree was scaled by an a posteriori fossilized birth-death model to produce Fig. 4a, and is the tree in Fig. S11b.
- "Infernogallus _231204 _clock _nodummy.tre" is the summary tree for the 4-rate relaxed-clock tip-dated Bayesian analysis. It is the tree in Fig. S12.

3_Parsimony_Analyses:
- This folder contains two files.
- "Funston _full _matrix240619nex.nex" is the Nexus file for the parsimony analysis with the full matrix.
- "Funston _master _matrix231106.tnt" is the TNT file for the parsimony analysis with the full matrix.

4_Bayesian_Analyses:
- This folder contains four files and four folders.
- "Funston_bayes_matrix231201constr_3rates.nex" is the Nexus matrix for the 3-rate tip-dated analysis. The folder "Infernogallus231201 _clock _3rates" contains the results for this analysis.
- "Funston_bayes_matrix231201noclock.nex" is the Nexus matrix for the trimmed analysis with no clock. The folder "Infernogallus231201_noclock" contains the results for this analysis.
- "Funston_bayes_matrix231204constr_4rates.nex" is the Nexus matrix for the 4-rate tip-dated analysis. The folder "Infernogallus231201 _clock _4rates" contains the results for this analysis.
- "Funston_Fullbayes_matrix231206.nex" is the Nexus matrix for the full matrix with no clock. The folder "Infernogallus_Bayesian_noclock_fullmatrix" contains the results for this analysis.
