# Supplementary material for: Fruit-eating adaptations in a Late Cretaceous oviraptorosaur (Theropoda)
Source: Nat Commun. 2026 Oct 10;17:11549. doi: 10.1038/s41467-026-78343-w (PMC13748659; doi:10.1038/s41467-026-78343-w)
Supplement: Supplementary file 3 [file 41467_2026_78343_MOESM4_ESM.pdf]

nature portfolio

Corresponding author(s): Gregory F. Funston

Last updated by author(s): 2026/07/09

Reporting Summary

Nature Portfolio wishes to improve the reproducibility of the work that we publish. This form provides structure for consistency and transparency in reporting. For further information on Nature Portfolio policies, see our Editorial Policies and the Editorial Policy Checklist.

Statistics

For all statistical analyses, confirm that the following items are present in the figure legend, table legend, main text, or Methods section.

n/a	Confirmed	
☐	☒	The exact sample size (n) for each experimental group/condition, given as a discrete number and unit of measurement
☐	☒	A statement on whether measurements were taken from distinct samples or whether the same sample was measured repeatedly
☒	☐	The statistical test(s) used AND whether they are one- or two-sided<br>*Only common tests should be described solely by name; describe more complex techniques in the Methods section.*
☐	☒	A description of all covariates tested
☒	☐	A description of any assumptions or corrections, such as tests of normality and adjustment for multiple comparisons
☐	☒	A full description of the statistical parameters including central tendency (e.g. means) or other basic estimates (e.g. regression coefficient) AND variation (e.g. standard deviation) or associated estimates of uncertainty (e.g. confidence intervals)
☒	☐	For null hypothesis testing, the test statistic (e.g. F, t, r) with confidence intervals, effect sizes, degrees of freedom and P value noted<br>*Give P values as exact values whenever suitable.*
☐	☒	For Bayesian analysis, information on the choice of priors and Markov chain Monte Carlo settings
☒	☐	For hierarchical and complex designs, identification of the appropriate level for tests and full reporting of outcomes
☒	☐	Estimates of effect sizes (e.g. Cohen's d, Pearson's r), indicating how they were calculated

Our web collection on statistics for biologists contains articles on many of the points above.

Software and code

Policy information about availability of computer code

Data collection	No software or code was used to collect data in this study. Mesquite v 4.02 was used to compile and organize the phylogenetic matrix (characters were scored manually).
Data analysis	RStudio v3.6.1 was used for all statistical analyses. Packages used include MuscleTenary, ggplot2, ggpubr, ggsci, gridExtra, phytools, viridis, lmodel2, strap, Claddis, smatr, geiger, caper, nlme, and MCMCglmm. No custom code was used for data analysis. For phylogenetic analysis, TNT v 1.5 and Mr Bayes v. 3.2.7 were used. Phylogenetic scripts are available as supplementary files. Slicer v4.10.2 was used for µCT segmentation, Meshlab v 2020.06 was used for mesh alignment, and Blender v. 3.1.2 was used for retrodeformation. NIF Elements (no version number) was used for palaeohistological imaging. Figures were assembled in Adobe Photoshop 2026.<br><br>Phylogenetic analysis scripts are available in the Supplementary Data Files hosted at Figshare [https://doi.org/10.6084/m9.figshare.31901296]. Four directories and a ReadMe file are provided in the .zip archive. No custom code or software was used in the study.

For manuscripts utilizing custom algorithms or software that are central to the research but not yet described in published literature, software must be made available to editors and reviewers. We strongly encourage code deposition in a community repository (e.g. GitHub). See the Nature Portfolio guidelines for submitting code & software for further information.

Data

Policy information about availability of data

All manuscripts must include a data availability statement. This statement should provide the following information, where applicable:

- Accession codes, unique identifiers, or web links for publicly available datasets
- A description of any restrictions on data availability
- For clinical datasets or third party data, please ensure that the statement adheres to our policy

Fossil specimens in this study are permanently deposited at the Royal Ontario Museum (ROM VP 65050, ROM VP 64194, and ROM VP 88690) and North Dakota Geological Survey (NDGS 4351), where they can be accessed by contacting the relevant curator. Data used to conduct this study (including all Source files for figures) are available as Supplementary Data files (Supplementary Data 1). The µCT, phylogenetic and quantitative data generated in this study, including all data and code used to generate the figures, have been deposited in the Figshare database under accession code NCOMMS-26-001683A105 [https://doi.org/10.6084/m9.figshare.31901296].

Field-specific reporting

Please select the one below that is the best fit for your research. If you are not sure, read the appropriate sections before making your selection.

☐ Life sciences ☐ Behavioural & social sciences ☒ Ecological, evolutionary & environmental sciences

For a reference copy of the document with all sections, see nature.com/documents/nr-reporting-summary-flat.pdf

Ecological, evolutionary & environmental sciences study design

All studies must disclose on these points even when the disclosure is negative.

Study description	The research consisted of 1) anatomical study of the specimens; 2) µCT scanning, segmentation, and modelling; 3) biomechanical analysis via calculation of muscle vectors; 4) phylogenetic analysis using parsimony and Bayesian methods; and 5) palaeohistological analysis via thin sections. Statistical analyses included principal components analysis on scaled muscle vector data, but there were no treatments of data, experimental units, or replicates.
Research sample	The research sample comprises four focal fossil specimens of the new species, collected from Montana and North Dakota, and comparative specimens housed at institutes around the world. In addition, we used a previously published dataset from Naware and Benson (2024)
Sampling strategy	The sample size and inclusion was dictated by the number of fossils discovered and their quality.
Data collection	Fossils were collected according to local permitting requirements. ROM VP 65050, VP 64193, VP 64194, and VP 88690 were collected from private land with the landowner's permission. NDGS 4351 was collected under BLM permit MTM 108845 during a field trip for the Cretaceous and Beyond symposium held in Dickinson, North Dakota in 2019. Further details are available to researchers upon request.<br><br>Specimen measurements were collected by GFF using calipers or a measuring tape and recorded using a mechanical pencil and paper, or compiled directly in Microsoft Excel. Anatomical study of the specimen was conducted by GFF with input from DCE and DMD. Palaeohistological data were collected by producing a thin-section using standard methods followed by polarized light microscopy. Phylogenetic data were collected by first-hand observation of fossil specimens by GFF, or from the literature. µCT data was collected as described in the methods, and was segmented by GFF. Biomechanical data was collected as outlined in the methods.
Timing and spatial scale	Specimens come from the late Maastrichtian Hell Creek Formation, approx 68–66 mya, and were discovered in Montana and North Dakota. Detailed locality information is provided in the Supplementary Information
Data exclusions	No relevant data were excluded from the study
Reproducibility	Replication is not applicable to the study because there were no experimental groups.
Randomization	Randomization was not applied because there were no experimental groups.
Blinding	Blinding was not used because there were no experimental groups.

Did the study involve field work? ☒ Yes ☐ No

Field work, collection and transport

Field conditions	Fieldwork was carried out by DCE to measure stratigraphic section from the holotype site. Detailed locality information is provided below.
Location	Detailed locality information for all the specimens is provided below.

Access & import/export	All fieldwork and specimen collection adhered to local laws, including permission from private landowners and permits issued by the Bureau of Land Management in the USA.
Disturbance	No ecological disturbance was caused by the fieldwork, as it took place on foot in un-vegetated badlands and involved removal of minimal sediment.

Reporting for specific materials, systems and methods

We require information from authors about some types of materials, experimental systems and methods used in many studies. Here, indicate whether each material, system or method listed is relevant to your study. If you are not sure if a list item applies to your research, read the appropriate section before selecting a response.

Materials & experimental systems

n/a	Involved in the study
☒	☐ Antibodies
☒	☐ Eukaryotic cell lines
☐	☒ Palaeontology and archaeology
☒	☐ Animals and other organisms
☒	☐ Human research participants
☒	☐ Clinical data
☒	☐ Dual use research of concern

Methods

n/a	Involved in the study
☒	☐ ChIP-seq
☒	☐ Flow cytometry
☒	☐ MRI-based neuroimaging

Palaeontology and Archaeology

Specimen provenance

All five specimens are from the Maastrichtian Hell Creek Formation (see detailed locality information below). This land is the traditional territory and home of the Niitsítapi; Očhéthi Šakówiŋ; Apsáalooke; Tséstho’e; Cayuse, Umatilla, and Walla Walla; and Métis peoples, who have been stewards of these areas since time immemorial. All specimens were collected in accordance with local permitting requirements, and are permanently accessioned at accredited museums where they are available for study in perpetuity. Initial preparation of ROM VP 65050 was conducted by CK Preparations (Montana) prior to acquisition by the ROM in Apr2012, but no reconstructive work was done, and final preparation was carried out by ROM staff (S. Sugimoto, I. Morrison). CT scans confirm the authenticity of the specimen, including the skull.

Detailed Locality Information:

ROM VP 65050— The holotype material was collected from private, deeded land in the northeastern part of Garfield County, Montana, USA, by C. Phipps in July 2011. Detailed location information is on file at the Royal Ontario Museum. The specimen was preserved in a concretion within an organic-rich, medium-grained sandstone approximately 3.75 m thick (Supplementary Fig. 1). The site is 15.5 m below the K/Pg boundary (measured with a Jacob’s staff by DCE), which is approximated by the base of a 4 m-thick coal swarm exposed on top of a ridge ~1 km to the NE. The specimen was acquired by the ROM in April, 2012 with a ROM Strategic Acquisition Grant to DCE from the Louise Hawley Stone Charitable Trust. Detailed location information, including exact GPS coordinates, is on file at the Royal Ontario Museum, as is a letter from the BLM stating its provenance from private, deeded land.

ROM VP 64193 and 64194—These two specimens, comprising partial dentaries and an articular-surangular-coronoid complex, were collected together from private land the south-central part of Garfield County, Montana, USA. The specimens were collected from the same channel complex that produced the holotype of Acheroraptor temertyorum 93. The specimens are identical in preservation style and correspond to an animal of the same size, and therefore we interpret them to belong to the same individual. Detailed location information is on file at the Royal Ontario Museum.

ROM VP 88690—ROM VP 88690 was acquired by the ROM in 2015. It was collected from the Hell Creek Formation exposed near Faith, South Dakota. Detailed location and provenance information is on file at the Royal Ontario Museum.

NDGS 4351—NDGS 4351 was collected in 2019 by one of the authors [DF] from locality NDGS L10006 (=PTRM V87012 94) in the Mud Buttes area of southwestern Bowman County, North Dakota on lands managed by the Bureau of Land Management. It was collected under BLM permit MTM 108845 during a field trip for the Cretaceous and Beyond symposium held in Dickinson, North Dakota in 2019. Further details are available to researchers upon request. The specimen was collected from a microvertebrate accumulation preserved within a silty mudstone interpreted as a floodplain environment within the upper portion of the Hell Creek Formation 9.85 meters below the Cretaceous-Paleogene boundary 94 (Pearson et al., 2002), Lancian North American Land Mammal Age, within plant zone HCIII 95.

Specimen deposition	Specimens are housed in either the Royal Ontario Museum, Toronto, ON, Canada(ROM VP 65050, ROM VP 64193, ROM VP 64194, and ROM VP 88690; or the North Dakota Geological Survey, Bismarck, ND, USA (NDGS 4351).
Dating methods	No new dates are provided

☒ Tick this box to confirm that the raw and calibrated dates are available in the paper or in Supplementary Information.

Ethics oversight	No ethical approval or guidance required for the study, as it used specimens already housed in institutions.

Note that full information on the approval of the study protocol must also be provided in the manuscript.
