# Supplementary material for: Fruit-eating adaptations in a Late Cretaceous oviraptorosaur (Theropoda)
Source: Nat Commun. 2026 Oct 10;17:11549. doi: 10.1038/s41467-026-78343-w (PMC13748659; doi:10.1038/s41467-026-78343-w)
Supplement: Supplementary file 4 [file 41467_2026_78343_MOESM5_ESM.pdf]

nature portfolio

Peer Review File

Fruit-eating adaptations in a Late Cretaceous oviraptorosaur (Theropoda)

Corresponding Author: Dr Gregory Funston

This file contains all reviewer reports in order by version, followed by all author rebuttals in order by version.

Version 0:

Reviewer comments:

Reviewer #1

(Remarks to the Author)
This manuscript reports on a new genus of caenagnathid oviraptorosaur from the Hell Creek Formation of North Dakota. The most remarkable part of the specimen is its skull, which is highly complete by caenagnathid standards. Thus, it is an important addition to the research on this group of oviraptorosaurs. The description of this specimen is well presented, and the diagnosis is convincing. The authors place their major focus on the biomechanical and ecological implications derived from the skull. The argument is logically coherent, combining muscle topology, joint mobility, and occlusal morphology to infer jaw function and diet. Overall, the manuscript presents an important specimen and provides a hypothesis on one of the key unsolved issues in dinosaur paleontology—the link between herbivorous dinosaurs and the rise of flowering plants in the Cretaceous—which I believe should be published once some minor issues are addressed.

1. The estimate of "at least 15 mm anteroposterior jaw motion" likely represents an overestimate of the functional range of motion. While the authors note that "soft tissues likely affected true joint mobility," osteological range of motion calculations provide a maximum bony limit, and soft tissues likely restrict actual motion within this range. I suggest rephrasing this part (e.g., up to ~15 mm) to better reflect this idea.

2. Figure 3: It would be great to standardise Non-oviraptorid "t"heropods with "Sliding jaw joint".

3. Figure 3: I suggest refining the legend of the figure to make it more easily understandable. The symbols for Oviraptorosauria and Aves can likely be presented without the square outline, allowing the black square and circle to be enlarged to match the size of the triangle.

Reviewer #2

(Remarks to the Author)
Dear Authors,

I had the opportunity to review the manuscript entitled, "Fruit-eating adaptations in a Late Cretaceous theropod". The manuscript describes a new species of caenagnathid theropod dinosaurs, and identifies key features that the authors argue are associated with frugivory.

As I am not a taxonomist, I will have to trust the authors on the systematics of the new specimen.

What I can comment on are the functional morphology, muscle reconstructions, and ecological interpretations.

While I have no problems with the functional morphological interpretations and muscle reconstructions per se, the ecological conclusions presented here largely are qualitative in nature and not supported by any statistical analyses. Strictly speaking, the ternary plot and PCA morphospaces are not statistical tests - so the interpretations of these plots are also qualitative. This means the conclusions of the manuscript are not probabilistic predictions supported by statistical tests.

This can be resolved by conducting statistical predictive modelling such as discriminant analyses, but in a phylogenetic framework to account for the undesired effects of shared ancestry (Felsenstein 1985; Harvey and Pagel 1991).

Perhaps a minor point but because the muscle vectors are presented as cylinders, it gives the impression that some kind of dynamic analyses like multibody dynamic analysis or gape analyses were conducted. Given that the muscle vectors were reconstructed in 2D and neither muscle forces nor gapes were analysed, it would be less misleading if simple 2D lines or arrows were used instead cylinders.

Overall I think the manuscript is somewhat confusing as written as one needs to go to the supplement to understand exactly what was and was not conducted. The manuscript should stand on its own.

References:

Felsenstein, J. 1985. 'Phylogenies and the Comparative Method'. Am Nat 125 (1): 1–15. https://doi.org/Doi%252010.1086/284325.

Harvey, P. H., and M. D. Pagel. 1991. The Comparative Method in Evolutionary Biology. Oxford University Press.

Reviewer #3

(Remarks to the Author)
I am not an expert on these particular dinosaur group but the description and conclusions drawn from the morphology sound plausible and coherent to me.
The conclusions drawn from osteohistology are also correct. The osteohistological description is very short but include all necessary factors and document skeletal maturity, an advanced ontogenetic stage of the specimen. I would not described the vascular denisty as dense, its more moderate. A detailed/enlarged figure of the bone matrix in polarized light added to the suppl. figure would be nice.
Sincerely

Version 1:

Reviewer comments:

Reviewer #1

(Remarks to the Author)
Thank you for addressing the concerns raised in the previous round of review. I think the manuscript is now ready for publication.

Reviewer #2

(Remarks to the Author)
Dear authors,

I was Reviewer #2 in the previous iteration of peer review.

I want to thank the authors for considering my suggestion of running a phylogenetically-informed LDA to predict diet in Infernogallus.

Just a point of slight disagreement on how phylogeny and LDA would conflict in terms of their contributions to the VCV - they can be accounted for simultaneously in phylogenetic implementations. It just means that the VCV will be scaled according to any phylogenetic structure, and that would then be used for the LDA. Maybe the lackluster performance of the pFDA was because of the small sample size - it should work in theory given a big enough sample size. It appears sample size is a bigger issue than statistical non-independence owing to phylogeny in this instance, so I concede that qualitative interpretation is the sensible approach here.

I agree that it would be confusing for the readers but it would equally be a shame to waste all the effort that went into this so I think it would be great to see this in the supplementary material. Plus, there would be some who would argue that a phylogenetic approach is necessary, so it would be beneficial for such readers if these analyses were included in the supplement - but I would emphasise the issue of sample size over phylogenetic non-independence and that a full-scale analysis on extant birds would be far beyond the scope of this study.

I am overall satisfied with the responses and amendments made with respect to my comments.

One new comment I do have relates to the Bayesian phylogenetic inferences: I'm not quite sure if I'm just missing something but it's a bit unclear on the details of the first Bayesian phylogenetic inference. Is it a conventional birth-death model? The description of this model contrasts with the level of detail that's gone into the second Bayesian inference (the FBD model) so perhaps a bit more detail would improve the manuscript.

Lastly, I just want to say that I really love the name Infernogallus. Hell chicken is such a great name!

Best wishes,
Manabu

Open Access This Peer Review File is licensed under a Creative Commons Attribution 4.0 International License, which permits use, sharing, adaptation, distribution and reproduction in any medium or format, as long as you give appropriate credit to the original author(s) and the source, provide a link to the Creative Commons license, and indicate if changes were made.
In cases where reviewers are anonymous, credit should be given to 'Anonymous Referee' and the source.
The images or other third party material in this Peer Review File are included in the article's Creative Commons license, unless indicated otherwise in a credit line to the material. If material is not included in the article's Creative Commons license and your intended use is not permitted by statutory regulation or exceeds the permitted use, you will need to obtain permission directly from the copyright holder.
To view a copy of this license, visit https://creativecommons.org/licenses/by/4.0/

Dear reviewers,

Thank you for your time and effort in assessing our manuscript. We are very pleased to see the positive responses of the reviewers and their constructive suggestions for improvement. We have taken these to heart and have revised our manuscript accordingly. This includes some revisions to the text (in addition to the formatting changes you outlined), as well as additions to the figures to make legends clearer and to present more histological data.

Regarding the statistical tests requested by the second reviewer: we discuss those tests at length in this response document, showing that where they can be performed, they support our hypothesis, but arguing that they are not fully appropriate to tackle the question at hand. We have revised the text of the manuscript to make this clearer to the reader. We are happy to include a refined version of this additional discussion and results in the supplementary material of the manuscript, should you wish.

To make our revisions easier to keep track of, we have provided a Microsoft Word document with 'Tracked changes', highlighting differences, and we have also responded to each of the reviewers' points below.

Reviewer #1 (Remarks to the Author):

This manuscript reports on a new genus of caenagnathid oviraptorosaur from the Hell Creek Formation of North Dakota. The most remarkable part of the specimen is its skull, which is highly complete by caenagnathid standards. Thus, it is an important addition to the research on this group of oviraptorosaurs. The description of this specimen is well presented, and the diagnosis is convincing. The authors place their major focus on the biomechanical and ecological implications derived from the skull. The argument is logically coherent, combining muscle topology, joint mobility, and occlusal morphology to infer jaw function and diet. Overall, the manuscript presents an important specimen and provides a hypothesis on one of the key unsolved issues in dinosaur paleontology—the link between herbivorous dinosaurs and the rise of flowering plants in the Cretaceous—which I believe should be published once some minor issues are addressed.

Response: Thank you for your support of the manuscript!

1. The estimate of "at least 15 mm anteroposterior jaw motion" likely represents an overestimate of the functional range of motion. While the authors note that "soft tissues likely affected true joint mobility," osteological range of motion calculations provide a maximum bony limit, and soft tissues likely restrict actual motion within this range. I suggest rephrasing this part (e.g., up to ~15 mm) to better reflect this idea.

Response: Good idea – we want to make this as clear to readers as possible. We agree that this is likely an overestimate of actual motion, although there are joints for which soft tissue actually *enhances* the range of motion over what would be expected based on bony surfaces. Nevertheless, we have rephrased this part according to your suggestion.

2. Figure 3: It would be great to standardise Non-oviraptorid "t"heropods with "Sliding jaw joint".

Response: Good catch – we have standardized the capitalization in the legend.

3. Figure 3: I suggest refining the legend of the figure to make it more easily understandable. The symbols for Oviraptorosauria and Aves can likely be presented without the square outline, allowing the black square and circle to be enlarged to match the size of the triangle.

Response: We have taken your suggestion on board. We initially included the outlines to represent the convex hulls around each of those groups, but because they are represented by symbols, it should already be clear to readers what those hulls represent. The icons are now all the same size.

Reviewer #2 (Remarks to the Author):

Dear Authors,

I had the opportunity to review the manuscript entitled, "Fruit-eating adaptations in a Late Cretaceous theropod". The manuscript describes a new species of caenagnathid theropod dinosaurs, and identifies key features that the authors argue are associated with frugivory.

As I am not a taxonomist, I will have to trust the authors on the systematics of the new specimen.

What I can comment on are the functional morphology, muscle reconstructions, and ecological interpretations.

Response: Thanks for making clear the nature of your review, and for your valuable comments on the ecological interpretations.

While I have no problems with the functional morphological interpretations and muscle reconstructions per se, the ecological conclusions presented here largely are qualitative in nature and not supported by any statistical analyses. Strictly speaking, the ternary plot and PCA morphospaces are not statistical tests - so the interpretations of these plots are also qualitative. This means the conclusions of the manuscript are not probabilistic predictions supported by statistical tests.

This can be resolved by conducting statistical predictive modelling such as discriminant analyses, but in a phylogenetic framework to account for the undesired effects of shared ancestry (Felsenstein 1985; Harvey and Pagel 1991).

Response: We are happy to hear that you agree with our interpretations, and we likewise agree that our methods do not constitute statistical tests. This is something that we grappled with back and forth during our analysis, which we reflected in a passage in the supplement to that effect, about the "the difficulty of distilling the complexity of muscle function, joint morphology, and behaviour into easily quantifiable parameters". These are highly complex systems, and we found through our analytical process that muscle vectors were the best (but still imperfect) reflection of diet that could be comparable across these clades. As we discuss in the supplement, the dietary signal in the muscles is complicated by the fundamental differences in cranial architecture between birds and their theropod ancestors, as well as vagueness in dietary categories, and the relatively few times that flesh-processing frugivory has evolved in birds, limiting comparative replicates and statistical power. These are barriers which a human observer can easily tease apart and interrogate, but which are difficult to do for a computer, especially at the relatively small sample sizes that are dictated by our data. Indeed, visualization of our data using the ternary plots and PCAs clearly demonstrates a trend: although birds are distinct from non-avian dinosaurs, *Infernogallus* demonstrates a muscle vector organization that is derived from other taxa. In this way, *Infernogallus* morphologically converges with some birds, and is closest to those that eat soft plant material. Although this is clear to an observer who is able to see the context of the plots, because of the separate spaces occupied by the birds and dinosaurs, it is difficult to apply statistics to this trend.

Nevertheless, we wanted to fully engage with your suggestion, so we did undertake some further statistical work as well, which we present below.

Although we appreciate your suggestion to use a phylogenetically-corrected discriminant analysis, perhaps unintuitively, it is not straightforward to combine phylogenetic correction and linear discriminant analysis. This is because both approaches interact with the variance-covariance (vcv) matrix of a regression as a key part of their data-handling approach. Whereas phylogenetic correction scales the vcv by branch lengths in the tree, an LDA modifies the vcv to maximize the variation in regressions between the *a priori* groups. Although these could in theory be combined, the resulting vcv would not accomplish either task desired at the outset. There are some published approaches that overcome this barrier, but most are designed for continuous variables rather than the categorial variables we would need to use (diet categories).

So, to examine statistical support for our hypothesis, we used three different approaches, outlined below. We used the following dietary categories for the birds in our dataset: "soft" for soft plants (*Psittrichas*, *Anas*, *Chauna*); "seed" for granivores that processed seeds (*Psittacus*, *Geospiza*); "omni" for omnivorous taxa or those with very broad diets (*Gallus, Struthio, Corvus, Eudromia*); and "meat" for carnivores (*Buteo*). In the graphs below, the 'points' are the dietary category for each taxon as a word, rather than a point or the species name – hopefully it is still possible to make sense of the graphs.

1) **The first approach was a phylogenetic flexible discriminant analysis (pFDA), based on the approach from Schmitz & Motani (2011). For this approach, we categorized the birds as above, and left all the dinosaur taxa as 'unknown'. This approach does a very poor job of discriminating between dietary categories in the extant birds (3 of 10 misclassified in the training set), and predicts all theropods as "omnivores" with exceedingly high certainty. Infernogallus is the least strongly predicted as an omnivore (94.3%), with the remainder of likelihood made up by "soft" plants.**

2) **The second approach was to conduct a conventional LDA, not taking into account phylogenetic relatedness. We performed the LDA on the extant birds for which diet was known, and then predicted dinosaurs into the space. This approach only minimally reorganized the bird data points compared to the PCA (main text Fig. 3c), demonstrating that the PC axes are generally as effective at visualizing the dietary categories as the LDA axes. Estimating the diet of the dinosaurs in our dataset based on this analysis predicts all of them to be omnivores with >85% probability, with the exception of *Infernogallus*, which comes out as only 75% probable to be an omnivore, with the remaining 25% proportion of likelihood being "soft" foods. Visual assessment of the data shows that this pattern is driven by all of the dinosaurs plotting highly negatively along LD2, in their own region of space, just like in the PCA, again with the exception that *Infernogallus* plots among the birds.**

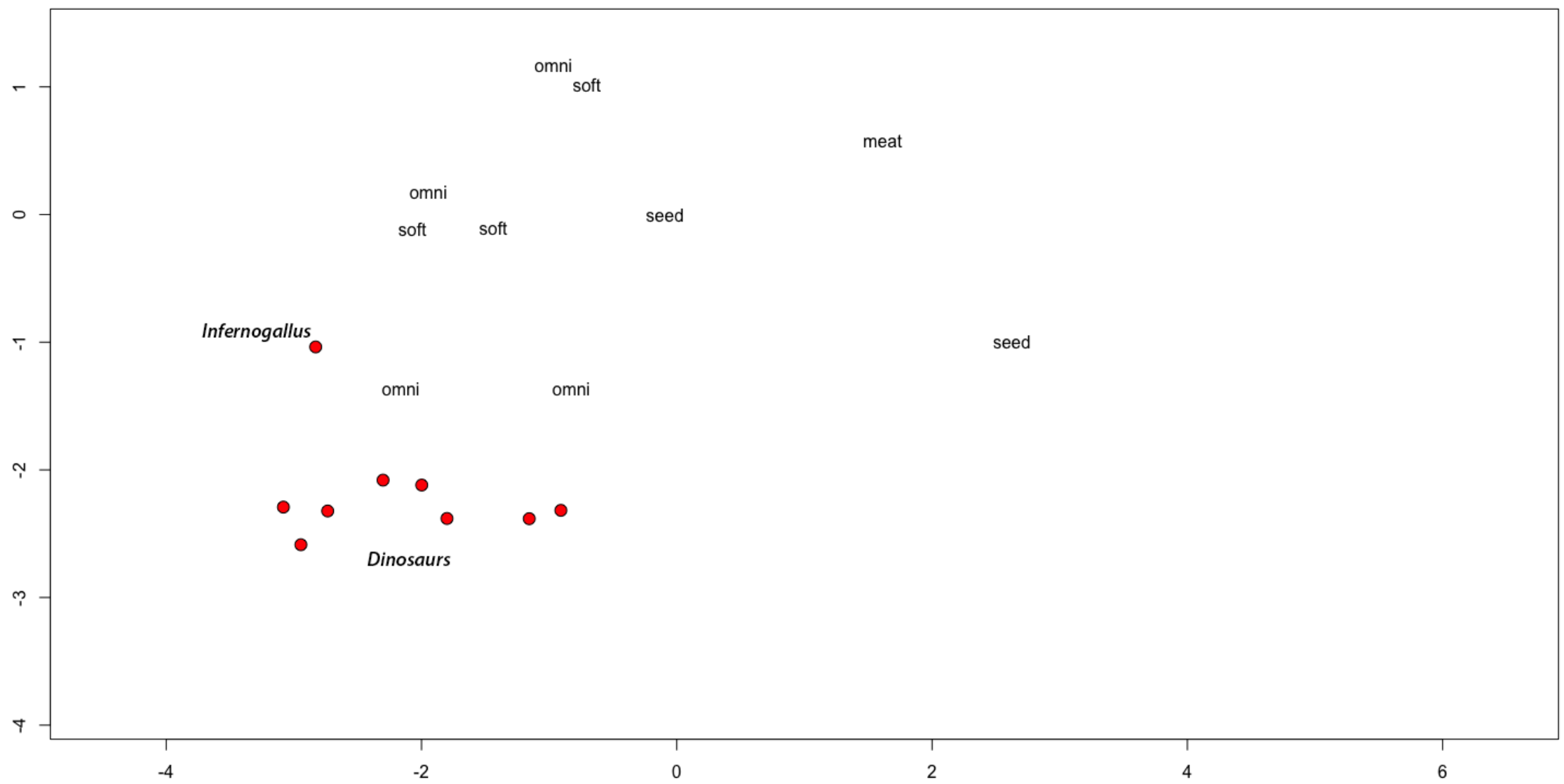

Whereas this supports the idea that *Infernogallus* is as well adapted for soft food as any dinosaur in our dataset, it also shows that the highly derived morphologies of birds are probably not easily translatable to theropod diets without more detailed comparisons.

3) **To incorporate phylogeny into the analysis, we conducted a phylogenetically-informed PCA (pPCA) and then performed an LDA on the resulting PC values for each taxon. This allowed us to structure the data using a phylogeny, but then allow the LDA to control the vcv. We did this analysis in two main ways, as well as some trial runs of intermediate approaches:**
   a. **The 'main' analysis was one in which we performed the pPCA on all of the taxa in our dataset, using the phylogeny and node ages of Prum et al. 2015 for birds, and consensus estimates of node ages for dinosaurs. We then performed an LDA using only the birds, with the same dietary categories as above. Then we predicted the dinosaurs into this space. This produced results exactly identical to the non-phylogenetically-corrected analysis, except the polarity of the axes was flipped. This indicates that the relatedness of dinosaurs and birds to each other is not affecting the signal to a great degree. It also reflects the way that LDA overprints the vcv produced by a phylogenetic correction – while the PC axes may change, the LDA still finds the underlying structure.**

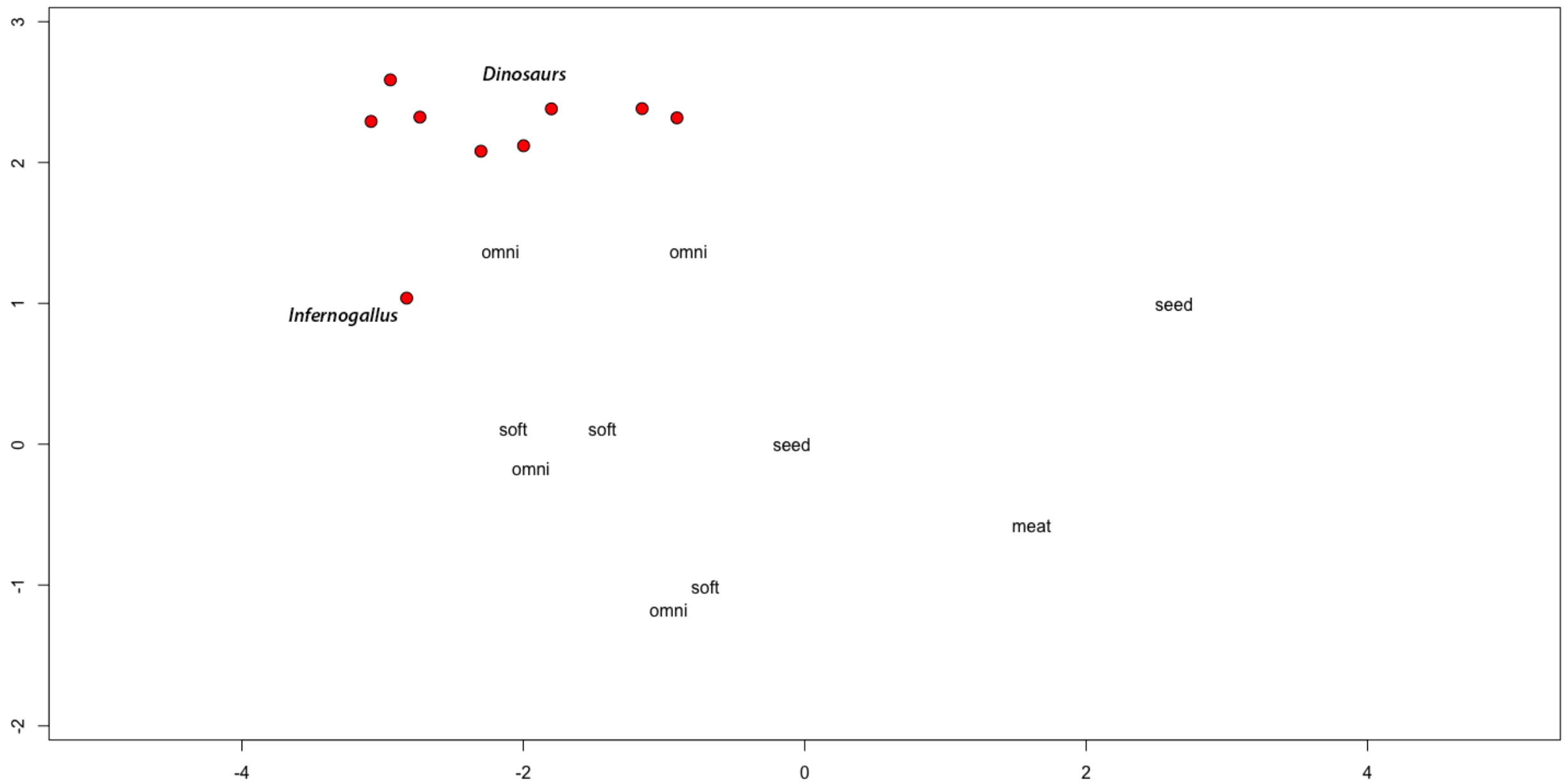

b. **The ‘exploratory’ analysis was one in which we used generally-agreed-upon diets for the non-oviraptorosaur dinosaurs in the LDA, to flesh out the sample and predict diets for the oviraptorosaurs. For this, we used the bird diets as normal, and we categorized *Tyrannosaurus*, *Allosaurus*, and *Dromaeosaurus* as meat-eaters, and *Erlikosaurus* as a “soft” plant eater (as it has generally been assumed to eat foliage rather than handling seeds). This analysis, again, did not substantially rearrange the points, reinforcing that the PCA and LDA are playing with the same axes of variation. Whereas this is unsurprising given that we only have 3 variables (because they are 3-dimensional muscle vectors), it also reinforces that the PC axes do a good job of visualizing the dietary categories, and that dietary categories are indeed reflected to some degree by the muscle vectors. When predicting the oviraptorosaurs into the LDA space, the oviraptorids and *Incisivosaurus* are predicted to be meat-eaters, whereas *Infernogallus* is predicted to be a soft-plant eater. *This directly supports our hypothesis that* Infernogallus *was adapted for eating soft plant materials like fruits,* but we choose to leave it from the manuscript due to issues in assuming fossil diets are known. However, it is noteworthy that although the “soft” category is the most favoured prediction for *Infernogallus*, it is only slightly more likely than the omnivorous category (40.6% vs. 40.3%)—these categories overlap extensively in the birds. The other oviraptorosaurs are much more imbalanced, with meat being favoured over other categories by >9%.**

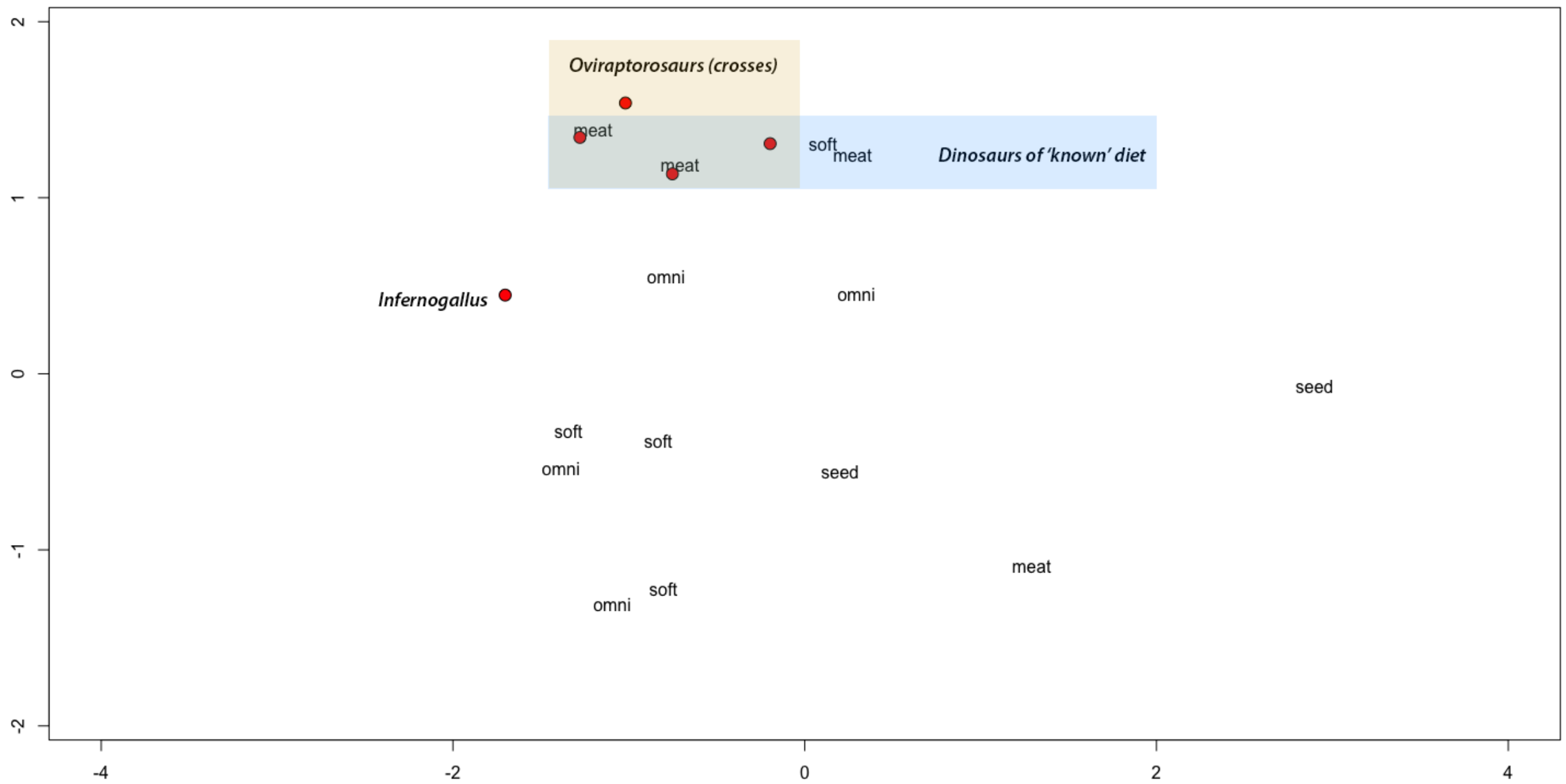

To summarize the results above, the statistical analyses generally corroborate two ideas already demonstrated in the manuscript: 1) *Infernogallus* had significantly different muscle organization than other theropods, even other oviraptorosaurs; and 2) it is difficult to distil a single index that adequately reflects dietary behaviour in birds, probably as a consequence of both data availability (i.e., lack of detailed behavioural or dietary data), and actual complexity in the controls on adductor muscle vector orientation. Critically, none of these analyses provide any meaningful signal that contradict our PCA interpretation, and where they are ambiguous, it is clear that it is caused by limitations in the dataset. For these reasons, and to avoid confusing the readers, we have decided not to include these statistical analyses in the main text of the manuscript. Furthermore, while the statistics do indeed support our claims, particularly the final comparison, we feel that it could be misleading to cite them in support of our interpretations, because it might falsely imply confidence in these statistical results. To be clear, we believe that with a larger and more detailed extant dataset (far beyond what we can accomplish here), it is possible a clearer signal could emerge, but we feel the stats are not robust enough for conclusion, even though they seem to support our qualitative claim.

Should you wish, we can include discussion to this effect as supplementary material to the manuscript, alongside more polished versions of the graphs (I'm sorry they are basic but I thought they were still illustrative).

Perhaps a minor point but because the muscle vectors are presented as cylinders, it gives the impression that some kind of dynamic analyses like multibody dynamic analysis or gape analyses were conducted. Given that the muscle vectors were reconstructed in 2D and neither muscle forces nor gapes were analysed, it would be less misleading if simple 2D lines or arrows were used instead cylinders.

Response: This comment highlights a major typo in the manuscript, because the muscle vectors were indeed reconstructed in three dimensions (hence the ternary plots), not two! That statement was from a very old section of the methods that I realize we had not updated since performing that work (now almost 2 years ago…). While it is true that we did not analyse forces or gapes quantitatively, we feel that cylinders are the best way to represent the muscle vectors because they exist in all three axes – even if the transverse axis is a very small contribution to the overall vector. To address this issue, we have corrected the typo in the methods to explain that these are reconstructed in three dimensions, and this should also be helped by the methods section moving back to the main text.

Overall I think the manuscript is somewhat confusing as written as one needs to go to the supplement to understand exactly what was and was not conducted. The manuscript should stand on its own.

Response: Thanks for this suggestion – you will be happy to hear that it was simply a consequence of the manuscript's formatting for an initial submission to a different venue, which required methodology to live in the supplemental information. We have now moved this to the main text now in accordance with Nature

Communications' formatting standards and it should be much easier for the reader to reference what we've done. We have also tried to do a better job of signposting which methods were used where, including additional in-text references to the methods.

References:

Felsenstein, J. 1985. 'Phylogenies and the Comparative Method'. Am Nat 125 (1): 1–15. https://doi.org/Doi%252010.1086/284325.

Harvey, P. H., and M. D. Pagel. 1991. The Comparative Method in Evolutionary Biology. Oxford University Press.

Reviewer #3 (Remarks to the Author):

I am not an expert on these particular dinosaur group but the description and conclusions drawn from the morphology sound plausible and coherent to me.

Response: We are pleased to hear that you find our conclusions reasonable.

The conclusions drawn from osteohistology are also correct. The osteohistological description is very short but include all necessary factors and document skeletal maturity, an advanced ontogenetic stage of the specimen. I would not described the vascular denisty as dense, its more moderate. A detailed/enlarged figure of the bone matrix in polarized light added to the suppl. figure would be nice.

Response: We are glad to hear you agree with our histological interpretations. This was drafted as a very short addition because we initially submitted to venues with restricted word counts. However, we have more space at Nature Communications so we have now expanded this description, including the addition of a cross-polarized image of the femur to Fig. 1, with the full section shown in Supplementary Figure S7. This should provide more comprehensive documentation for other future work.

Sincerely

Dear Reviewers,

Thank you again for your time and effort in assessing our manuscript. We are very pleased with your positive responses, and a second set of constructive suggestions for improvement. We have implemented these suggestions, including adding the statistical tests to the supplementary material (with a new accompanying figure), signposting these tests in the main text, and clarifying some aspects of the phylogenetic analysis.

In addition to these changes, we have done some minor polishing, including adding a citation to an exciting recent paper that further supports our claim, showing that fruits appropriate for caenagnathids were both more common and larger in the Late Cretaceous than previously recognized (https://www.science.org/doi/10.1126/science.adw9457). All of these changes are highlighted in the revised manuscript using the 'Track Changes' feature of Microsoft Word. Below we respond point-by-point to your suggestions.

REVIEWER COMMENTS

Reviewer #1 (Remarks to the Author):

Thank you for addressing the concerns raised in the previous round of review. I think the manuscript is now ready for publication.

Response: Thank you for your support and your investment of time in assessing our manuscript!

Reviewer #2 (Remarks to the Author):

Dear authors,

I was Reviewer #2 in the previous iteration of peer review.

I want to thank the authors for considering my suggestion of running a phylogenetically-informed LDA to predict diet in Infernogallus.

Response: This was a great suggestion and it was worthwhile for us to explore this avenue, so thank you for providing it.

Just a point of slight disagreement on how phylogeny and LDA would conflict in terms of their contributions to the VCV - they can be accounted for simultaneously in phylogenetic implementations. It just means that the VCV will be scaled according to any phylogenetic structure, and that would then be used for the LDA. Maybe the lackluster performance of the pFDA was because of the small sample size - it should work in theory given a big enough sample size. It appears sample size is a bigger issue than statistical non-independence owing to phylogeny in this instance, so I concede that qualitative interpretation is the sensible approach here.

Response: Thanks for clarifying this point. This does make sense, and it seems that our phylogenetic PCA → LDA pipeline essentially mirrors that implementation. It likely is the case that our small sample size (dictated by the availability of dissections and 3D models) is the major limitation leading to the identical results. We are glad to hear that you agree that our qualitative approach is appropriate here.

I agree that it would be confusing for the readers but it would equally be a shame to waste all the effort that went into this so I think it would be great to see this in the supplementary material. Plus, there would be some who would argue that a phylogenetic approach is necessary, so it would be beneficial for such readers if these analyses were included in the supplement - but I would emphasise the issue of sample size over phylogenetic non-independence and that a full-scale analysis on extant birds would be far beyond the scope of this study.

Response: This is a good point, so we have added the analyses from our response to reviews to the supplementary materials. We present refined text outlining our approaches and interpretations, and highlighting where we feel there are still limitations (primarily sample size but also some biological aspects of the challenge). We have outlined that fleshing out this avenue in a future study would likely provide more clarity, but that this is beyond the scope of this study. This results in the addition of a new supplementary figure presenting the graphs. We have also signposted this in the main text, with a direction for readers to the supplement.

I am overall satisfied with the responses and amendments made with respect to my comments.

Response: Thank you for your support!

One new comment I do have relates to the Bayesian phylogenetic inferences: I'm not quite sure if I'm just missing something but it's a bit unclear on the details of the first Bayesian phylogenetic inference. Is it a conventional birth-death model? The description of this model contrasts with the level of detail that's gone into the second Bayesian inference (the FBD model) so perhaps a bit more detail would improve the manuscript.

Response: We have clarified that this first approach is using Bayesian optimality criteria for our tree search, without any kind of time calibration. This is essentially the approach outlined in Carr & Brusatte's 2016 study of tyrannosauroid phylogeny (https://www.nature.com/articles/srep20252.pdf), but with some updated priors and no experimentation with the gamma parameter.

Lastly, I just want to say that I really love the name Infernogallus. Hell chicken is such a great name!

Response: Thank you! I wish I could take credit for it, but it was a colleague who suggested it – which reminds me that I should have added that detail to the acknowledgments! We've done that now.

Best wishes,
Manabu
